# Supplementary material for: CRISPR-Cas9-producing probiotic bacteria for editing NOX2/gp91phox and treating inflammatory bowel disease
Source: Cell Rep Med. 2026 Jul 24;7(8):102940. doi: 10.1016/j.xcrm.2026.102940 (PMC13522793; doi:10.1016/j.xcrm.2026.102940)
Supplement: Supplementary file 1 [file mmc1.pdf]

**Cell Reports Medicine, Volume *7***

## Supplemental information

# CRISPR-Cas9-producing probiotic bacteria for editing NOX2/gp91$^{phox}$ and treating inflammatory bowel disease

**Chen Zhang, Tianyu Li, Huoye Hao, Gang Wang, Yuzhe Zhang, Yue Huang, Lili Chen, Jin Wang, Xiang-Jiao Yang, and Xiuli Bi**

A

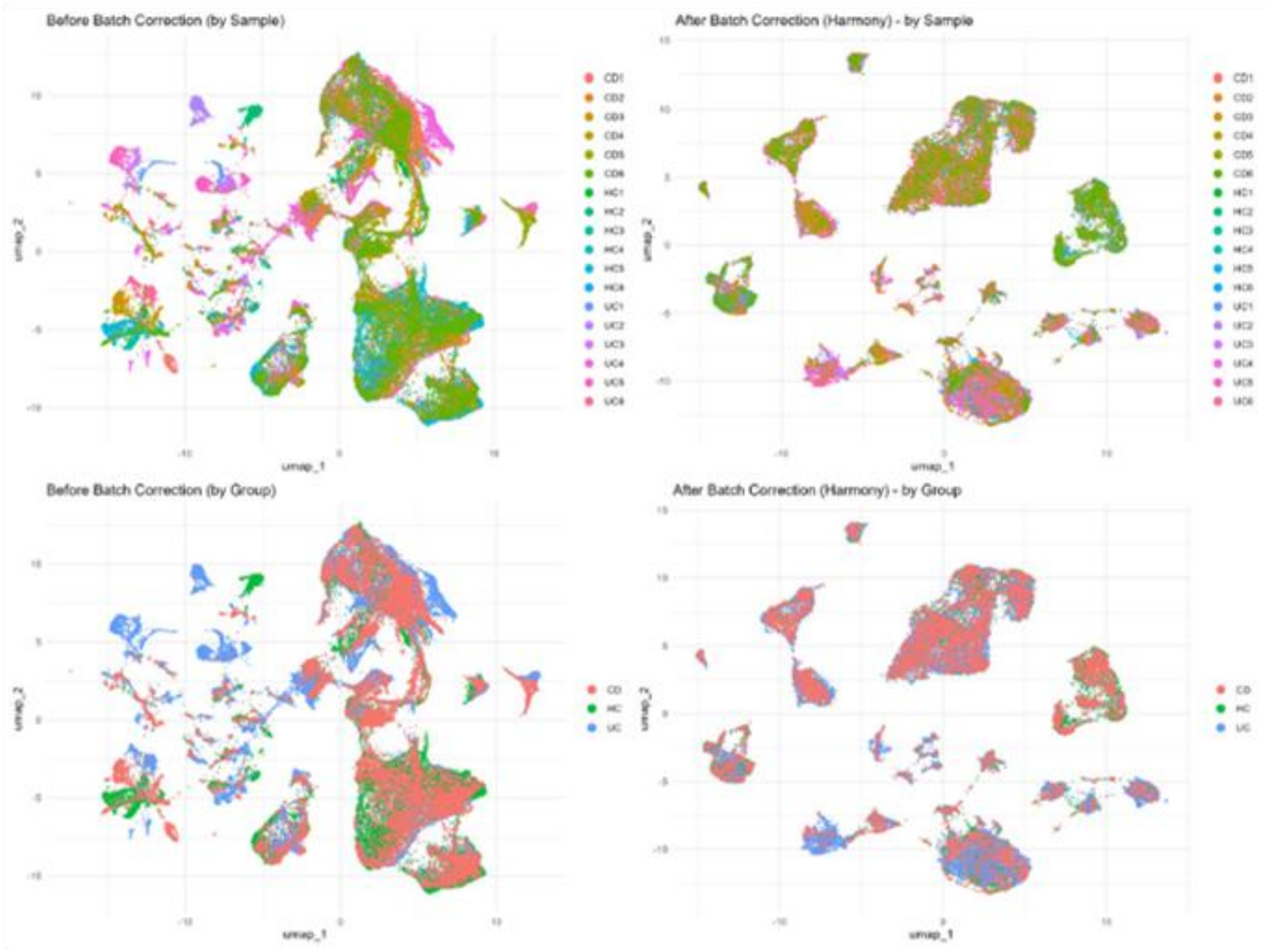


B

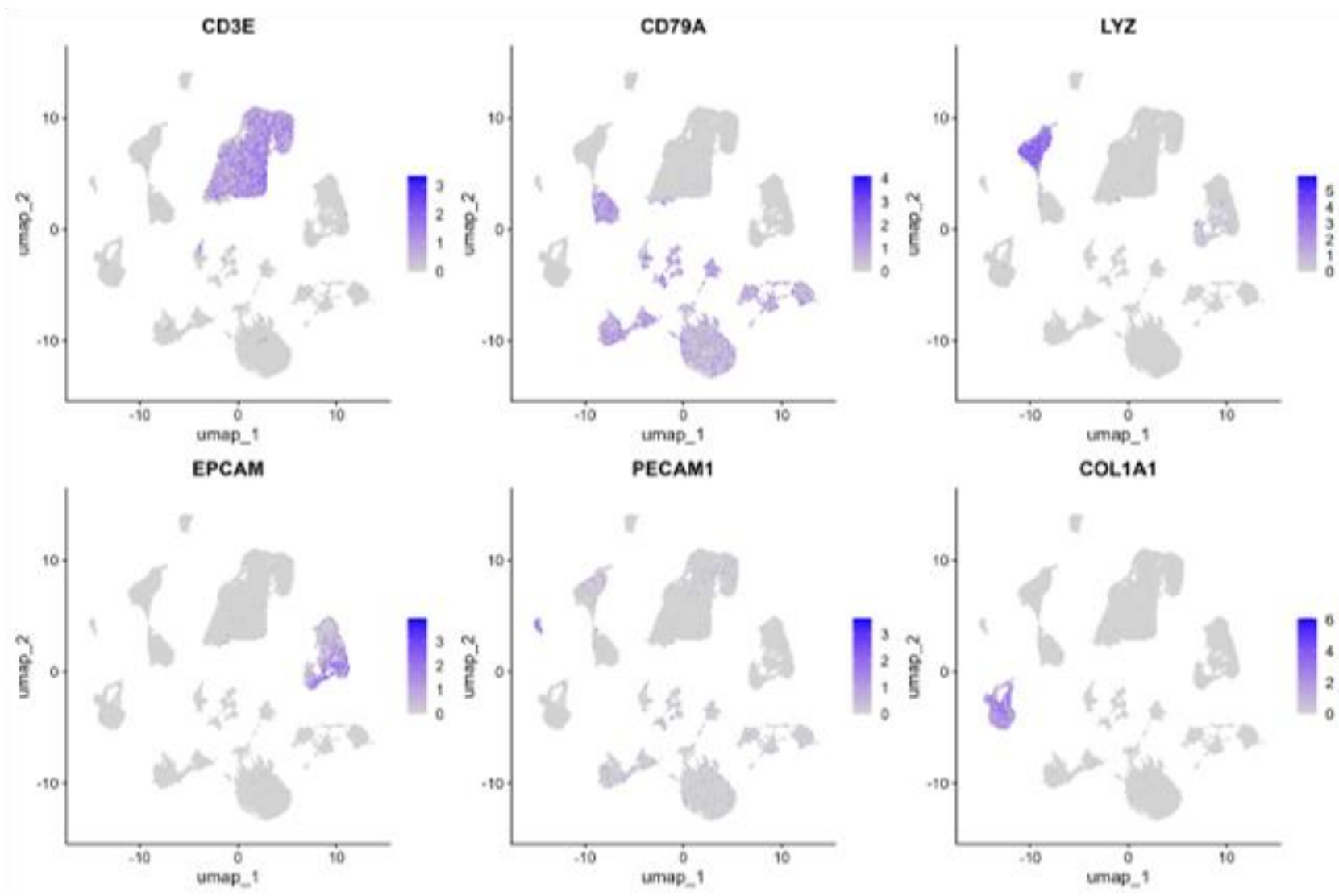


**Figure S1. Clustering and annotation of cells, Related to Figure 1.**

A) Clustering and annotation of cells before and after batch correction. The results show that cells clustered primarily by sample, exhibiting a clear batch effect. Cells were more evenly intermixed after correction, which preserved the biological differences between disease groups. B) Identification of marker genes. The results showed that marker genes were specifically expressed in their corresponding cell clusters, validating the accuracy of cell type annotations.

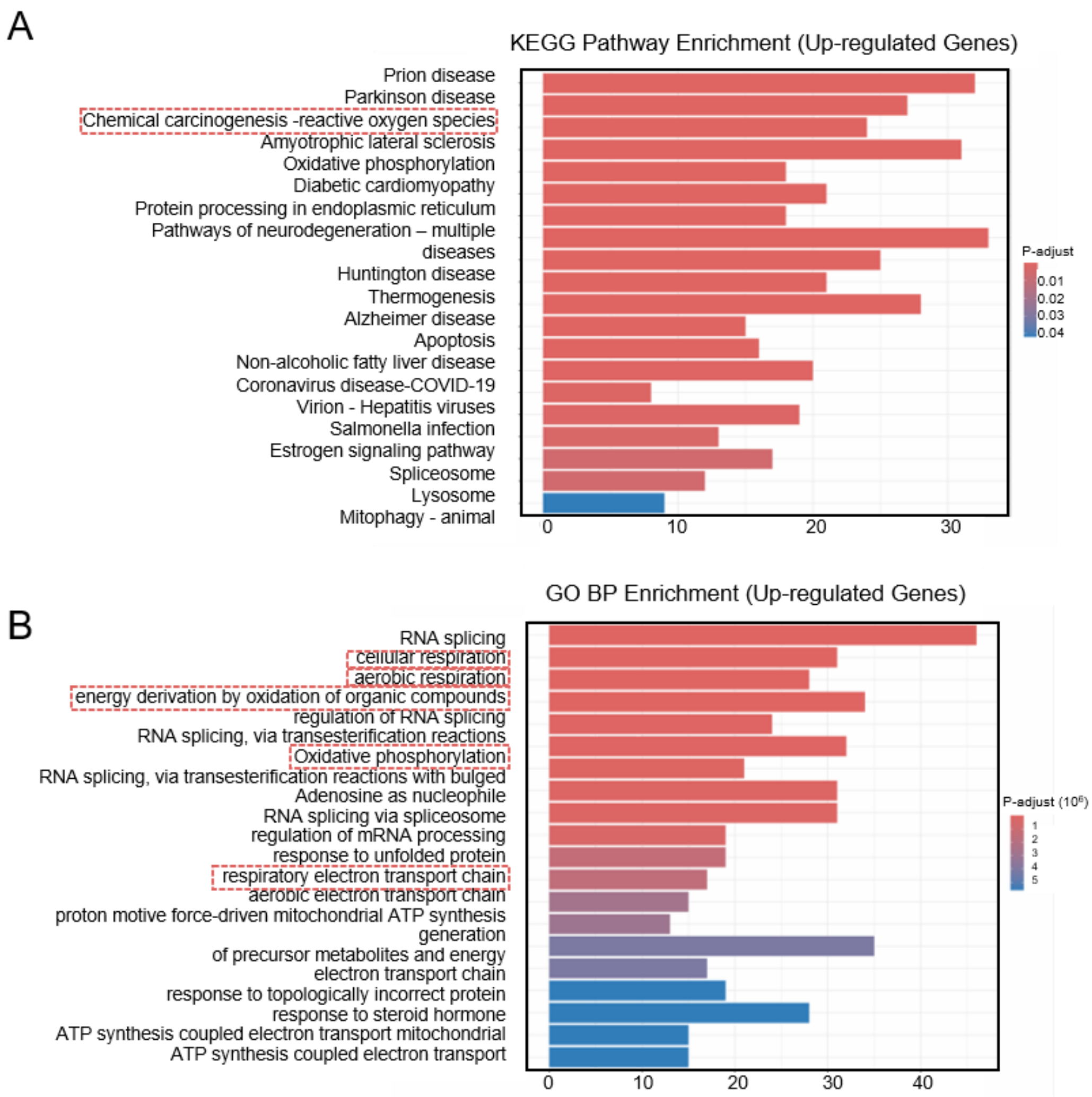


**Figure S2. Differentially expressed genes (DEGs) enriched in oxidative stress pathways, Related to Figure 1.**

(A) KEGG and (B) GO pathway enrichment analyses. Differentially expressed genes (DEGs) enriched in oxidative stress, inflammatory response and ROS metabolism pathways.

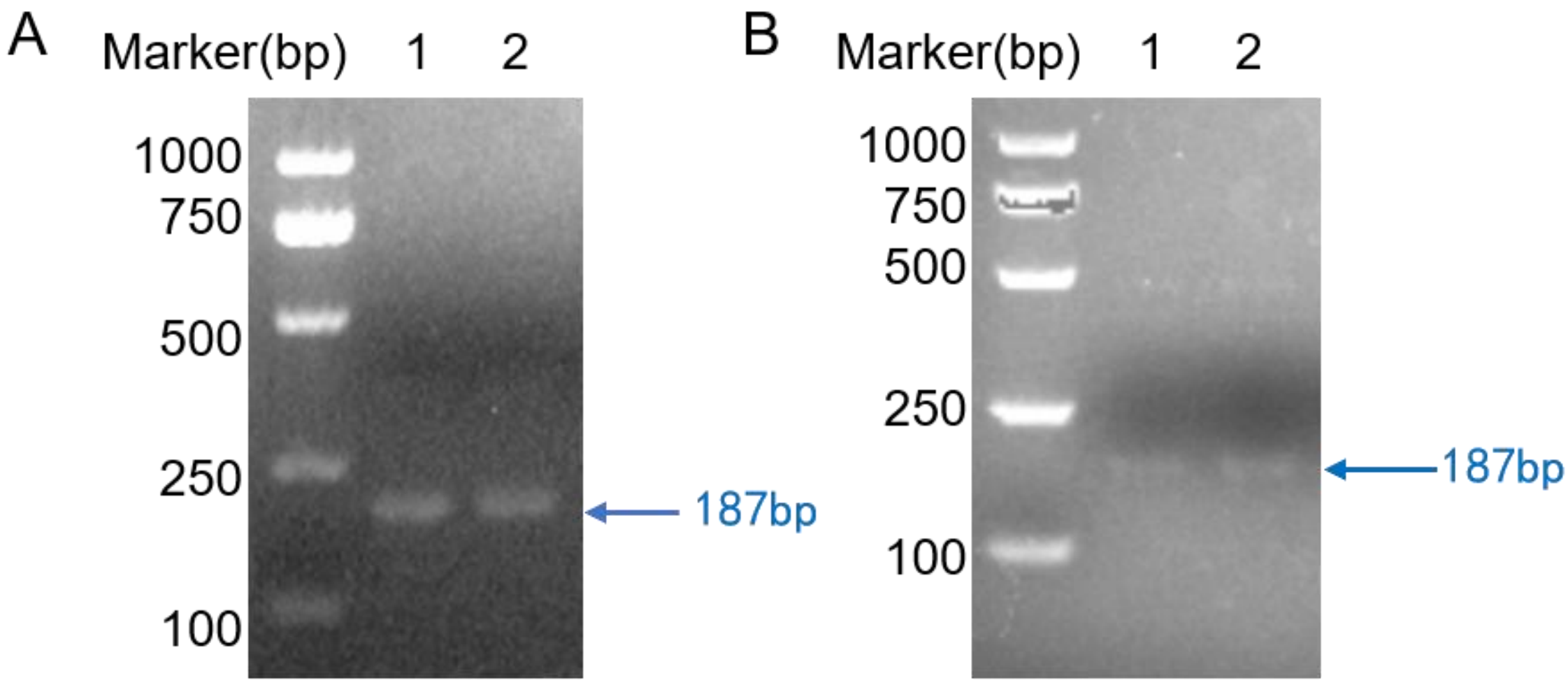


**Figure S3. Verification of the gp91$^{phox}$ gene sequence in cultured cells and colon tissues, Related to Figure 1.**

A) Verification of the gp91$^{phox}$ gene sequence in cultured cells. lane 1, Caco-2 cells, lane 2, RAW264.7 cells. B) Verification of the gp91$^{phox}$ gene sequence from colon tissues, lanes 1 and 2 represent repetitive testing.

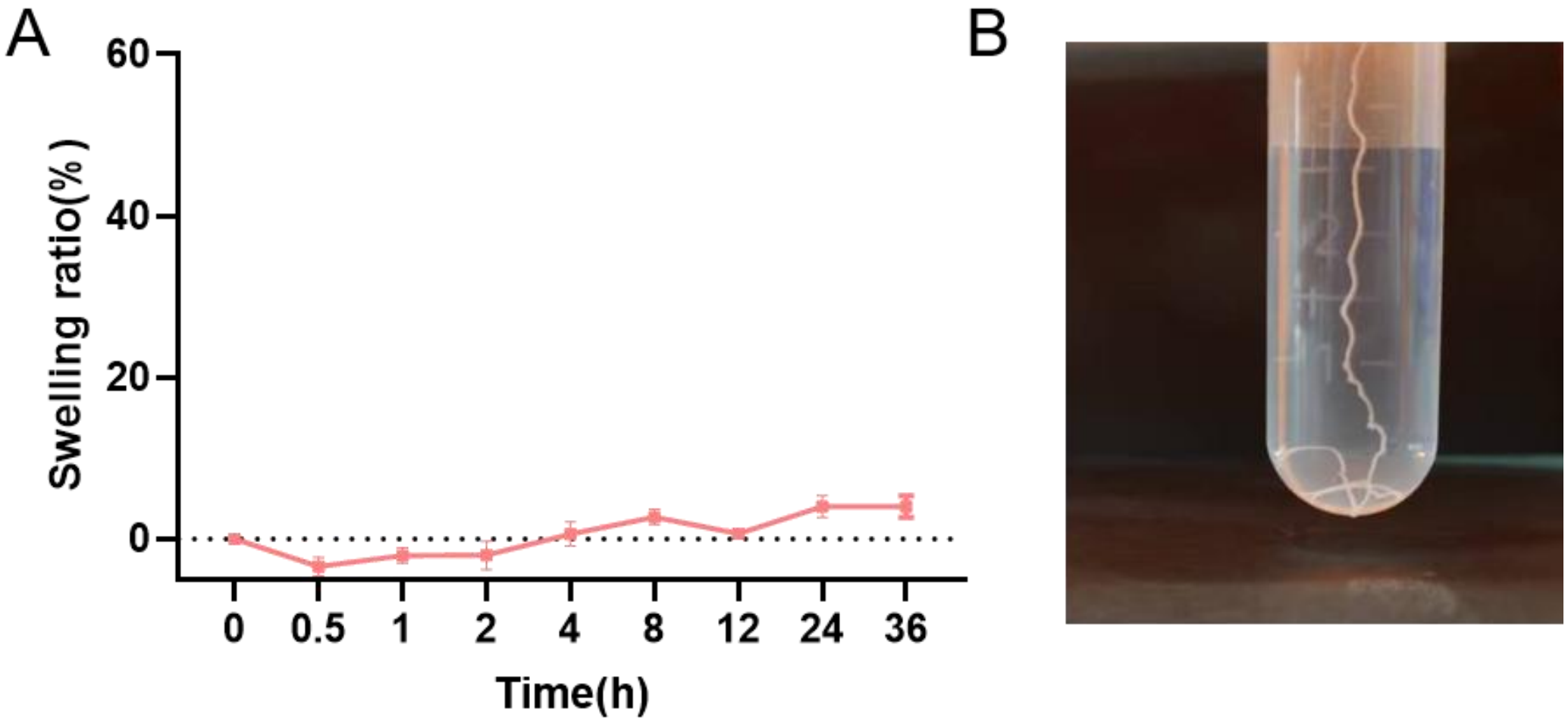


**Figure S4. Swelling ratio and injectability of EcN-RNP@HCMg hydrogel, Related to Figure 1.**

Swelling ratio A) and injectability B) of EcN-RNP@HCMg hydrogel. EcN-RNP@HCMg exhibits a maximum swelling ratio and injectability, these characteristics effectively reduce the risk of intestinal embolism and laceration.

A

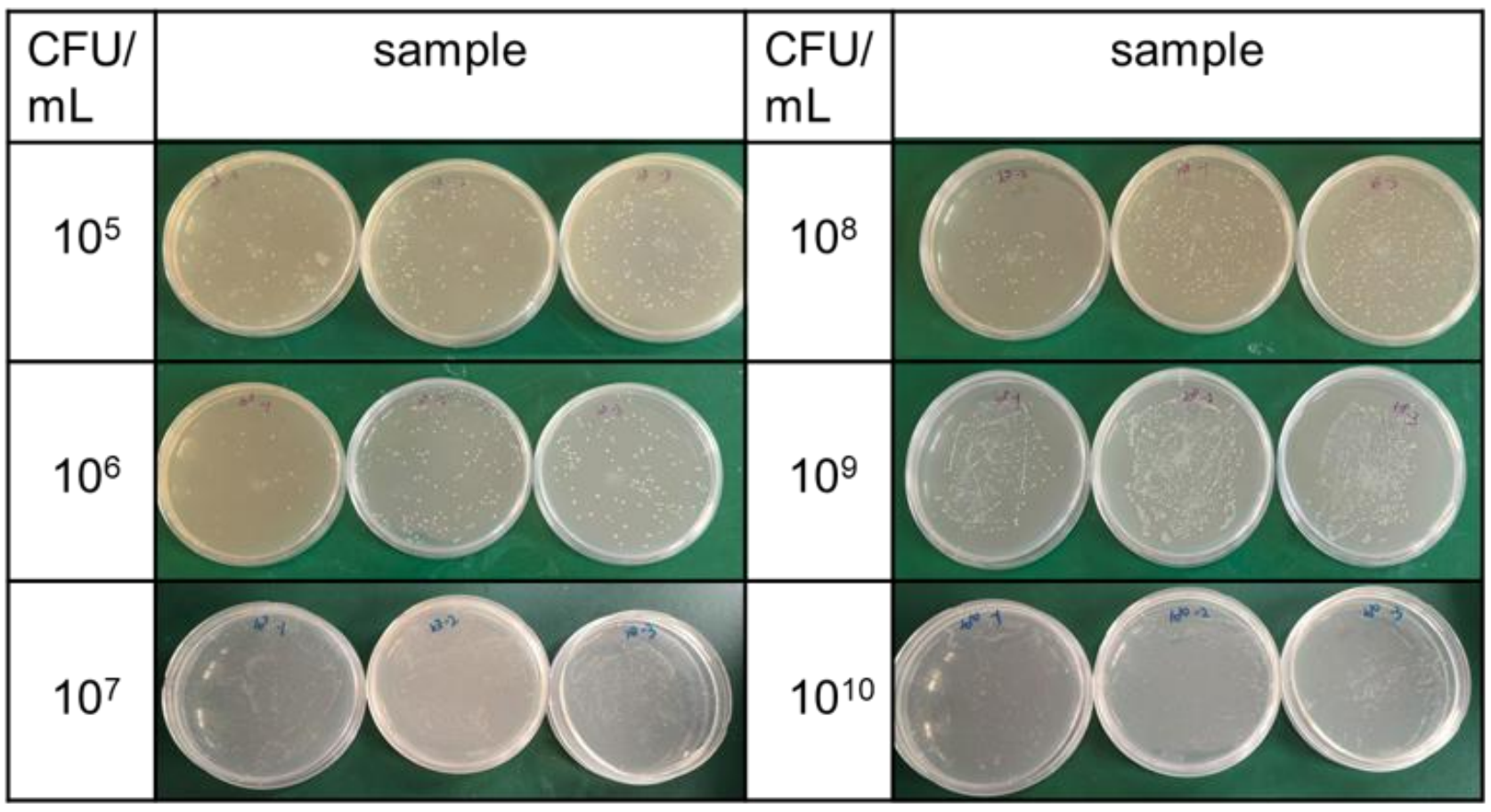


B

| bacteria (CFU/mL) | sample 1 | sample 2 | sample 3 | encapsulation rate% |
|---|---|---|---|---|
| $10^5$ | 78 | 95 | 140 | 1.04% |
| $10^6$ | 11 | 176 | 119 | 0.92% |
| $10^7$ | 110 | 100 | 90 | 0.93% |
| $10^8$ | 59 | 295 | 423 | 2.59% |
| $10^9$ | 1100 | 800 | 750 | 8.83% |
| $10^{10}$ | 90 | 60 | 50 | 0.67% |

**Figure S5. Encapsulation analysis of EcN-RNP@HCMg hydrogel, Related to Figure 1.**

A) Images of colonies showing bacterial encapsulation analysis. B) Encapsulation efficiency statistics. The results show that the maximum encapsulation efficiency of 8.83% by HA, CS and $MgCl_2$ was achieved at a bacterial concentration of $10^9$ CFU/mL.

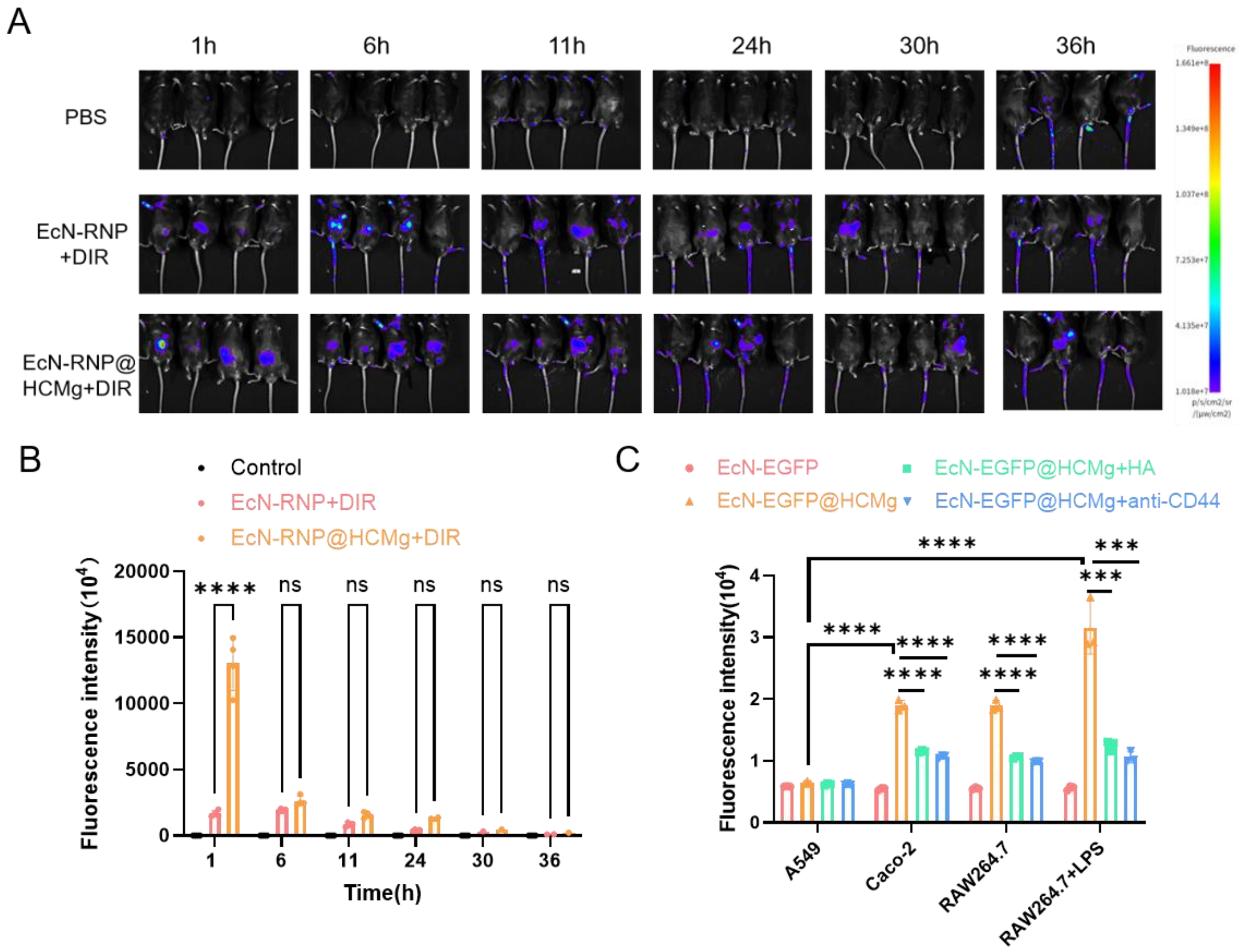


**Figure S6. IVIS and cell uptake analysis of HA Targeting Properties, Related to Figure 2.**

Fluorescence images of IBD mice following oral administration (A and B). C) Statistics of the uptake of EcN-EGFP@HCMg by different cells. Data are presented as mean ± SD, ns: no significance, *p < 0.05, **p < 0.01 and ***p < 0.001 calculated using Student’s test or one-way ANOVA.

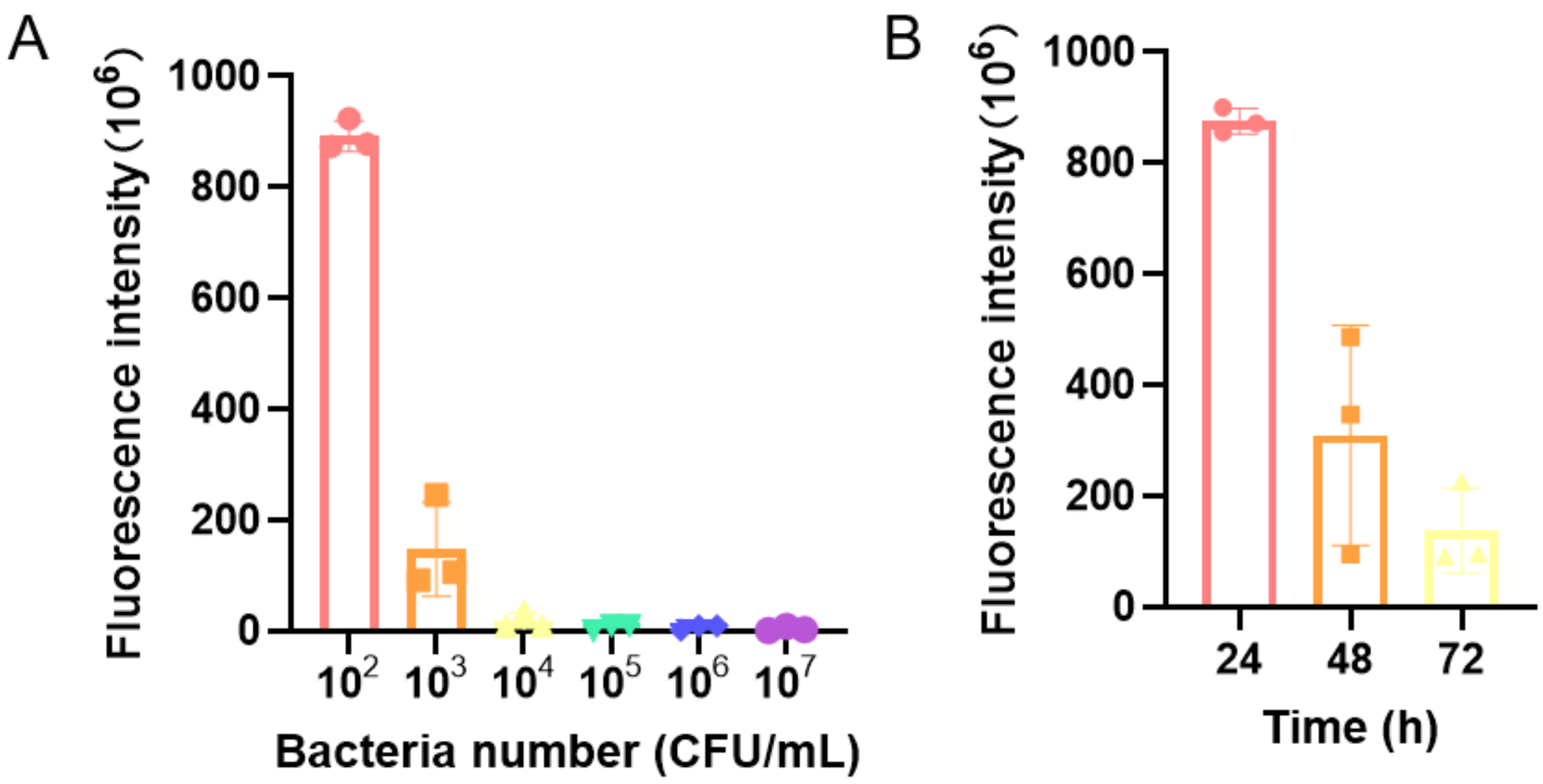


**Figure S7. Intracellular ROS levels Caco-2 cells, Related to Figure 3.**

Intracellular ROS levels Caco-2 cells were treated by EcN-RNP@HCMg of different EcN concentrations (A) and different times (B). Cells were co-cultured with EcN-RNP@HCMg at concentrations of $10^2$, $10^3$, $10^4$, $10^5$, $10^6$, and $10^7$ CFU/mL, and the results showed that higher bacterial concentrations led to lower ROS levels. Moreover, the effects of EcN-RNP@HCMg on reducing ROS levels gradually enhanced as the co-culture duration increased. Data are presented as mean ± SD, ns: no significance, $*p < 0.05$, $**p < 0.01$ and $***p < 0.001$ calculated using Student's test or one-way ANOVA.

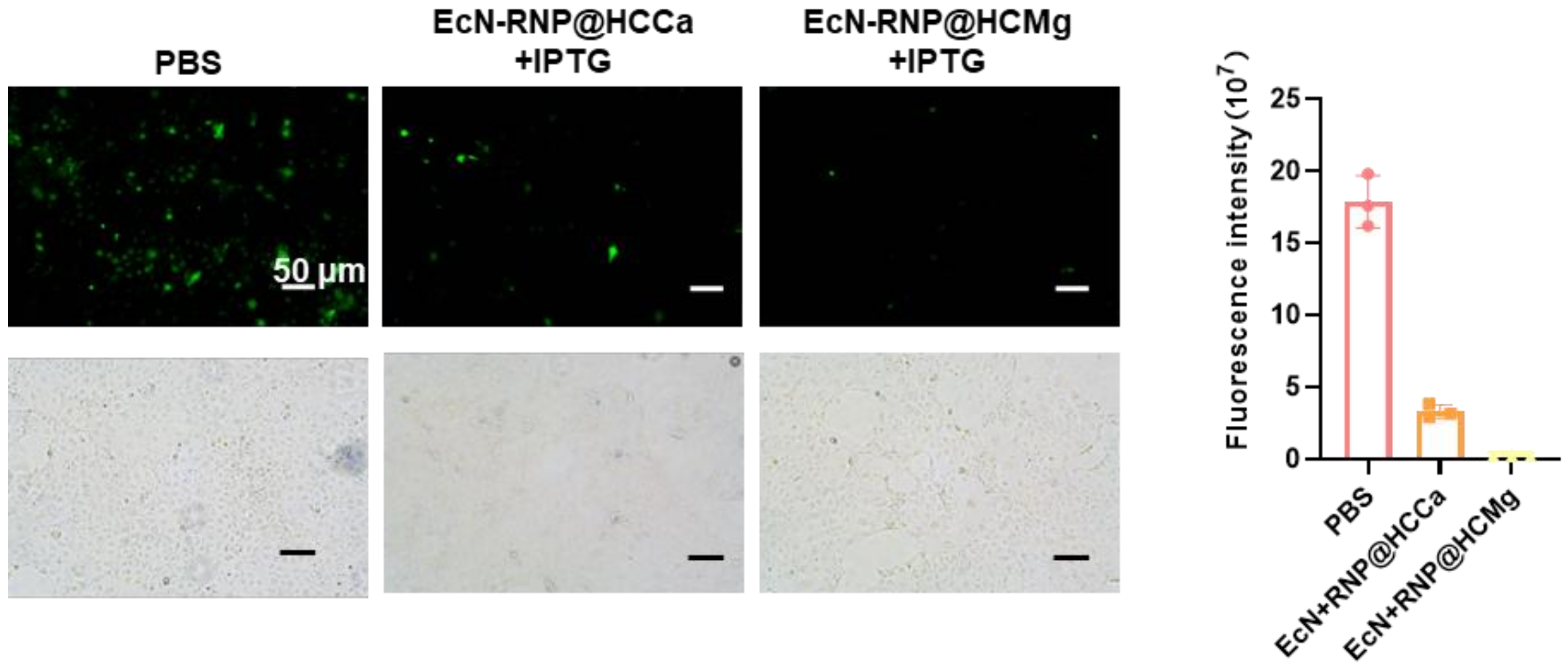


**Figure S8. Effects of $Mg^{2+}$ and $Ca^{2+}$ were compared by detecting the intracellular ROS levels, Related to Figure 3.**

Effects of EcN-RNP@HCCa+IPTG and EcN-RNP@HCMg+IPTG on reducing ROS levels in Caco-2 cells following $H_2O_2$ treatment. The results show that both EcN-RNP@HCCa+IPTG and EcN-RNP@HCMg+IPTG can reduce ROS levels in cells, with EcN-RNP@HCMg+IPTG exhibiting a superior effect. Data are presented as mean ± SD, ns: no significance, *p < 0.05, **p < 0.01 and ***p < 0.001 calculated using Student's test or one-way ANOVA.

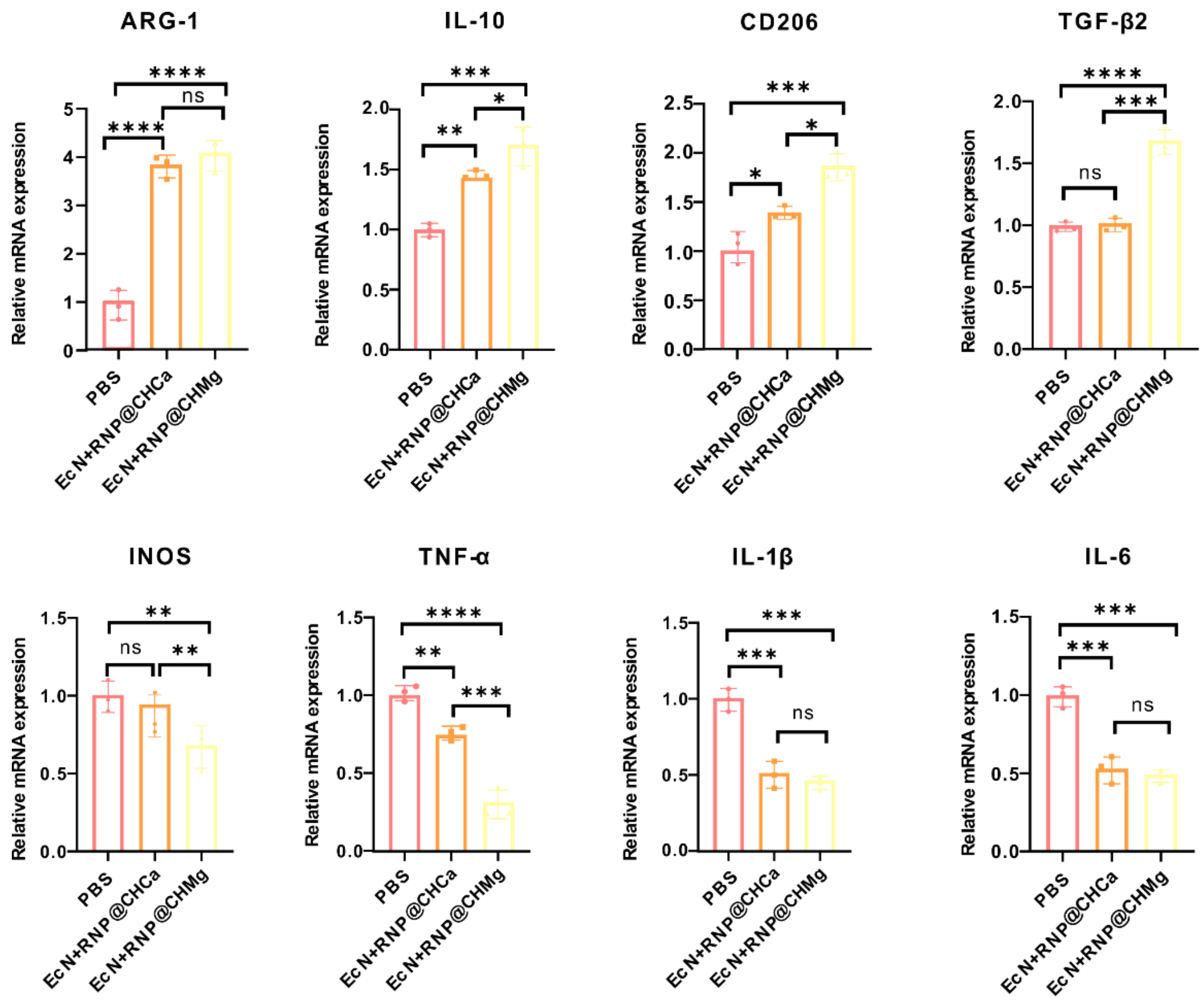


**Figure S9. Effects of $Mg^{2+}$ and $Ca^{2+}$ were compared by detecting the expression of inflammatory factors, Related to Figure 3.**

Effects of EcN-RNP@HCCa + IPTG and EcN-RNP@HCMg + IPTG on mRNA expression of inflammatory cytokines in LPS-treated RAW264.7 cells. Data are presented as mean ± SD, ns: no significance, *p < 0.05, **p < 0.01 and ***p < 0.001 calculated using Student's test or one-way ANOVA.

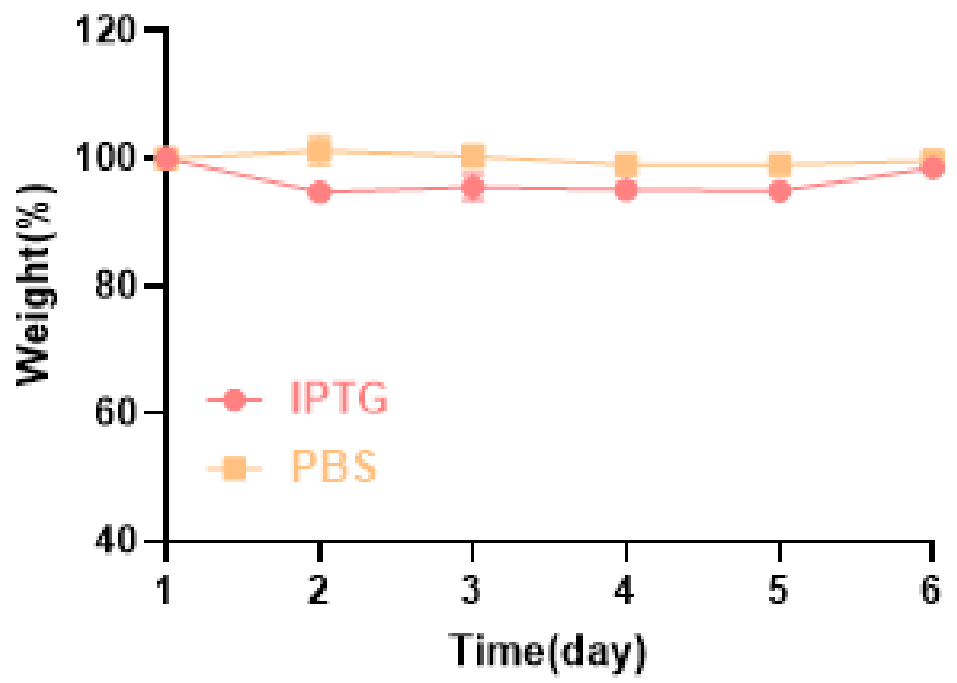


**Figure S10. In vivo safety testing of IPTG, Related to Figure 4.**

Body weight variation in mice after oral administration of IPTG. The body weight of mice over six days showed no significant difference in IPTG and PBS groups, demonstrating the safety of IPTG.

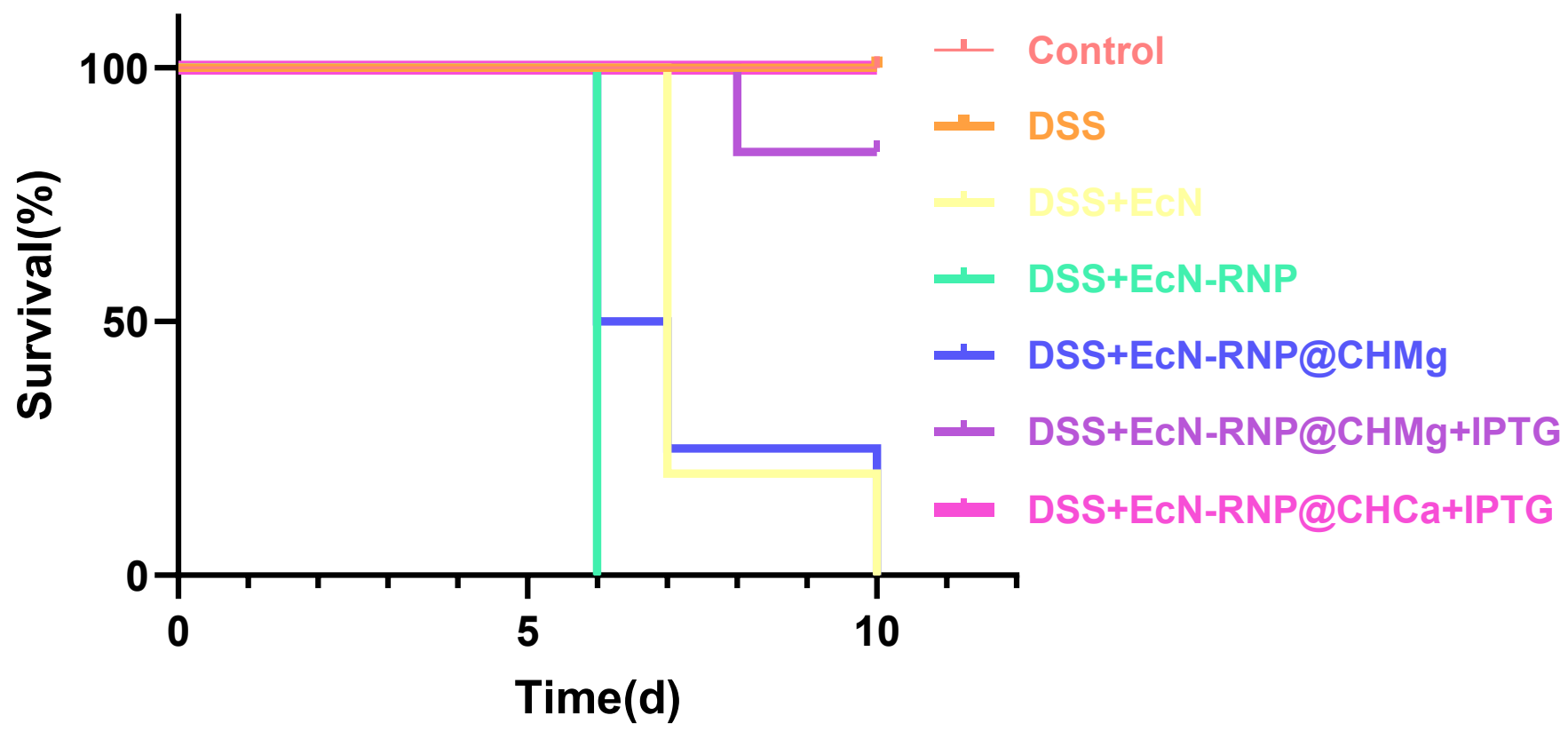


**Figure S11. Survival curves, Related to Figure 4.**

The results show that mice in groups except control, EcN-RNP@HCCa + IPTG and EcN-RNP@HCMg + IPTG died progressively.

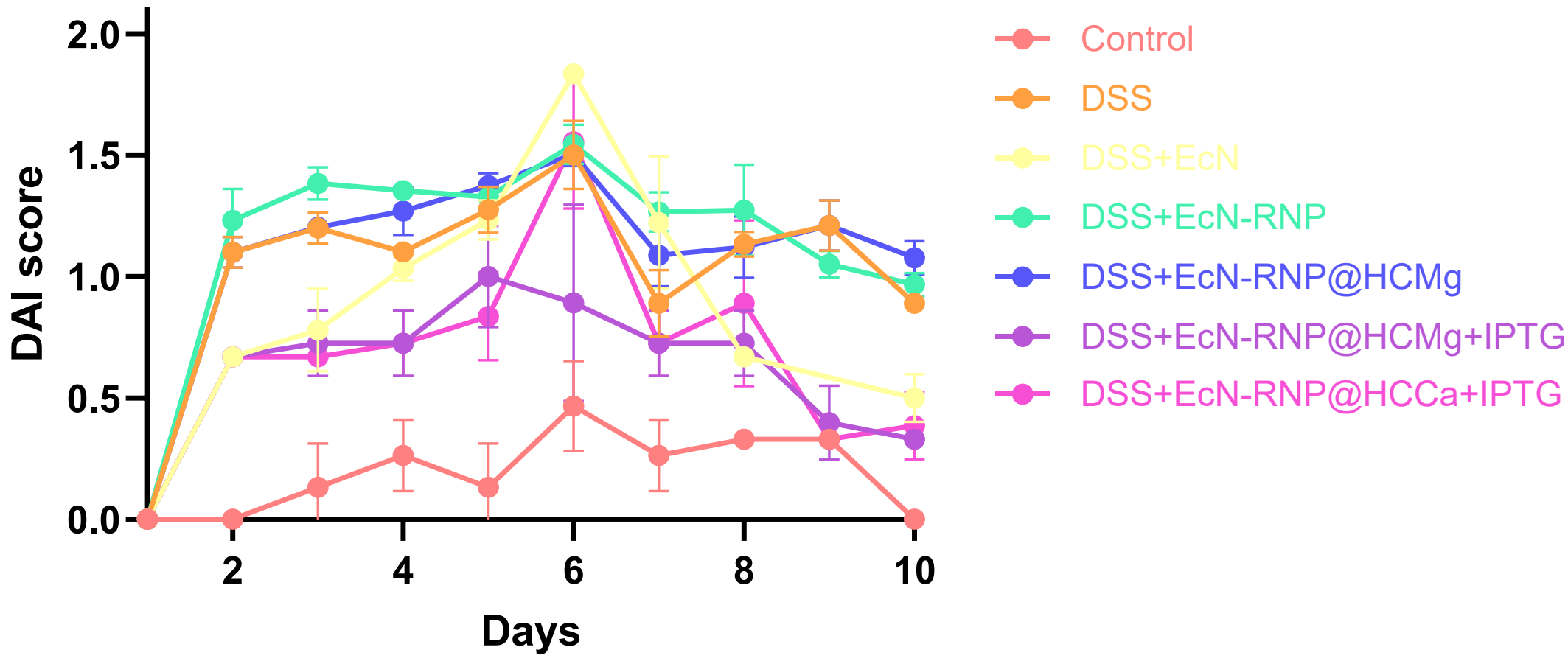


**Figure S12. DAI scores variation curve, Related to Figure 4.**

The DAI scores variation curve during the 10-day monitoring period. The DAI scores of mice showed a consistent increase in all groups after DSS treatment. However, these scores reduced in mice with oral administration of EcN-RNP@HCMg + IPTG, leading to a gradual normalization towards control group levels.

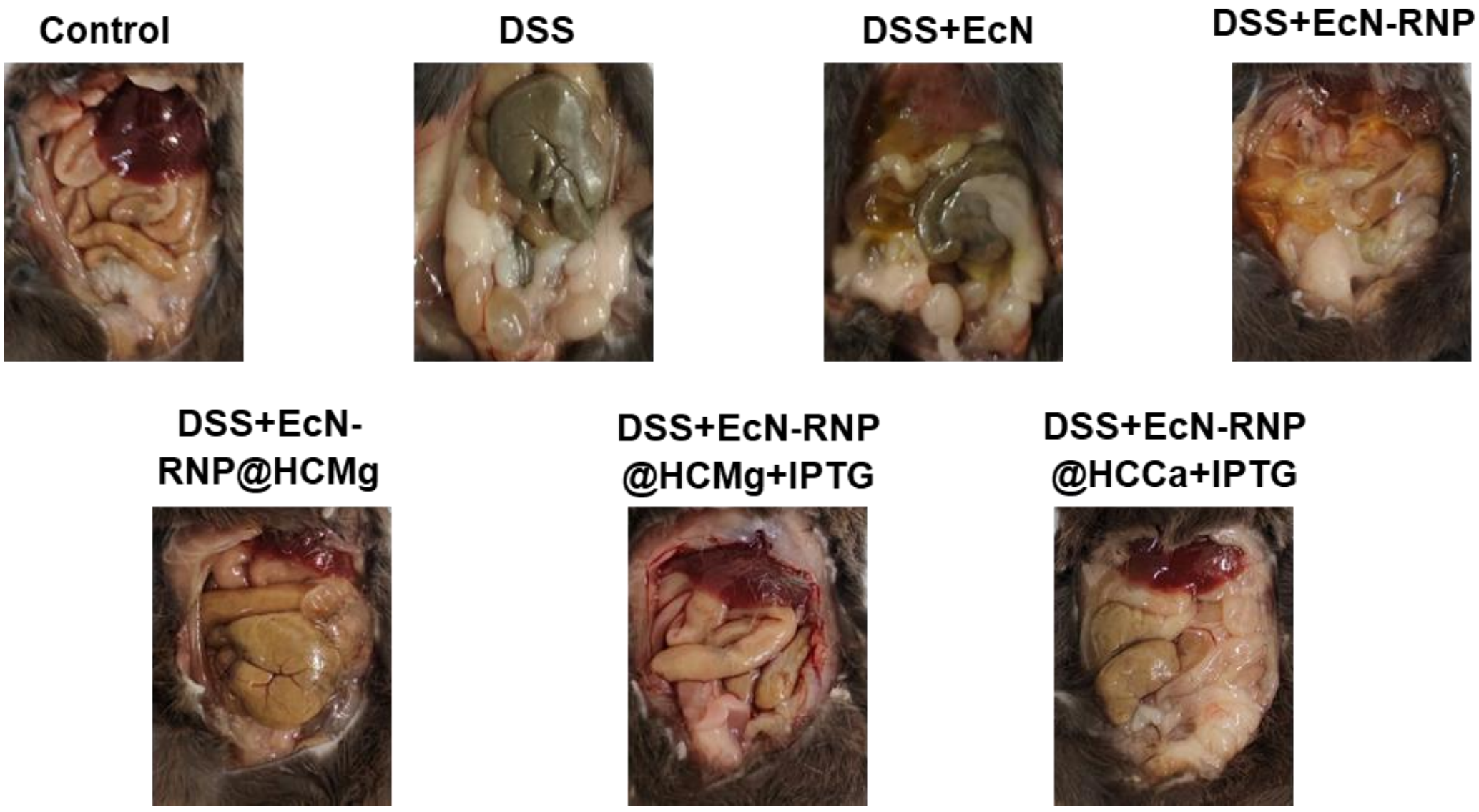


**Figure S13. Macroscopic pictures of abdominal condition, Related to Figure 4.**

Macroscopic pictures of abdominal condition from each group. Colons in mice exhibited tissue adhesions and irregular morphology after DSS treatment, whereas these conditions were alleviated in mice treated with EcN-RNP@HCMg + IPTG.

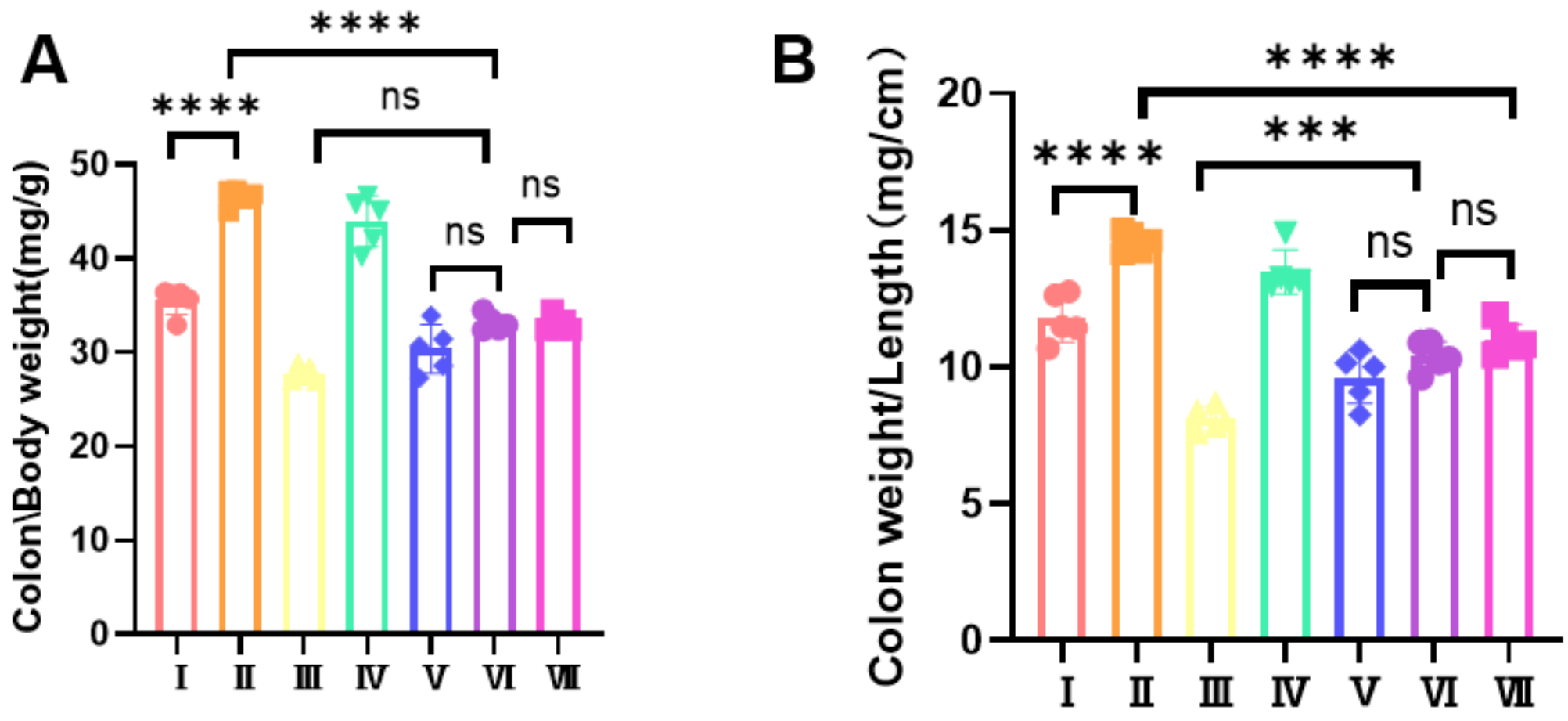


**Figure S14. Colon weight and length characteristics, Related to Figure 4.**

Colon weight and length characteristics. A) Colon /body weight ratio. B) Colon weight/length ratio. I: PBS, II: DSS, III:DSS+EcN, IV:DSS+EcN-RNP, V: DSS+EcN-RNP@HCMg, VI: DSS+EcN-RNP@ HCMg+IPTG, VII: DSS+EcN-RNP@HCCa+IPTG. The colon/body weight and colon weight/length ratio of mice significantly increased following DSS treatment, while these indices of mice treated with EcN-RNP@HCMg+IPTG reduced. Data are presented as mean ± SD, ns: no significance, *$p < 0.05$, **$p < 0.01$ and ***$p < 0.001$ calculated using Student's test or one-way ANOVA.

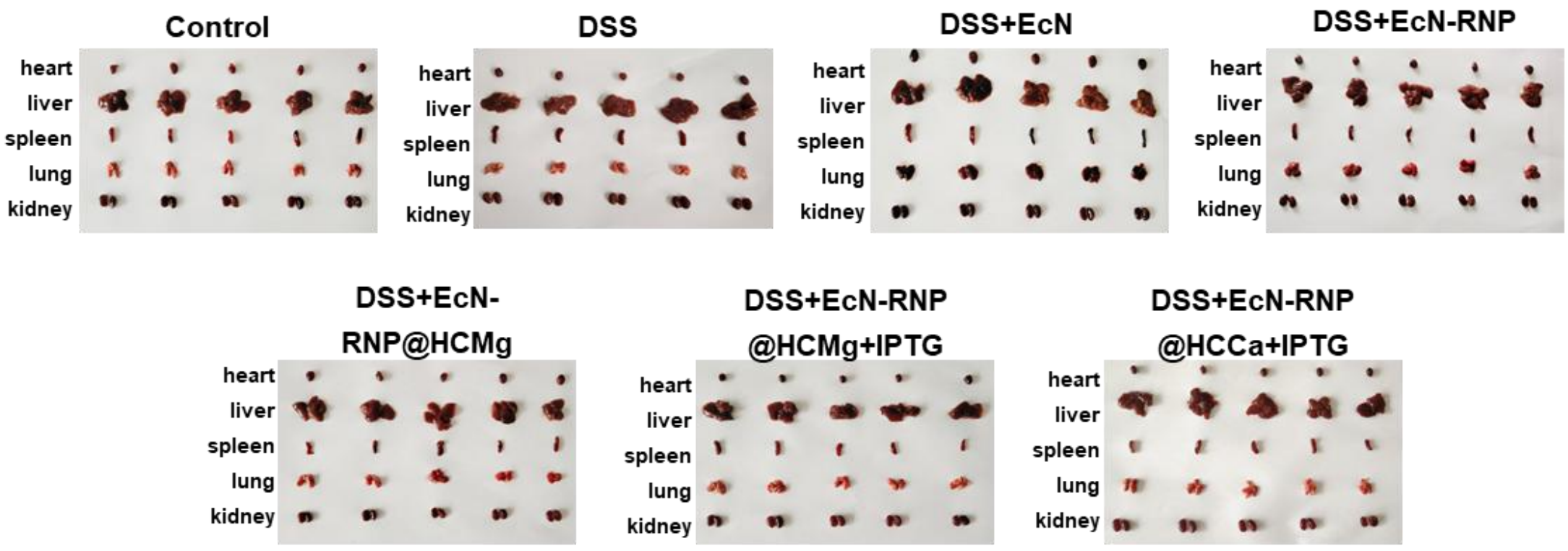


**Figure S15. Major organ pictures, Related to Figure 4.**

Macroscopic pictures of heart, liver, spleen, lung and kidney (another organ as there are five arrays from each group.

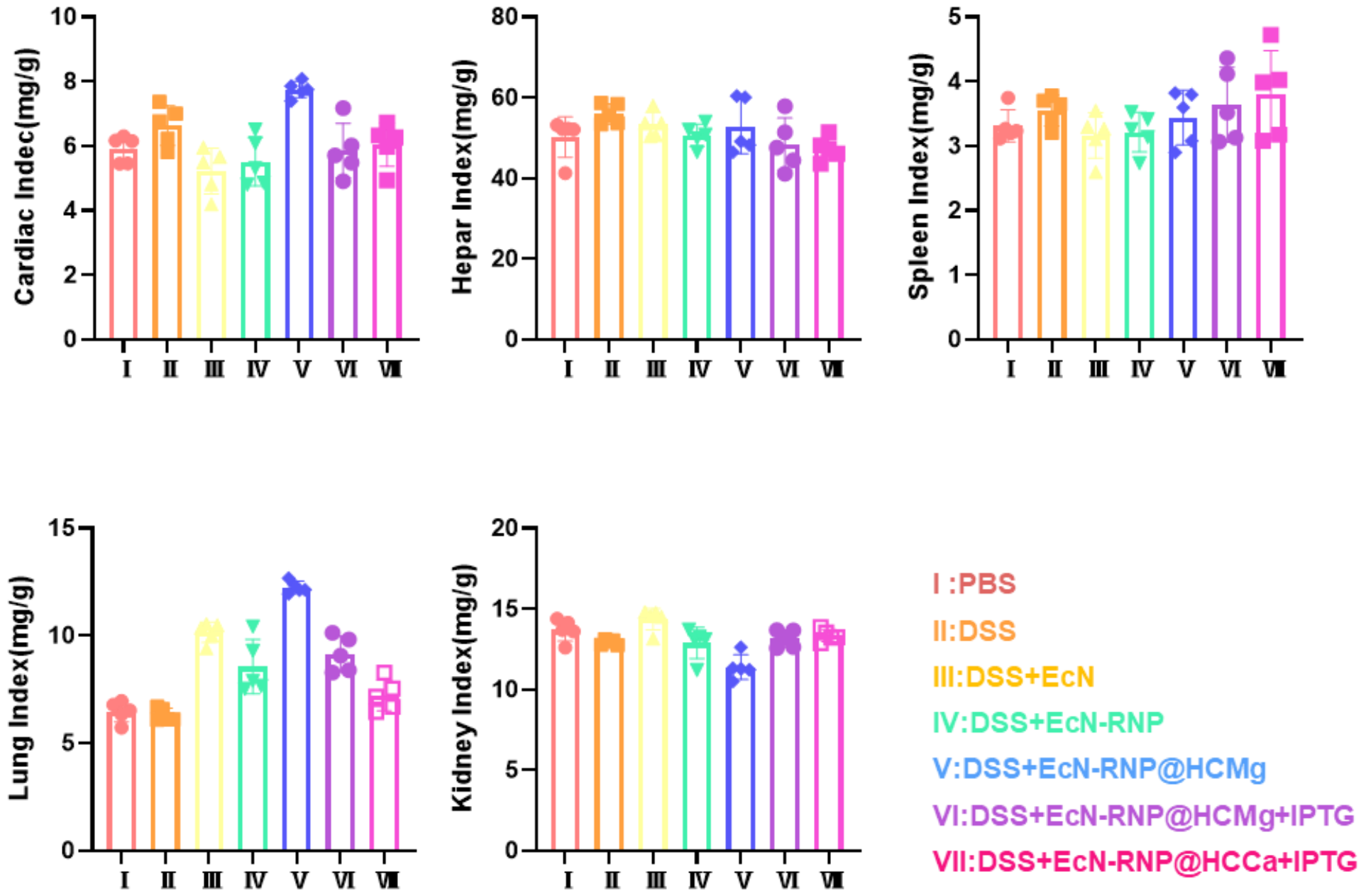


**Figure S16. Organ indices of heart, liver, spleen, lung, and kidney, Related to Figure 4.** The results show no significant differences in organ indices among groups, indicating that samples did not adversely affect mice organs. I: PBS, II: DSS, III: DSS+EcN, IV: DSS+EcN-RNP, V: DSS+EcN-RNP@HCMg, VI: DSS+EcN-RNP@HCMg+IPTG, VII: DSS+EcN-RNP@HCCa+IPTG.

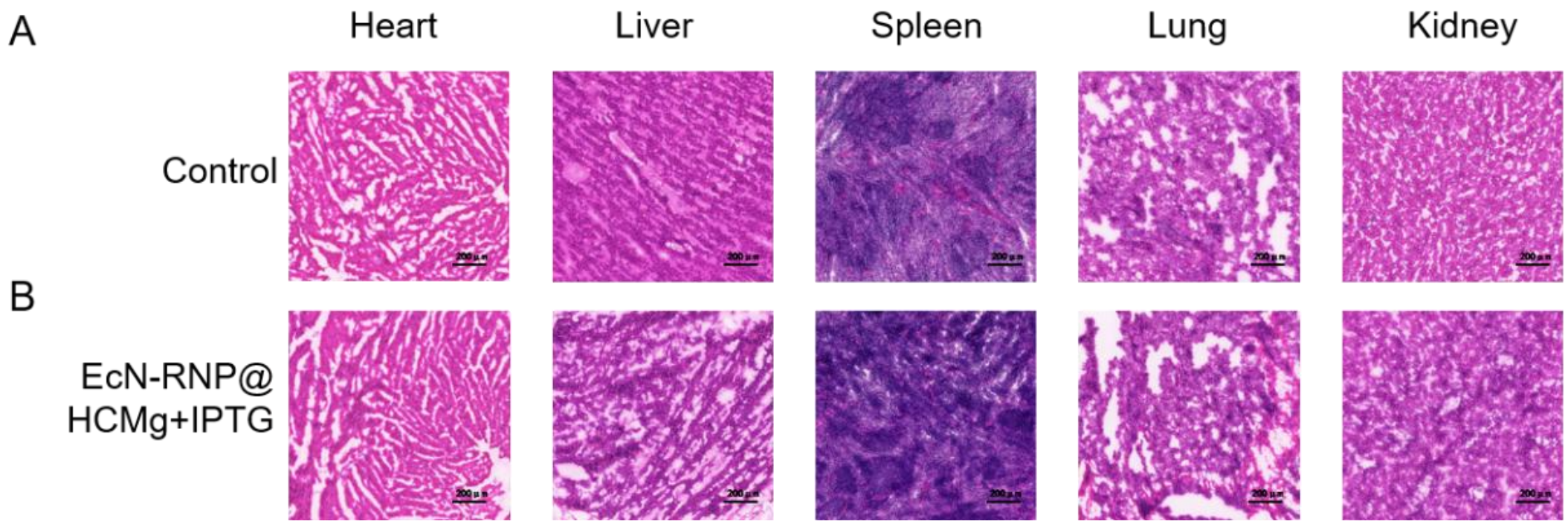


**Figure S17. H&E staining of major organs, Related to Figure 4.**
H&E staining of the heart, liver, spleen, lung and kidney tissues in mice from the control (A) and EcN-RNP@HCMg+IPTG (B) groups.

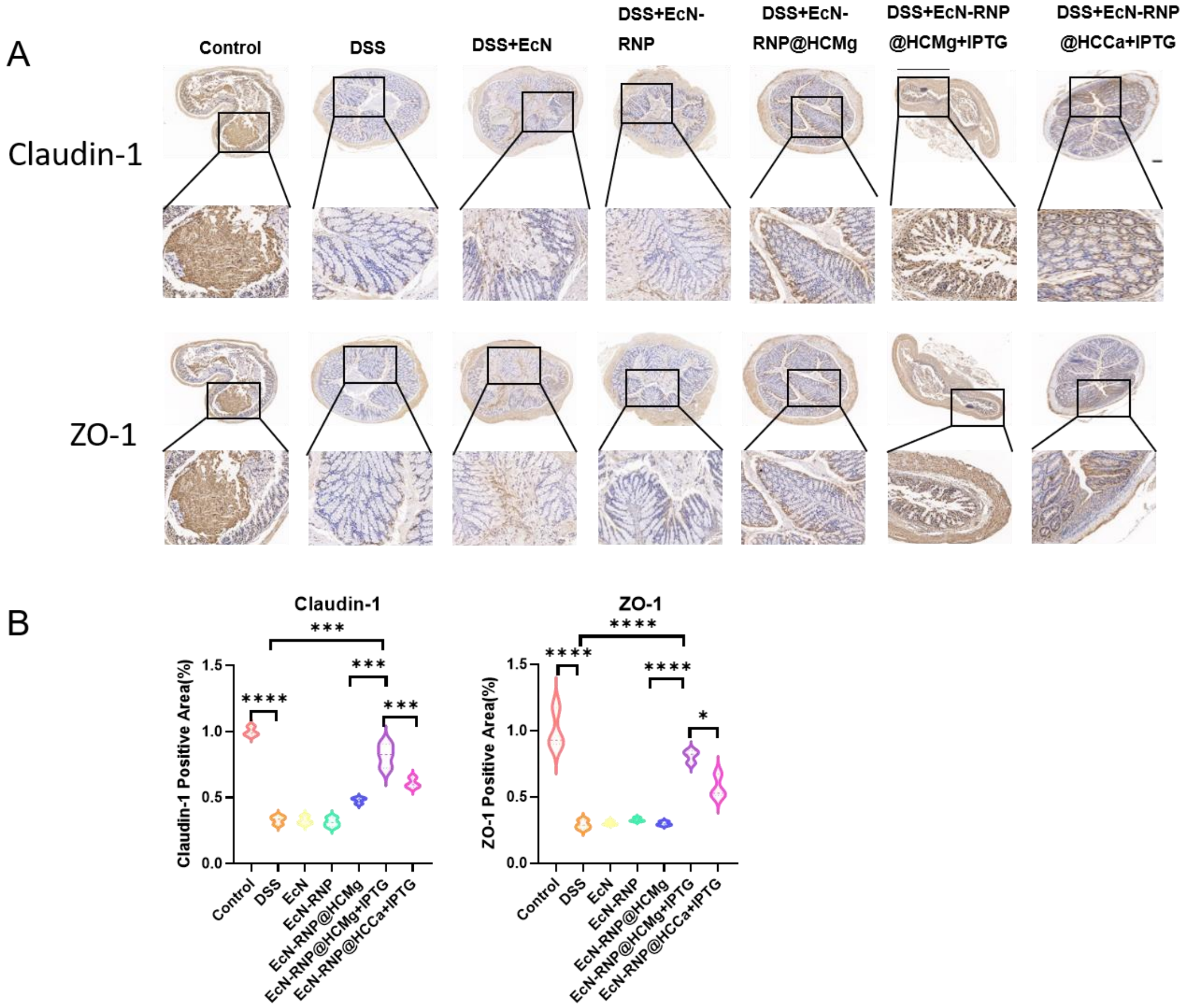


**Figure S18. Immunohistochemical staining of Claudin-1 and ZO-1, Related to Figure 4.**

Immunohistochemical staining images A) and statistical analysis B) of Claudin-1 and ZO-1 in colon tissues. The strong brown-stained intensity of Claudin-1 and ZO-1 exhibited in DSS + EcN-RNP@HCMg + IPTG group, indicating its capacity to repair the intestinal epithelial barrier. Data are presented as mean ± SD, ns: no significance, *p < 0.05, **p < 0.01 and ***p < 0.001 calculated using Student's test or one-way ANOVA.

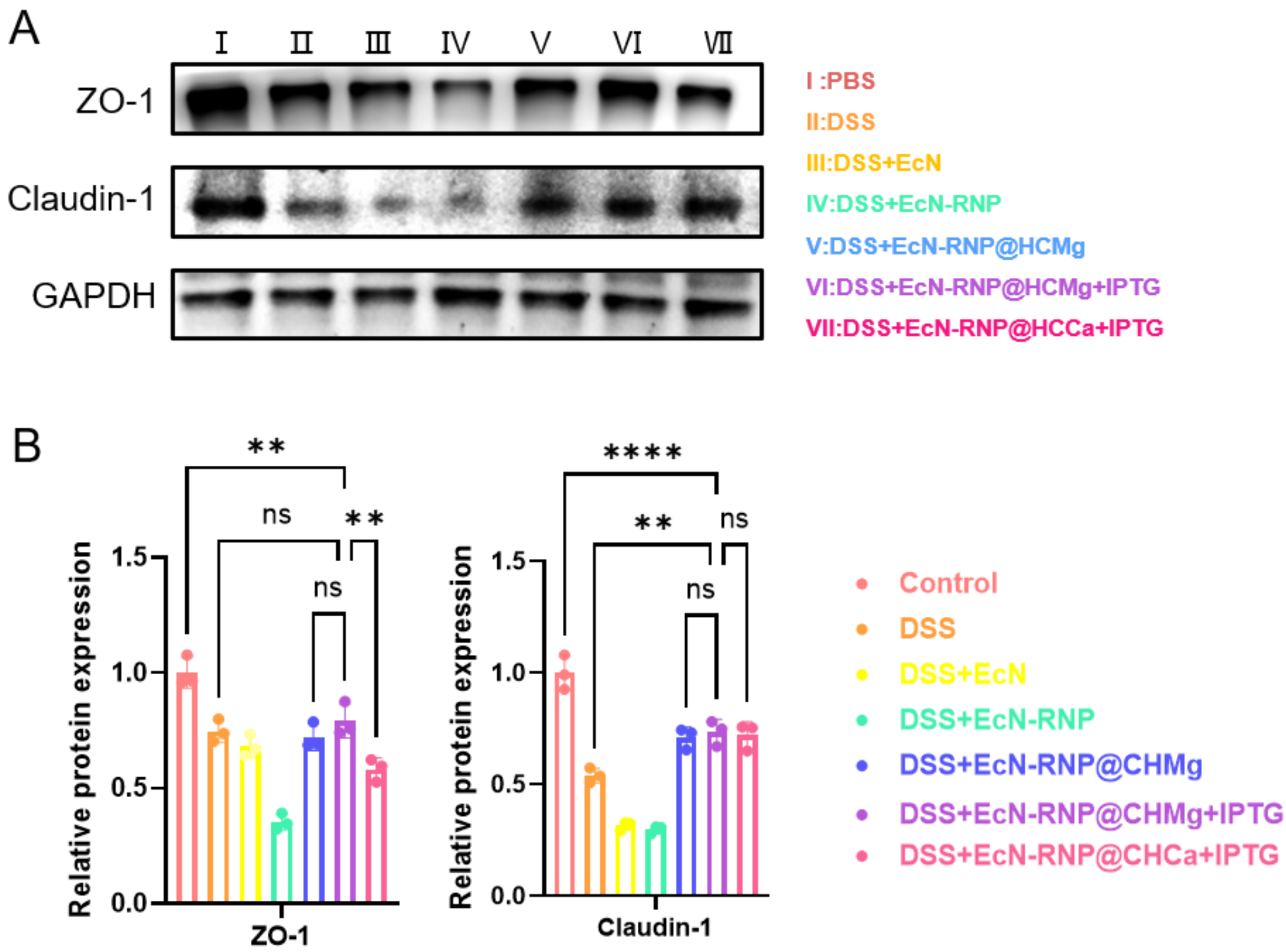


**Figure S19. Detection of intestinal epithelial barrier permeability, Related to Figure 4.**

Detection of intestinal epithelial barrier permeability. A) Expression levels and B) statistical analysis of ZO-1 and Claudin-1 in cells from colon tissues. Data are presented as mean ± SD, ns: no significance, *p < 0.05, **p < 0.01 and ***p < 0.001 calculated using Student's test or one-way ANOVA.

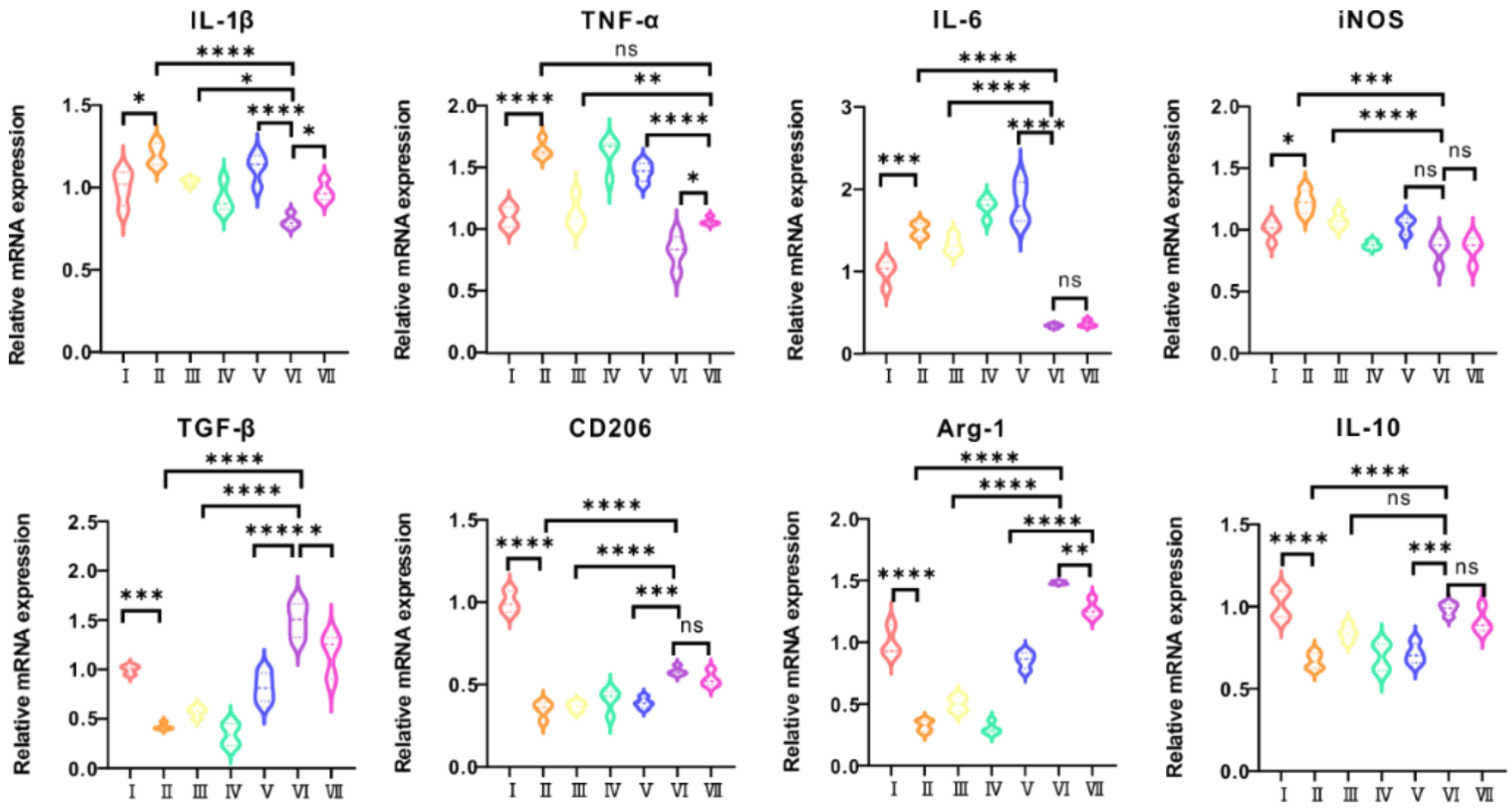


**Figure S20. The mRNA expressions analysis of inflammatory cytokine in colon tissues, Related to Figure 4.**

The mRNA expressions analysis of inflammatory cytokine in colon tissues. I: PBS, II: DSS, III:DSS+EcN, IV:DSS+EcN-RNP, V: DSS+EcN-RNP@HCMg, VI: DSS+EcN-RNP@ HCMg+IPTG, VII: DSS+EcN-RNP@HCCa+IPTG. Data are presented as mean ± SD, ns: no significance, *$p < 0.05$, **$p < 0.01$ and ***$p < 0.001$ calculated using Student's test or one-way ANOVA.

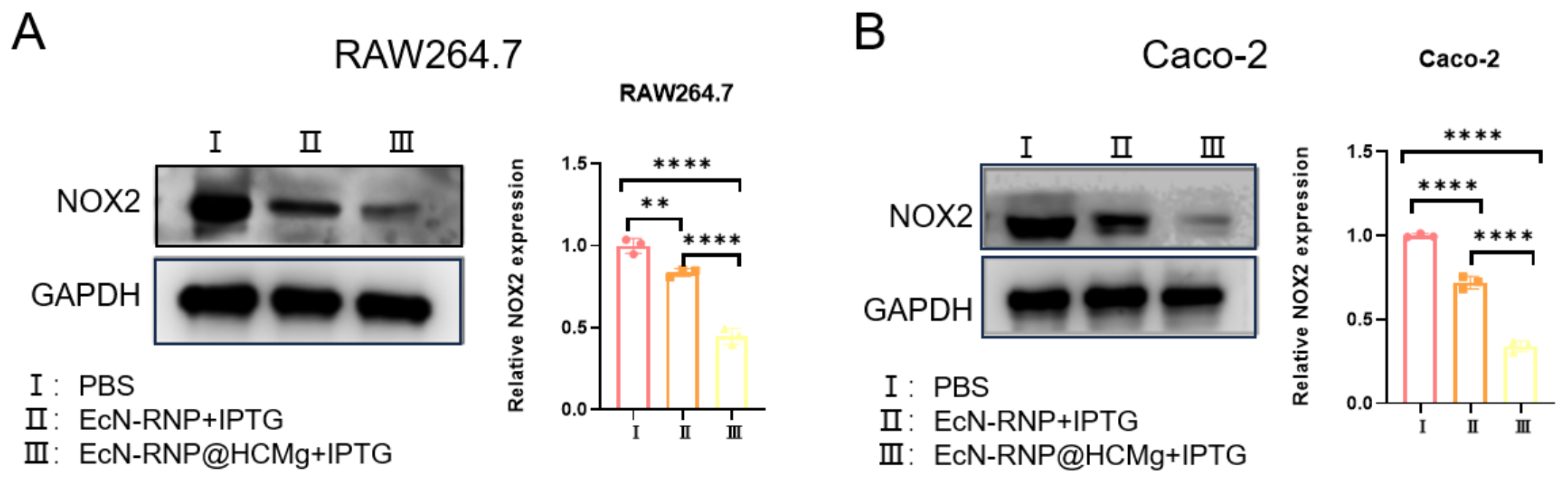


**Figure S21. IPTG induces CRISPR/Cas9 to inhibit the expression of NOX2, Related to Figure 5.**

Expression analysis of NOX2 in RAW264.7 cells A) and Caco-2 cells B) treated by EcN-RNP+IPTG and EcN-RNP@HCMg+IPTG. Data are presented as mean ± SD, ns: no significance, *p < 0.05, **p < 0.01 and ***p < 0.001 calculated using Student's test or one-way ANOVA.

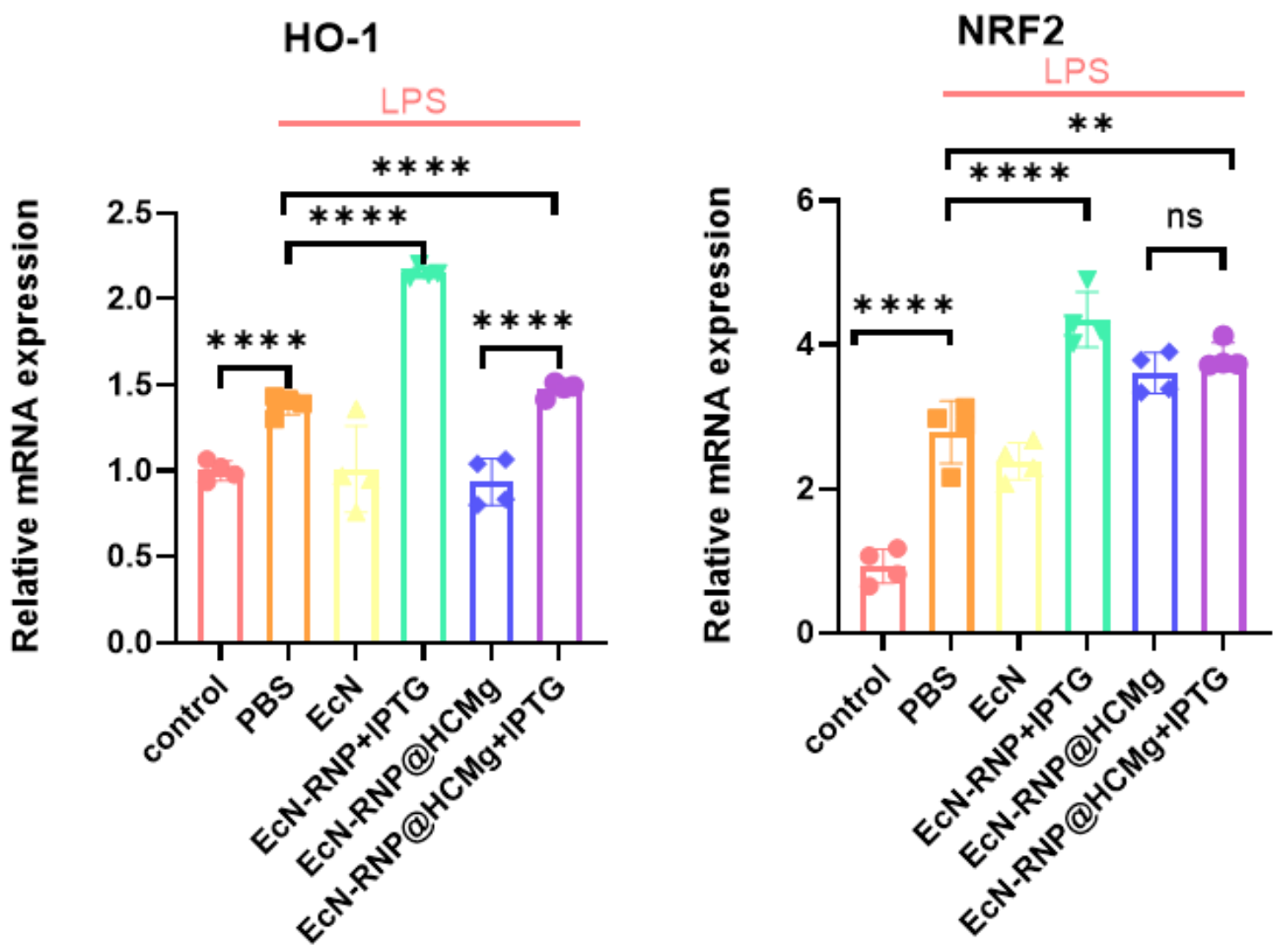


**Figure S22. The mRNA expression levels of HO-1 and NRF2, Related to Figure 5.**

mRNA expression levels of HO-1 and NRF2 in RAW 264.7 cells. The mRNA expression of HO-1 and NRF2 were upregulated in cells treated by EcN-RNP@HCMg+IPTG. Data are presented as mean ± SD, ns: no significance, *$p < 0.05$, **$p < 0.01$ and ***$p < 0.001$ calculated using Student's test or one-way ANOVA.

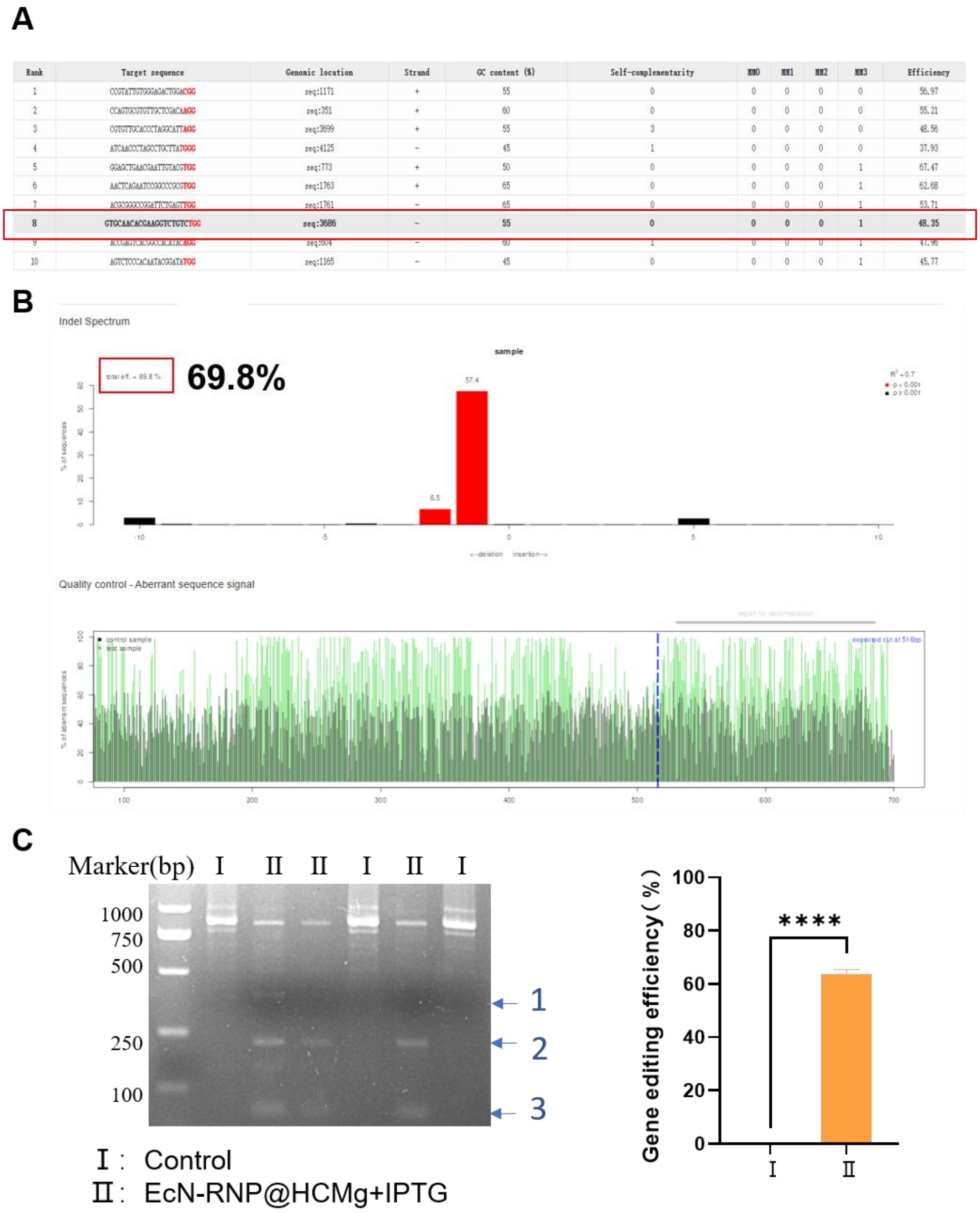

| Rank | Target sequence | Genomic location | Strand | GC content (%) | Self-complementarity | MM0 | MM1 | MM2 | MM3 | Efficiency |
|---|---|---|---|---|---|---|---|---|---|---|
| 1 | CCGTATTGTGGGAGACTGGACGG | seq:1171 | + | 55 | 0 | 0 | 0 | 0 | 0 | 56.97 |
| 2 | CCAGTGCGTGTTGCTCGACAAGG | seq:351 | + | 60 | 0 | 0 | 0 | 0 | 0 | 55.21 |
| 3 | CGTGTTGCACCCTAGGCATTAGG | seq:3699 | + | 55 | 3 | 0 | 0 | 0 | 0 | 48.56 |
| 4 | ATCAACCCTAGCCTGCTTATGGG | seq:4125 | - | 45 | 1 | 0 | 0 | 0 | 0 | 37.93 |
| 5 | GGAGCTGAACGAATTGTACGTGG | seq:773 | + | 50 | 0 | 0 | 0 | 0 | 1 | 67.47 |
| 6 | AACTCAGAATCCGGCCCGCGTGG | seq:1763 | + | 65 | 0 | 0 | 0 | 0 | 1 | 62.68 |
| 7 | ACGCGGGCCGGATTCTGAGTTGG | seq:1761 | - | 65 | 0 | 0 | 0 | 0 | 1 | 53.71 |
| 8 | GTGCAACACGAAGGTCTGTCTGG | seq:3686 | - | 55 | 0 | 0 | 0 | 0 | 1 | 48.35 |
| 9 | [illegible] | seq:604 | - | 60 | 1 | 0 | 0 | 0 | 1 | 47.96 |
| 10 | AGTCTCCCACAATACGGATATGG | seq:1165 | - | 45 | 0 | 0 | 0 | 0 | 1 | 45.77 |



**Figure S23. Analysis of genome editing efficiency, Related to Figure 5.**

A) *In silico* prediction of the gene editing efficiency of the selected sgRNA at the target locus. B) Mutation efficiency detected by TIDE analysis in cells isolated from colonic tissues of mice treated with EcN-RNP@HCMg+IPTG. C) The indel mutation frequency of NOX2 determined by T7EI assay in mouse colonic tissues following EcN-RNP@HCMg+IPTG treatment.

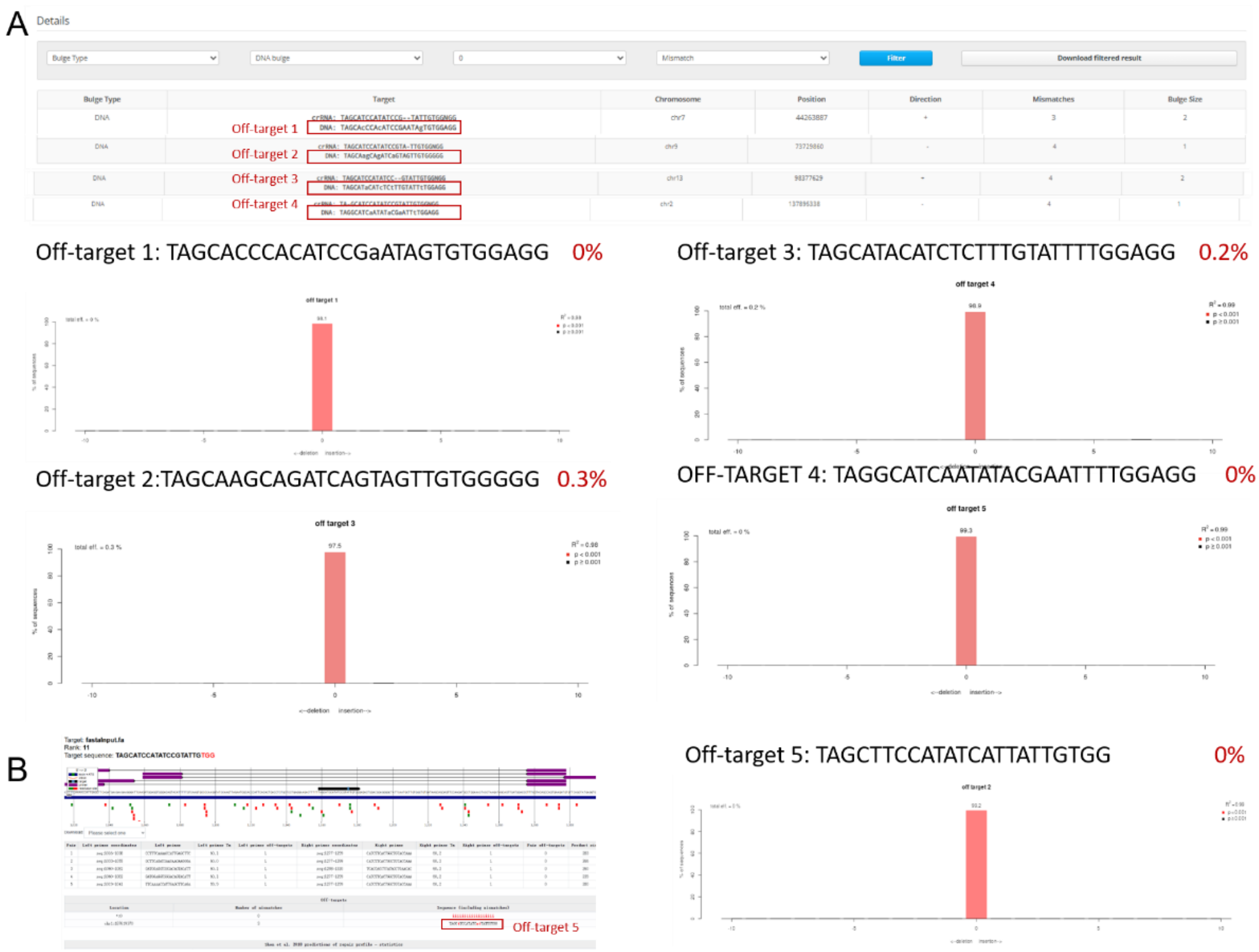


**Figure S24. Analysis of the off-target rate, Related to Figure 5.**

A) Five off-targets of EcN-RNP@HCMg+IPTG were predicted by two computational software programs. B) Analysis of the corresponding mutation frequency of the five off-targets.

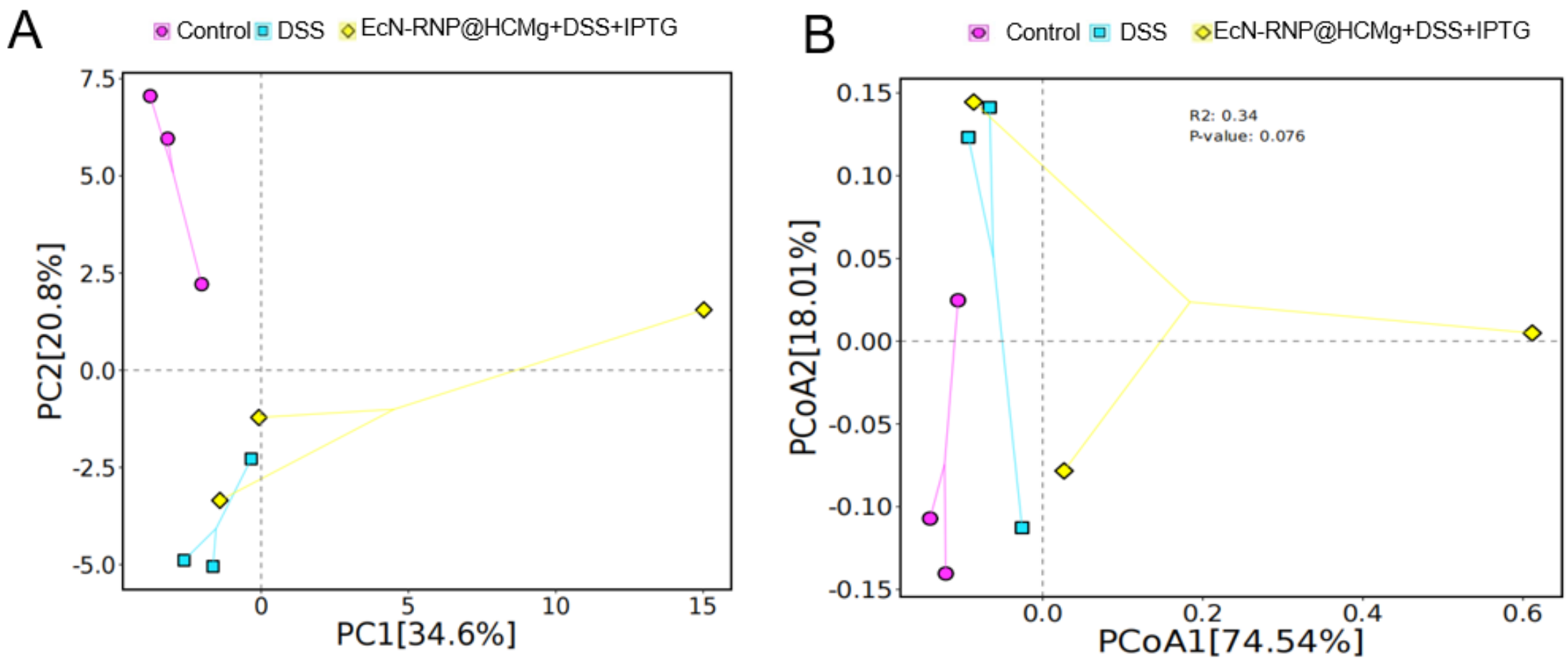


**Figure S25. Beta diversity analysis, Related to Figure 7.**

The results show that the microbial community composition in EcN-RNP@HCMg group exerts a closer relationship with the healthy control group, compared with the DSS group.

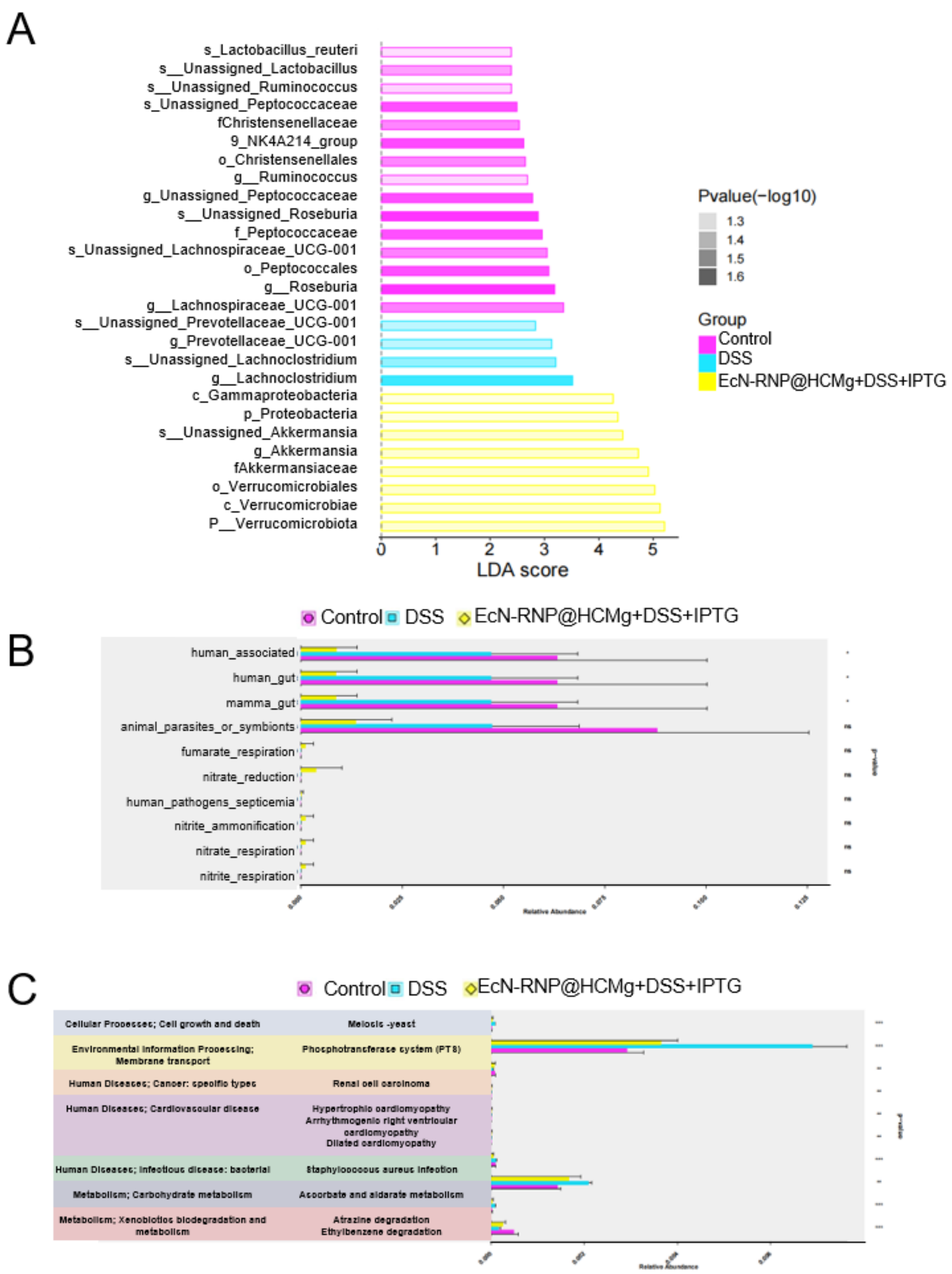


**Figure S26. Gut microbiota functional analysis, Related to Figure 7.**

Functional analysis of bacteria in the colon tissues were performed and the results show that the functions related to the intestines have been restored treated by EcN-RNP@HCMg+IPTG.

**Table S1.**

**Table S1. Primers sequence, Related to STAR Methods.**

| Name | Sequence |
|---|---|
| β-actin(F) | GGCTGTATTCCCCTCCATCG |
| β-actin(R) | CCAGTTGGTAACAATGCCATGT |
| iNOS(F) | GTTCTCAGCCCAACAATACAAGA |
| iNOS(R) | GTGGACGGGTCGATGTCAC |
| IL-1β(F) | GCAACTGTTCCTGAACTCAAC |
| IL-1β(R) | ATCTTTTGGGGTCCGTCAACT |
| IL-10(F) | GCTCTTACTGACTGGCATGA |
| IL-10(R) | CGCAGCTCTAGGAGCATGTG |
| TGF-β2(F) | CTTCGACGTGACAGACGCT |
| TGF-β2(R) | GCAGGGGCAGTGTAAACTTATT |
| IL-6(F) | TAGTCCTTCCTACCCCAATTTCC |
| IL-6 (R) | TTGGTCCTTAGCCACTCCTTC |
| TNF-α(F) | CCCTCACACTCAGATCATCTTCT |
| TNF-α(R) | GCTACGACGTGGGCTACAG |
| Arg-1(F) | CTCCAAGCCAAAGTCCTTAGAG |
| Arg-1(R) | AGGAGCTGTCATTAGGGACATC |
| CD206(F) | CTCTGTTCAGCTATTGGACGC |
| CD206(R) | CGGAATTTCTGGGATTCAGCTTC |
| Gp91$^{phox}$ cell(F) | CCCCAAGGTATCCAAGTT |
| Gp91 $^{phox}$ cell(R) | AAAGGGTCCATCAACTGC |
| Gp91 $^{phox}$ bacteria(F) | CGCCAGGGTTTTCCCAGTCACGACGTT |
| Gp91 $^{phox}$ bacteria(R) | ATGCAGCTGGCACGACAGGTTTCCCGA |
| Cas9(F) | CAAACAGCGCACTTTCGACAATGGAAG |
| Cas9(R) | TGTTCCTTAAGGATTTGGGACCCCAGT |
| Target 1(F) | CTGTATGTGGCCGTGACTCG |
| Target 1(R) | CCTTTCCTGCATCTGGGTCT |
| Off-target 1(F) | CCTCGCCTGGGAGATAGGAG |
| Off-target 1(R) | GCACGGGGATCAGAGGTTTT |
| Off-target 2(F) | TGAGCCTTTTCAGATTTCTGTTCC |
| Off-target 2(R) | ACAAGGATGATGCCTATGGCT |
| Off-target 3(F) | TCTACTCTTGCTCTGCCATACATT |
| Off-target 3(R) | AGCAATATGATGCGCAATCCC |
| Off-target 4(F) | TGACGCCCCCTTGTTTTTCT |
| Off-target 4(R) | CCACCCGTAGGCCTTCATAC |
| Off-target 5(F) | TCTCAGTTTCAGGCCTTCGG |
| Off-target 5(R) | ACATGCAAAGTCGCAAAGCC |
