# Supplementary material for: CRISPR-Cas9-producing probiotic bacteria for editing NOX2/gp91phox and treating inflammatory bowel disease
Source: Cell Rep Med. 2026 Jul 24;7(8):102940. doi: 10.1016/j.xcrm.2026.102940 (PMC13522793; doi:10.1016/j.xcrm.2026.102940)
Supplement: Supplementary file 2 [file mmc2.pdf]

Cell Reports
Medicine

Article

# CRISPR-Cas9-producing probiotic bacteria for editing NOX2/gp91$^{phox}$ and treating inflammatory bowel disease

## Graphical abstract

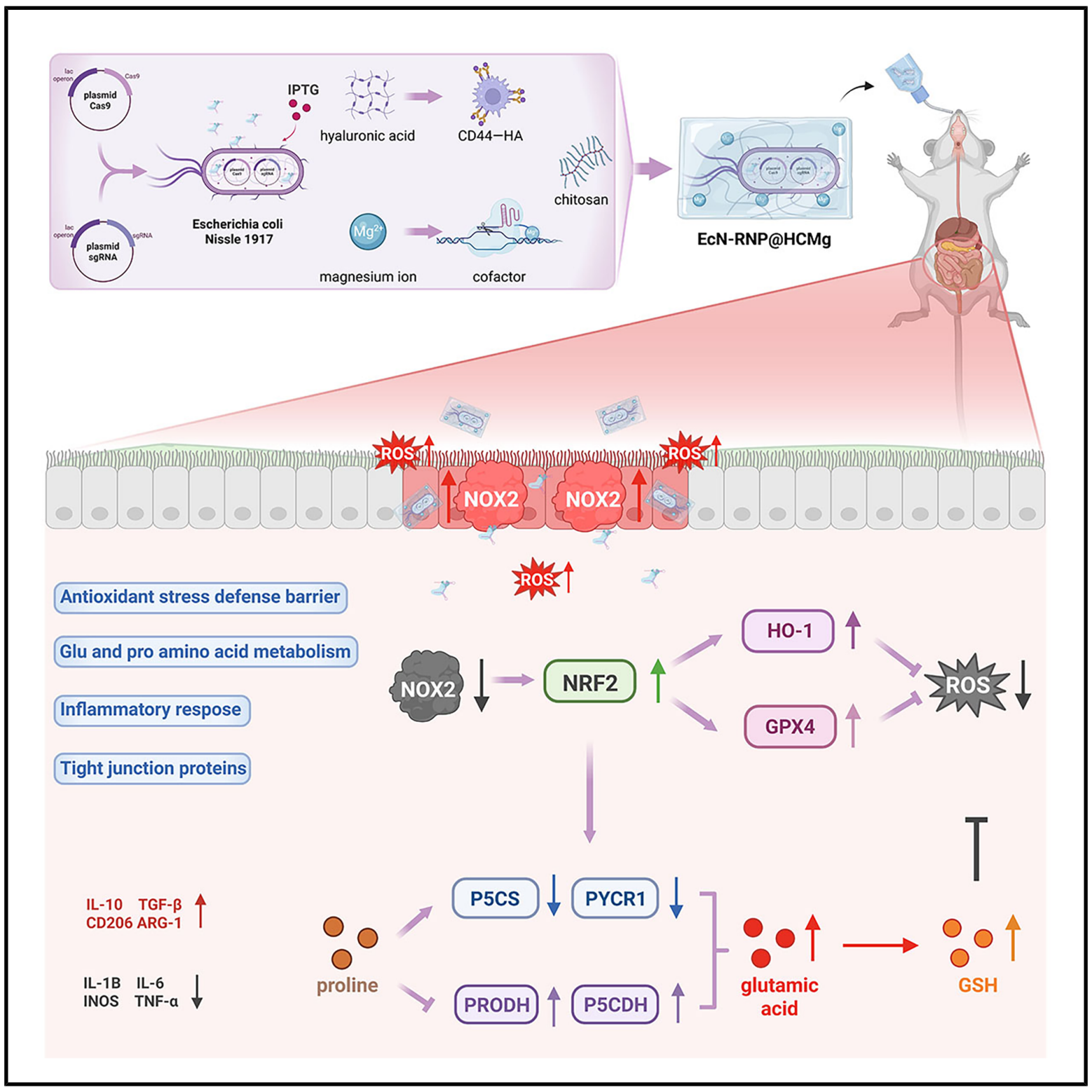


## Authors

Chen Zhang, Tianyu Li, Huoye Hao, ..., Jin Wang, Xiang-Jiao Yang, Xiuli Bi

## Correspondence

xiulibi@lnu.edu.cn

## In brief

Zhang et al. prepare EcN-RNP hydrogel, aiming to treat IBD by inhibiting NOX2 expression via CRISPR-Cas9-mediated genome editing. The hydrogel enhances oxidative stress defense pathways and modulates glutamate and proline metabolism to reduce ROS.

## Highlights

- EcN-RNP hydrogel downregulates NOX2 via genome editing to lower ROS
- EcN-RNP hydrogel boosts antioxidant capacity and glutathione to alleviate colitis
- CRISPR-Cas9-expressing EcN withstands harsh gastrointestinal environments
- Synthetic inducible promoter achieves controllable CRISPR-Cas9 expression

Zhang et al., 2026, Cell Reports Medicine 7, 102940
August 18, 2026 © 2026 The Authors. Published by Elsevier Inc.
https://doi.org/10.1016/j.xcrm.2026.102940

CellPress

Cell Reports Medicine

CellPress
OPEN ACCESS

Article

# CRISPR-Cas9-producing probiotic bacteria for editing NOX2/gp91$^{phox}$ and treating inflammatory bowel disease

Chen Zhang,[1,4,5] Tianyu Li,[1,4] Huoye Hao,[1,4] Gang Wang,[1,4] Yuzhe Zhang,[1,4] Yue Huang,[1,4] Lili Chen,[1,4] Jin Wang,[1,4] Xiang-Jiao Yang,[2] and Xiuli Bi[1,3,4,6,*]

[1]College of Life Science, Liaoning University, Shenyang 110036, China
[2]Rosalind and Morris Goodman Cancer Institute & Department of Medicine, McGill University, Montreal, QC H3A 1A3, Canada
[3]Key Laboratory for Chronic Diseases Molecular Mechanism Research and Nutritional Intervention of Shenyang, Shenyang 110036, China
[4]Present address: 66 Chongshan Road, Shenyang, Liaoning 110036, China
[5]Senior author
[6]Lead contact
*Correspondence: xiulibi@lnu.edu.cn
https://doi.org/10.1016/j.xcrm.2026.102940

## SUMMARY

**The primary pathogenic mechanism of inflammatory bowel disease (IBD) involves elevated levels of reactive oxide species (ROS) in the gut, leading to oxidative stress and damage to the intestinal barrier function. We engineered a non-pathogenic bacterial strain, *Escherichia coli* Nissle 1917 (EcN), for oral CRISPR-Cas9 delivery to edit NOX2 (encoding the gp91$^{phox}$ subunit of NADPH oxidase 2), thereby alleviating IBD symptoms by reducing ROS. EcN expressing the Cas9/sgRNA ribonucleoprotein (RNP) was encapsulated in a hydrogel (composed of hyaluronic acid, chitosan, and $MgCl_2$), which protected EcN-RNP from degradation and increased survival from 0.07% to 12%. EcN-RNP hydrogel reduced NOX2 expression by 47% and ROS by 88% in lipopolysaccharide-induced RAW264.7 cells. Moreover, oral delivery of EcN-RNP hydrogel mitigated inflammation in dextran sodium sulfate-induced colitis mouse models. Mechanistically, the hydrogel could activate the NRF2-HO1/GPX4 pathway by inhibiting NOX2 expression, enhancing oxidative defense, and promoting glutathione accumulation. Thus, the EcN-RNP hydrogel offers a promising therapeutic strategy for IBD.**

## INTRODUCTION

Inflammatory bowel disease (IBD), including Crohn's disease and ulcerative colitis, is a chronic inflammatory disorder involving the colon and small intestine.[1] As a modern epidemic, IBD has emerged as a global condition, affecting approximately 0.3%–0.5% of the world's population. It has shown a rapidly increasing incidence in certain regions and a steadily accumulating prevalence, with an increasing proportion of elderly patients exhibiting the disease.[2,3] These trends underscore the urgent need to develop therapeutic strategies that address the complexity of IBD management. A key pathological hallmark of IBD is the markedly elevated levels of reactive oxygen species (ROS) at lesion sites, 10–100 times higher than the levels in healthy tissues.[3] A large number of immune cells are recruited to the intestine and produce substantial ROS via "respiratory burst." Furthermore, certain pathogenic bacteria can stimulate the host immune system, indirectly driving ROS generation further.[4–7] Elevated ROS also promote the release of pro-inflammatory cytokines, thereby exacerbating intestinal inflammation. Prolonged exposure to high levels of ROS may cause DNA damage and genetic mutations, significantly increasing the risk of colorectal cancer.[8] Consequently, inhibiting ROS accumulation to disrupt pathological inflammatory cascades has been recognized as a promising therapeutic strategy for IBD.

Currently, strategies for reducing ROS levels fall into two categories, one of which involves suppression of the sources of ROS generation and the other involving enhancement of the antioxidant capacity. The latter approach primarily relies on exogenous antioxidant agents, which require continuous supplementation to maintain lowered ROS levels. However, many antioxidants suffer from poor stability, and their oxidation products may interfere unpredictably with normal physiological functions and adversely affect gut microbiota.[9,10] In contrast, targeting the intracellular enzyme systems responsible for ROS generation represents a safer and more effective approach as it addresses the underlying cause of oxidative stress.[11,12] In IBD, respiratory burst that centers on the activation of the NADPH oxidase complex is a major source of ROS. The catalytic subunit of this complex, NOX2, plays a key role in inflammation and has been implicated in the pathogenesis of IBD in experimental mouse models.[13,14] Upon activation, membrane-localized NOX2 catalyzes the reduction of molecular oxygen ($O_2$) to generate superoxide anion ($O_2^{-}\cdot$). Subsequently, these are


© 2026 The Authors. Published by Elsevier Inc.
This is an open access article under the CC BY license (http://creativecommons.org/licenses/by/4.0/).

converted into hydrogen peroxide ($H_2O_2$), which can further react to form highly reactive hydroxyl radicals.[15] NOX2 is a complex consisting of six subunits, including gp91$^{phox}$, p22$^{phox}$, p47$^{phox}$, p67$^{phox}$, p40$^{phox}$, and the small GTPase Rac. Among these, the membrane-bound gp91$^{phox}$ subunit acts as the catalytic core of NADPH oxidation[16] and sequentially recruits other subunits to assemble the active NOX2 complex for initiating ROS production. Therefore, targeted inhibition of the gp91$^{phox}$ subunit of NADPH oxidase, through effectively blocking the initial step in ROS generation, offers a potential strategy for IBD therapy.

Oral administration represents an ideal route for IBD treatment owing to its non-invasiveness, convenience, and high patient compliance.[17,18] The CRISPR-Cas9 system enables highly efficient and precise gene editing and has demonstrated promising therapeutic outcomes in various diseases, including cancer, Alzheimer disease, and sickle cell anemia.[19,20] However, the harsh physicochemical environment of the gastrointestinal tract, characterized by extreme pH and enzymatic degradation, can compromise the activity of CRISPR-Cas9, and a safe and effective oral delivery system is lacking. Notably, CRISPR-Cas9 originates from bacteria, and its production also relies on engineered *Escherichia coli*.[21,22] Therefore, bacteria engineered to express CRISPR-Cas9 possess inherent advantages and hold significant potential as oral delivery vehicles. As a non-pathogenic *E. coli* strain lacking virulence factors, *E. coli* Nissle 1917 (EcN) can modulate mucosal immune responses in the gut and promote the production of anti-inflammatory cytokines. EcN has already been engineered to express therapeutic proteins such as IL-10, superoxide dismutase, and catalase for the treatment of IBD.[17,23,24]

In this study, we designed a CRISPR-Cas9 system to edit the gp91$^{phox}$ gene and then engineered EcN to express this system and form EcN-RNP hydrogel via a synthetic biology approach. This system targeted the gp91$^{phox}$ subunit of NOX2, a key factor in ROS production, aiming to alleviate oxidative stress, modulate glutamate and proline metabolism, and eventually alleviate IBD. EcN protected CRISPR-Cas9 from gastrointestinal degradation and enabled its *in situ* production. EcN-RNP is encapsulated with hyaluronic acid (HA), chitosan (CS), and $MgCl_2$, utilizing the affinity of HA for CD44 receptors to accumulate at inflammatory sites.[25] Isopropyl β-D-1-thiogalactopyranoside (IPTG)-induced expression provided spatiotemporal control and minimized off-target effects. This orally administered engineered EcN could offer an efficient and safe gene therapy approach for IBD.

## RESULTS

### Single-cell RNA sequencing identified NADPH oxidase 2 subunits as therapeutic targets for IBD

To investigate the sources of ROS, single-cell RNA sequencing (scRNA-seq) was performed. The results revealed that differentially expressed genes were primarily enriched in pathways related to oxidative stress, inflammatory response, and ROS metabolism (Figure 1A; Figures S1 and S2). Further analysis revealed that the differentially expressed genes involved multiple NOX genes, suggesting their critical role in the pathogenesis of IBD. NOX2, a key member of the NOX family and the gp91$^{phox}$ subunit of NADPH oxidase 2, serves as a major source of ROS. Building upon the scRNA-seq results, we investigated editing the gp91$^{phox}$ subunit to inhibit NOX2 as a therapeutic strategy for IBD.

### Preparation of EcN-RNP hydrogel targeting the NOX2/gp91$^{phox}$ subunit

To inhibit *NOX2* expression, we designed single guide RNA (sgRNA) sequences targeting gp91$^{phox}$ (a key subunit of NOX2) and constructed a dual-plasmid system comprising pET-22a-Cas9-kana and pUC57-sgRNA-Chl within EcN. PCR amplification yielded distinct fragments corresponding to Cas9 and sgRNA, with molecular weights matching their expected sizes, which confirmed the presence of both plasmids in the strain (Figure 1B; Figure S3). Magnesium ions ($Mg^{2+}$, positively charged), HA (negatively charged), and chitosan (CS, positively charged) were sequentially mixed with bacterial suspension to form a hydrogel (EcN-RNP@HCMg) via electrostatic interactions, resulting in the white precipitate, as depicted at the bottom in Figures 1C and 1A, and a progressive shift in surface charge from negative to positive during the EcN-RNP@HCMg synthesis, as shown by zeta potential analysis, confirming successful bacterial encapsulation (Figure 1D). EcN-RNP@HCMg exhibited the maximum swelling ratio and injectability, characteristics that are required to effectively reduce the risk of intestinal embolism and laceration (Figure S4). An initial bacterial concentration of $10^9$ colony-forming units (CFUs)/mL resulted in the optimal encapsulation efficiency for EcN-RNP@HCMg. Therefore, this concentration was selected for all subsequent experiments (Figure 1E; Figure S5).

### Properties of EcN-RNP hydrogel on gene expression, resistance to gastrointestinal degradation, and prolonged intestinal retention

After the EcN-RNP hydrogel was constructed, its performance was characterized. IPTG binds to the repressor LacI for releasing it from the promoter, which allows RNA polymerase to bind for initiating gene transcription and protein expression. By placing the Cas9 gene under the control of a promoter regulated by the Lac operon, Cas9 expression could be controlled through the addition of IPTG to the engineered EcN, as illustrated in Figure 2A. SDS-PAGE (Figure 2B) and western blot (Figure 2C) analyses confirmed that both EcN-RNP and EcN-RNP@HCMg exhibited significantly enhanced Cas9 expression upon IPTG induction, while negligible expression was detected in the absence of IPTG. Growth curves were plotted to assess the impact of hydrogel encapsulation on EcN growth, EcN-RNP and EcN-RNP@HCMg showed no significant difference, demonstrating that hydrogel encapsulation did not notably interfere with the proliferation of EcN-RNP (Figure 2D). EcN-RNP@HCMg exhibited resistance to degradation by gastrointestinal enzymes and extreme pH conditions due to the inherent properties of the native bacteria and the physical barrier provided by the hydrogel (Figure 2E), enabling effective oral delivery. Upon exposure to simulated gastric fluid (SGF) and simulated intestinal fluid (SIF), the survival rate of the EcN-RNP group was only 0.07%, whereas the EcN-RNP@HCMg showed a

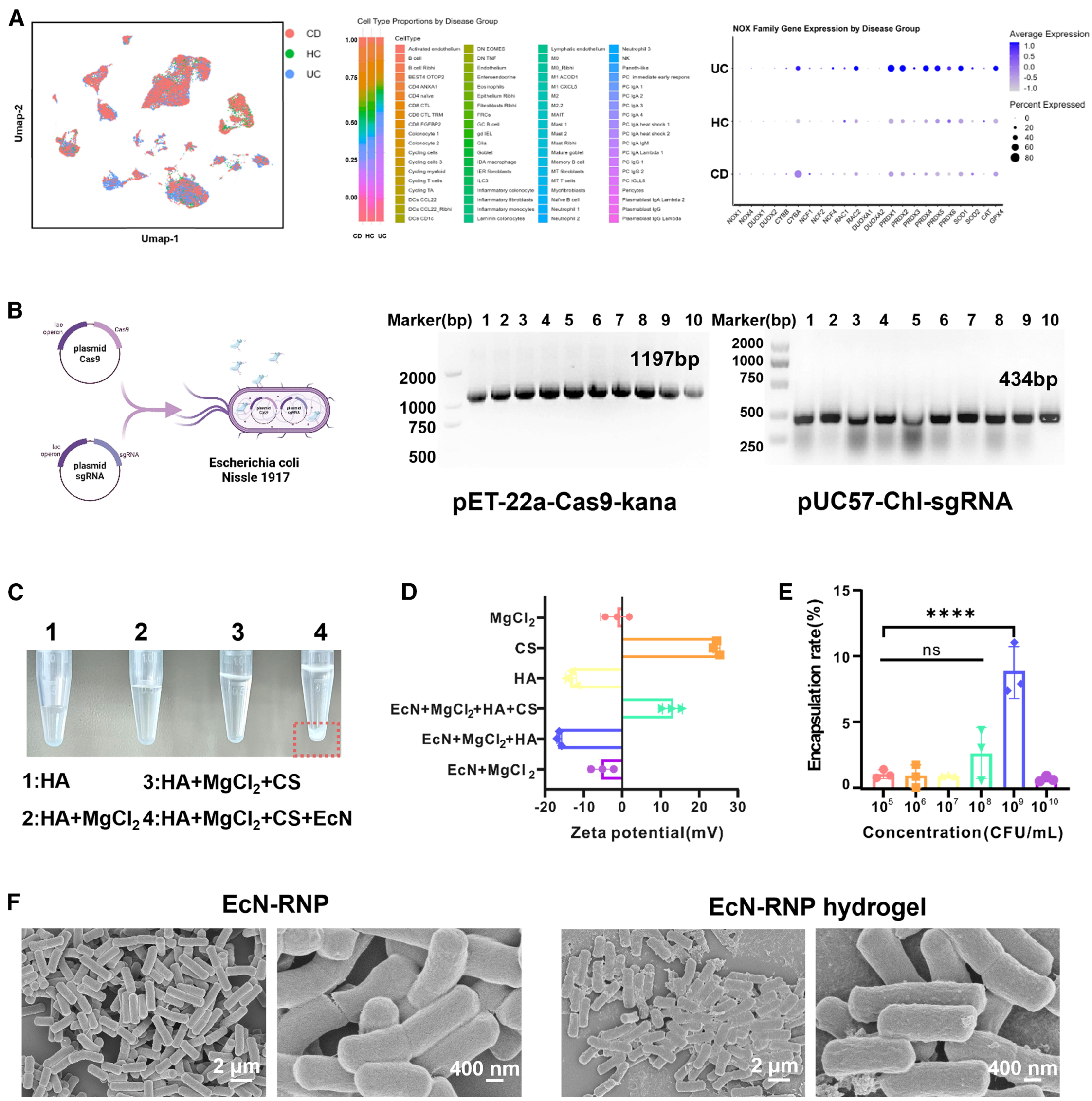


**Figure 1. scRNA-seq analysis and preparation of EcN-RNP hydrogel**

(A) Single-cell clustering, annotation, and NOX expression analysis of samples from healthy control (HC), ulcerative colitis (UC), and Crohn's disease (CD) groups. See Figures S1 and S2 for details.

(B) PCR confirming the successful transition of pET-22a-Cas9-kana and pUC57-sgRNA-Chl into EcN.

(C) Images of the EcN-RNP hydrogel.

(D) Zeta potential of the EcN-RNP hydrogel formation process.

(E) Encapsulation efficiency detections. See Figure S5 for details.

(F) The SEM images of EcN-RNP and EcN-RNP hydrogel.

Data are presented as mean ± SD; ns, no significance; *, $p < 0.05$; **, $p < 0.01$; ***, $p < 0.001$, calculated using Student's $t$ test or one-way ANOVA.

significantly higher survival rate of 12% (Figures 2F and 2G). A washing experiment was conducted to investigate whether hydrogel encapsulation enhances the intestinal retention time. After three washes, $7 \times 10^5$ CFUs of EcN-RNP were retained in the EcN-RNP@HCMg group, compared with only $7.6 \times 10^3$ CFUs retained in the unencapsulated EcN-RNP group, representing an approximately 100-fold difference (Figure 2H). Furthermore, EcN-RNP@HCMg demonstrated a stronger fluorescence intensity than EcN-RNP after a 36-h residence time in the gut (Figures 2I and 2J; Figure S6). *Ex vivo* intestinal fluorescence measurements showed intensities of $1.53 \times 10^7$ and $3.69 \times 10^8$ p/S/cm2/sr/(μw/cm$^2$) for the EcN-RNP group and EcN-RNP@HCMg group, respectively (Figure 2K). Thus, the fluorescence signal of EcN-RNP@HCMg was approximately 20-fold stronger than that of EcN-RNP. The presence of HA in the hydrogel targets CD44$^+$ macrophages, enabling specific accumulation at inflammatory sites. High CD44-expressing cells (Caco-2 and RAW264.7) showed stronger uptake of EcN-EGFP@HCMg

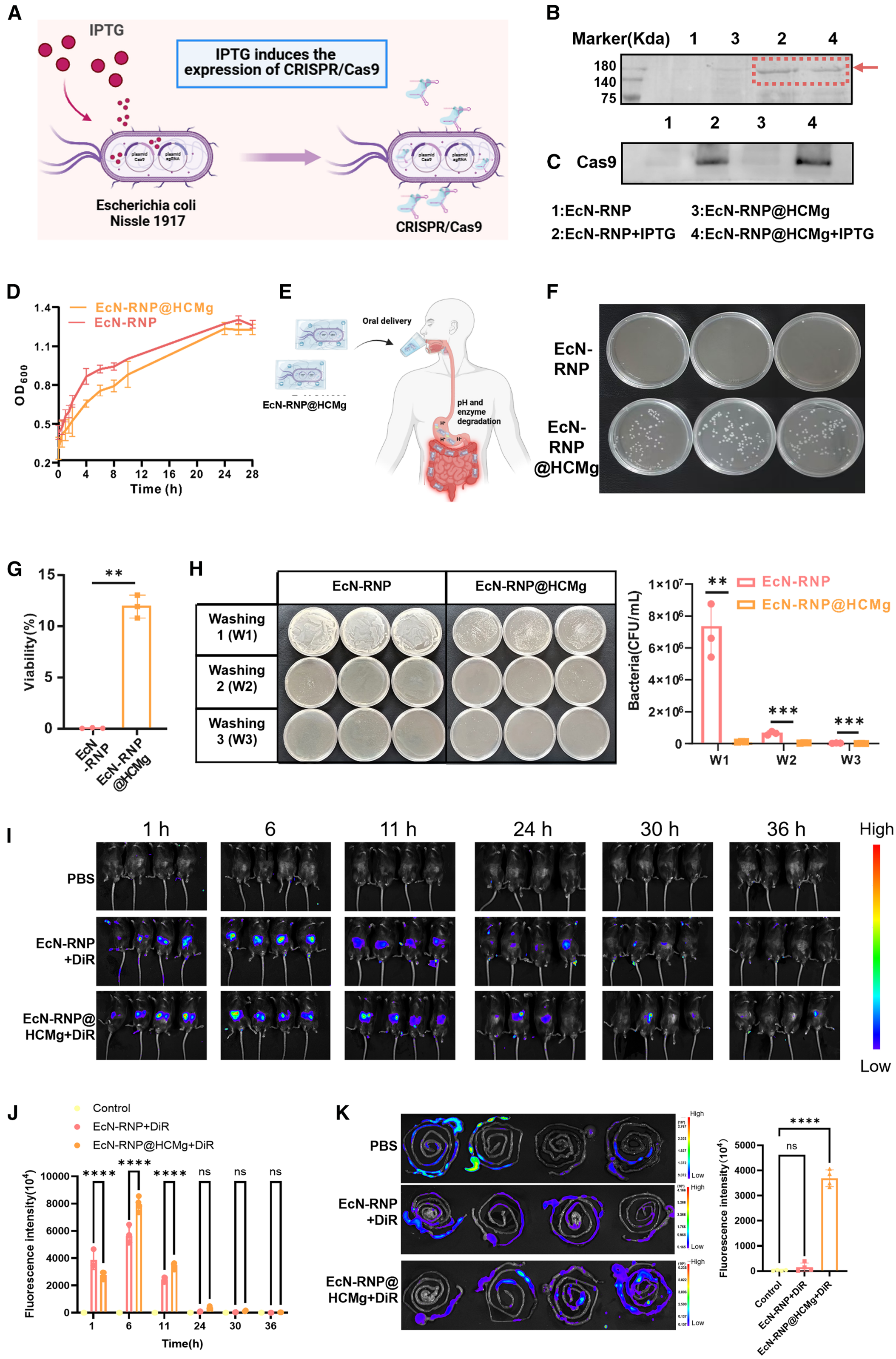


*(legend on next page)*

than low CD44-expressing cells (A549), while free HA/anti-CD44 antibody blocked uptake, confirming CD44-mediated targeting (Figure S6C). This targeting combined with the physical barrier of the hydrogel could significantly enhance the *in vivo* retention time of EcN-RNP.

### Effects of EcN-RNP hydrogel on resistance to cellular oxidative stress and modulation of inflammatory cytokines *in vitro*

Furthermore, we employed CRISPR-Cas9 to edit the NOX2 gene for reducing cellular damage caused by excessive ROS production, as shown in Figure 3A. The intracellular ROS levels were quantified using flow cytometry with the 2',7'-Dichlorodihydrofluorescein diacetate (DCFH-DA) fluorescent probe. The results showed that the $H_2O_2$-activated Caco-2 cells treated with EcN-RNP + IPTG and EcN-RNP@HCMg + IPTG exhibited markedly reduced percentage of fluorescent cells, at 99.74% and 88.42%, respectively, whereas the other groups exhibited more than 60% of these cells (Figures 3B and 3E). ROS levels decreased progressively with the increasing bacterial concentration and prolonged treatment duration (Figure S7). The weaker green fluorescence intensity in the EcN-RNP + IPTG and EcN-RNP@HCMg + IPTG groups were also visually confirmed by fluorescence microscopy (Figures 3C and 3F). A low percentage of red-stained dead cells was noted, accounting for 16.14% and 5.72% of total cells in the EcN-RNP + IPTG and EcN-RNP@HCMg + IPTG groups, respectively. In contrast, a significantly higher proportion of red-stained dead cells were observed in the $H_2O_2$, EcN-RNP + $H_2O_2$, and EcN-RNP@HCMg + $H_2O_2$ groups, accounting for 88.19%, 87.53%, and 69.67% of total cells, respectively (Figures 3D and 3H). Accordingly, cell viability in the EcN-RNP + IPTG and EcN-RNP@HCMg + IPTG groups exceeded 80%, whereas viability in all other groups was lower than 60% (Figure 3G). Collectively, these results indicated that EcN-RNP@HCMg can protect cells against oxidative stress-induced death. Subsequently, we investigated the effect of EcN-RNP@HCMg on the release of inflammatory cytokines in RAW264.7 cells (mouse leukemia cells of monocyte macrophage). EcN-RNP@HCMg significantly decreased the levels of pro-inflammatory cytokines, including IL-1$\beta$, TNF-$\alpha$, IL-6, and iNOS. Concurrently, it increased the level of anti-inflammatory cytokines including TGF-$\beta$, CD206, Arg-1, and IL-10, which demonstrated its ability to suppress inflammation through NOX2 regulation (Figure 3I). Compared with the EcN-RNP@HCCa group (formulated with calcium ions), which showed NOX2 expression levels of 73% (Caco-2 cells) and 75% (RAW264.7 cells), the EcN-RNP@HCMg group exhibited even lower NOX2 expression of 54% (Caco-2 cells) and 53% (RAW264.7 cells) (Figure 3J), which indicated that $Mg^{2+}$ act as an optimal cofactor for Cas9 protein to enhance the gene editing performance. Similarly, the EcN-RNP@HCMg group demonstrated superior ROS suppression (Figure S8) and more effective modulation of inflammatory cytokines (Figure S9) relative to the EcN-RNP@HCCa group. These results collectively highlight the $Mg^{2+}$-enhanced gene editing capability of EcN-RNP@HCMg.

### Therapeutic efficacy of EcN-RNP hydrogel in an IBD mouse model

In addition to investigating the intracellular functions of EcN-RNP hydrogel, its therapeutic effects were also explored in a mouse model. IBD mouse model establishment and intervention strategy are shown in Figure 4A. The body weights of mice in the EcN-RNP@HCMg + IPTG group gradually recovered compared with those of mice in the dextran sulfate sodium (DSS) group, while mice in the EcN-RNP, EcN-RNP + IPTG, and EcN-RNP@HCMg groups continued to exhibit progressive weight loss (Figure 4B). Moreover, the body weights of the mice in the four groups decreased by 21.85%, 10.52%, 25.82%, and 22.90%, respectively, by day 10 (Figure 4C). IPTG-induced EcN-RNP@HCMg group exhibited a better therapeutic efficacy than the EcN-RNP@HCMg group without IPTG. Importantly, IPTG itself is non-toxic and does not affect mouse body weight (Figure S10). Survival curve analysis showed that the mice in EcN-RNP@HCMg + IPTG group exhibited longer survival times (Figure S11). The disease activity index (DAI) scores of mice in the EcN-RNP@HCMg + IPTG group decreased to 0.33, which suggested the better therapeutic efficacy of EcN-RNP@HCMg in IBD (Figure 4D; Figure S12).

Colon length in mice serves as a crucial indicator for assessing the severity of IBD. Figure S13 and Figures 4E and 4F display uniform colon lengths without bleeding in the EcN-RNP@HCMg + IPTG group but significant variation in colon length (both elongation and shortening) in all other groups, with the colon exhibiting signs of redness, swelling, and hemorrhage, indicating that EcN-RNP@HCMg exerts a protective effect against DSS-induced colonic injury. The ratios of colon/body weight and colon weight/length also showed significant increases in the EcN-RNP@HCMg + IPTG group compared with the DSS group (Figure S14). No apparent abnormalities were observed in the major organs (heart, liver, spleen, lung, and kidney) across all groups, indicating that EcN-RNP@HCMg exhibits good *in vivo* safety (Figures S15, S16, and S17). Colonic crypts are intact on the colonic mucosal surface, playing a central role in maintaining intestinal homeostasis. Hematoxylin and eosin (H&E)

**Figure 2. Properties of EcN-RNP hydrogel on gene expression, resistance to gastrointestinal degradation, and prolonged intestinal retention**
(A) Schematic of IPTG-induced Cas9 expression.
(B and C) SDS-PAGE (B) and western blot (C) analyses of Cas9-induced expression.
(D) Bacterial growth curves.
(E) Schematic of oral administration.
(F and G) Bacterial viability (F) and survival rates (G) of EcN-RNP and EcN-RNP@HCMg after treatment with SGF and SIF.
(H) Quantification and statistical analysis of EcN-RNP and EcN-RNP@HCMg retention in the gut following washing experiments.
(I and J) *In vivo* imaging system (IVIS) monitoring of EcN-RNP and EcN-RNP@HCMg intestinal retention over 36 h.
(K) IVIS images of intestines at 36 h post-administration with corresponding fluorescence intensity data. See also Figure S6.
Data are presented as mean ± SD. ns, no significance; *, $p < 0.05$; **, $p < 0.01$; and ***, $p < 0.001$, calculated using Student's $t$ test or one-way ANOVA.

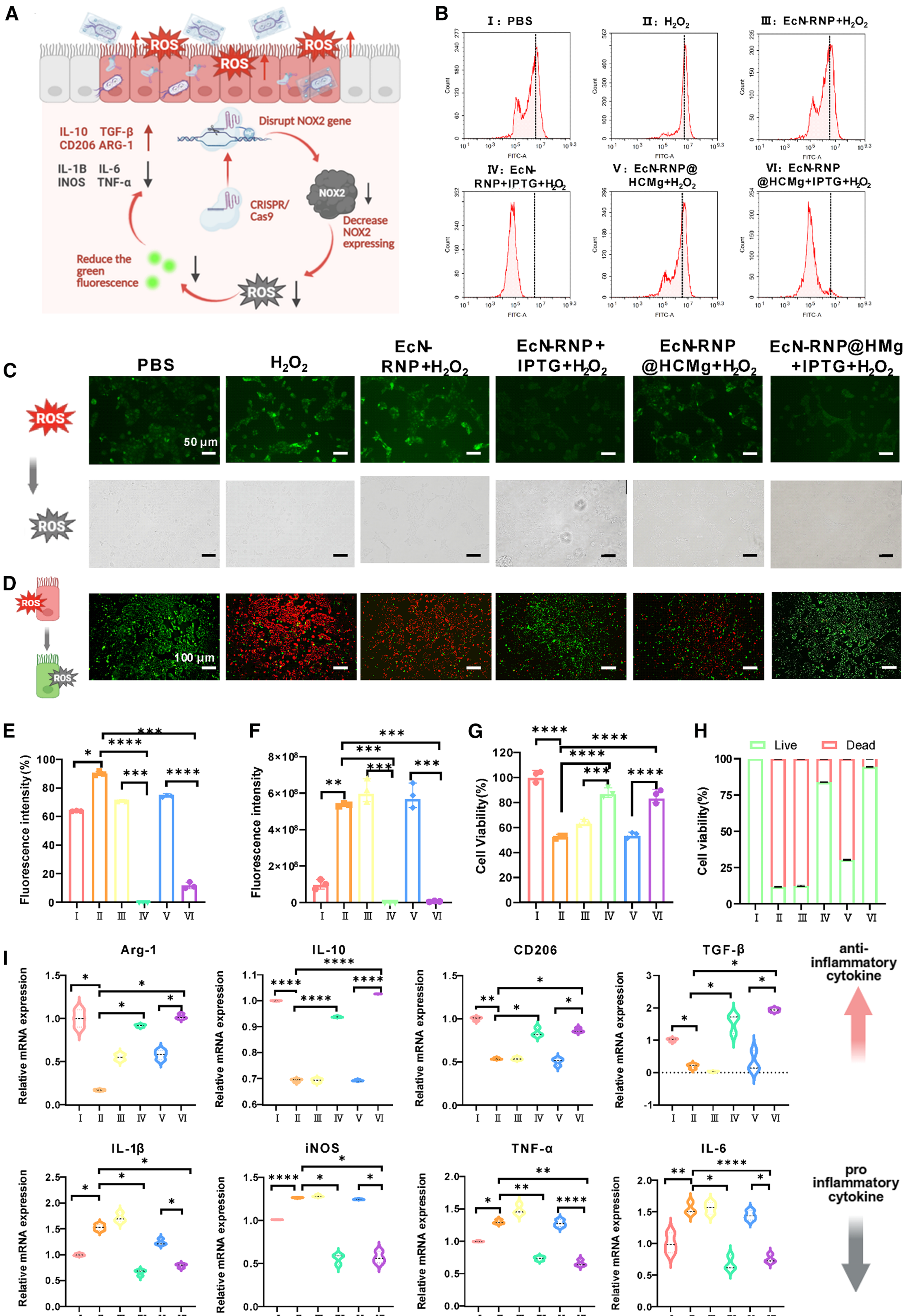


(legend on next page)

staining results indicated that the crypts in the DSS group and other control groups exhibited significant damage and markedly reduced crypt depth (Figures 4G and 4H), while the EcN-RNP@HCMg + IPTG group exhibited partial restoration of the crypt architecture. The key tight junction proteins Claudin-1 and zonula occludens-1 (ZO-1) are core molecules responsible for maintaining the structural integrity of the intestinal epithelial barrier, which is disrupted in IBD, currently the strong brown-stained intensity and high expression of Claudin-1 and ZO-1 exhibited in the EcN-RNP@HCMg + IPTG group, indicating its capacity to repair the intestinal epithelial barrier (Figures S18 and S19).

The percentage of blue staining area was significantly higher in the DSS group (38.27%), EcN-RNP group (43.48%), EcN-RNP + IPTG group (39.26%), and EcN-RNP@HCMg group (41.76%) than in the PBS group, as shown in Figures 4I and 4J. In contrast, significantly reduced blue-stained areas were exhibited by the EcN-RNP@HCMg + IPTG group (25.11%) and EcN-RNP@HCCa + IPTG group (30.33%), indicating that EcN-RNP@HCMg can ameliorate collagen deposition in the colonic tissue. The comparison between EcN-RNP@HCMg + IPTG and EcN-RNP@HCCa + IPTG revealed the better efficacy of $Mg^{2+}$ as an optimal cofactor. In addition, the reverse-transcription PCR (RT-PCR) results showed that the mRNA expression levels of pro-inflammatory cytokines (IL-1β, TNF-α, IL-6, and iNOS) were significantly lower in the EcN-RNP@HCMg + IPTG group, while those of anti-inflammatory cytokines (TGF-β, CD206, Arg-1, and IL-10) exhibited an increasing trend (Figure S20). These findings demonstrate that EcN-RNP@HCMg can balance inflammatory cytokines and maintain the inflammatory homeostasis.

## NRF2-HO1 and NRF2-GPX4 regulation mediated antioxidative stress effects of the EcN-RNP hydrogel

Previous studies have shown that the targeted inhibition $gp91^{phox}$ of NOX2 reduces ROS levels; so, we further investigated the alterations of associated factors involved in the antioxidant mechanism. The NOX2 expression level was increased by 79% in inflammatory RAW264.7 cells induced by lipopolysaccharide (LPS) compared with the PBS group, confirming that NOX2 is associated strongly with cellular inflammation (Figure 5A). Treatments with EcN-RNP + IPTG or EcN-RNP@HCMg + IPTG significantly reduced the NOX2 expression levels to 77% and 44% in RAW264.7 cells, respectively, compared with the LPS-induced group, demonstrating the gene editing efficacy of CRISPR-Cas9. The EcN-RNP@HCMg group also exhibited effective gene editing activity in Caco-2 cells (Figure S21). The protein expression levels of NRF2, HO-1, and GPX4 were elevated to 1.15-, 1.13-, and 1.32-folds, respectively, in the EcN-RNP + IPTG group relative to the LPS-induced group. Similarly, the expression levels of NRF2, HO-1, and GPX4 were increased, reaching 1.26-, 1.23-, and 1.28-folds, respectively, in the EcN-RNP@HCMg + IPTG group, compared with the LPS-induced group. NRF2, HO-1, and GPX proteins are associated with cellular antioxidant defense, confirming the regulatory effect of NOX2 gene editing on the NRF2-HO-1 and NRF2-GPX4 axes (Figures 5C and 5D). The regulatory effect was also observed at the mRNA level (Figure S22). *In vivo* experiments showed that the NOX2 expression in colon tissues of mice was significantly reduced by approximately 40% after treatment with EcN-RNP@HCMg + IPTG, compared with the DSS group mice (Figures 5C and 5D). Mutation efficiency of EcN-RNP@HCMg + IPTG at the target site $gp91^{phox}$ of NOX2 gene increased by more than 40%, and no obvious off-target events were detected (Figures S23 and S24). The expressions of NRF2, HO-1, and GPX4 were significantly upregulated by 21%, 43%, and 10%, respectively, in the EcN-RNP@HCMg group, and the results are consistent with those observed previously at the cellular level. These findings indicate that EcN-RNP@HCMg enhances antioxidant defenses in IBD mice by suppressing NOX2 and activating the NRF2-HO-1 and NRF2-GPX4 signaling pathways (Figures 5C and 5D).

## EcN-RNP hydrogel promotes transformation of proline-to-glutamic acid metabolism

Amino acid metabolism plays a crucial role in IBD development, with various amino acids also being implicated in intracellular oxidative stress.[26–29] Therefore, we performed amino acid profiling analysis to investigate amino acid metabolism variation triggered by the treatment with EcN-RNP@HCMg. The results revealed that the intracellular levels of glutamate, glutamine, and hydroxyproline were significantly reduced in LPS-induced RAW264.7 cells. Notably, EcN-RNP@HCMg treatment could restore their levels (Figure 6A), which demonstrated its therapeutic efficacy. Glutamate is converted to proline via pyrroline-5-carboxylate reductase 1 (PYCR1) and pyrroline-5-carboxylate synthase (P5CS), while proline is converted back to glutamate by proline dehydrogenase (PRODH) and $\Delta^1$-pyrroline-5-carboxylate dehydrogenase (P5CDH) and is converted to hydroxyproline by prolyl hydroxylase (PHD). Western blot results showed that EcN-RNP@HCMg treatment reduced the cellular expression levels of PYCR1 and P5CS to 0.40 and 0.32, respectively,

**Figure 3. Effects of EcN-RNP hydrogel on resisting cellular oxidative stress and modulating inflammatory cytokines**

(A–I) I, PBS; II, $H_2O_2$; III, $H_2O_2$ + EcN-RNP; IV, $H_2O_2$ + EcN-RNP + IPTG; V, $H_2O_2$ + EcN-RNP@HCMg; VI, $H_2O_2$ + EcN-RNP@HCMg + IPTG.
(A) Schematic of the intracellular action of EcN-RNP@HCMg.
(B–E) Flow cytometry analysis of intracellular ROS levels.
(C–F) Fluorescence microscopy images and statistical results showing intracellular ROS levels.
(D–H) Live/dead staining results.
(G) CCK-8 assay.
(I) The mRNA expression levels of inflammatory cytokines.
I, control; II, LPS; III, LPS + EcN-RNP; IV, LPS + EcN-RNP + IPTG; V, LPS + EcN-RNP@HCMg; VI, LPS + EcN-RNP@HCMg + IPTG.
(J) Western blot results of NOX2 from cells treated with EcN-RNP@HCMg + IPTG and EcN-RNP@HCCa + IPTG. See also Figures S8 and S9.
Data are presented as mean ± SD; ns, no significance; *, $p < 0.05$; **, $p < 0.01$; and ***, $p < 0.001$, calculated using Student's *t* test or one-way ANOVA.

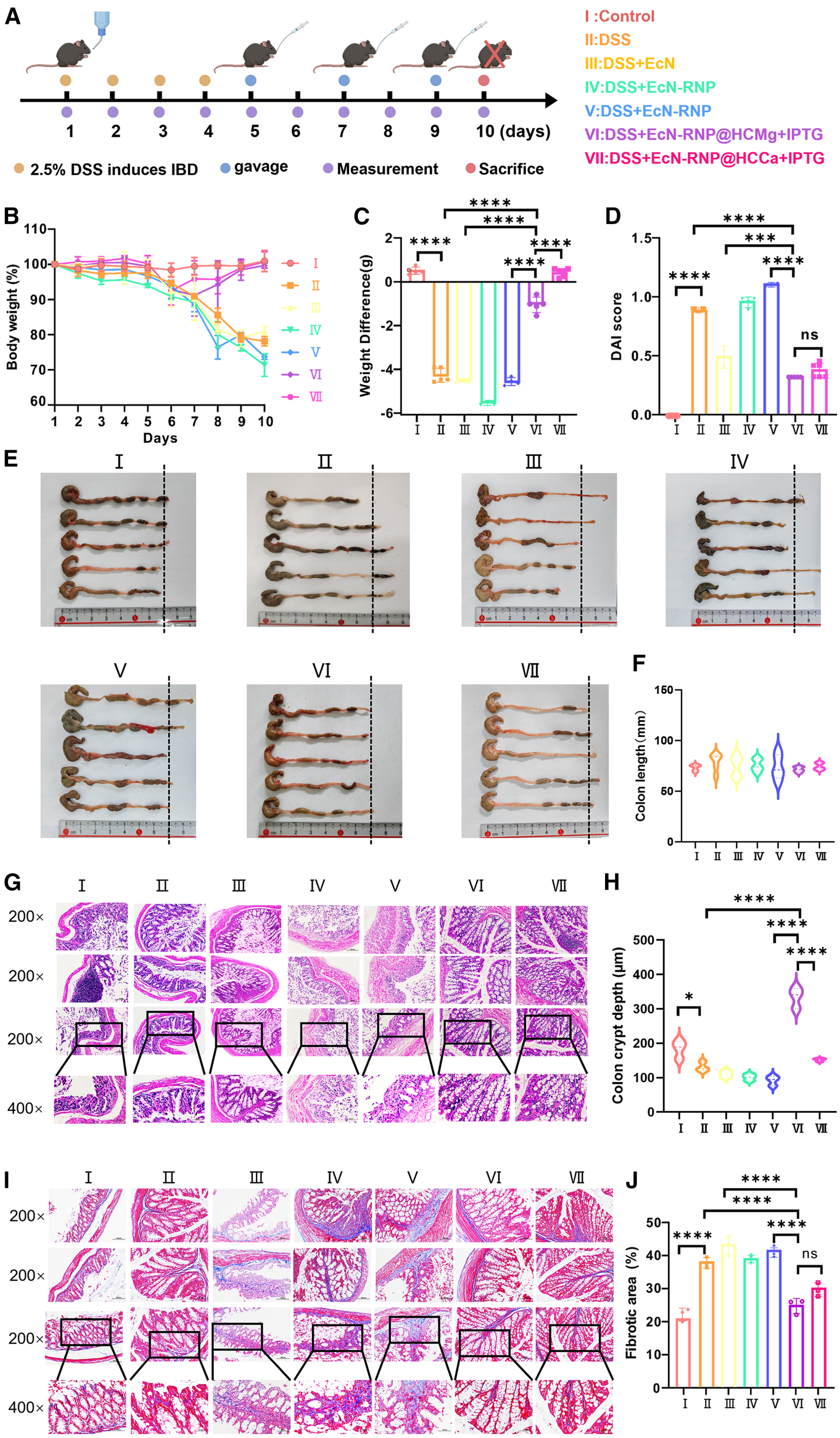


**Figure 4. Therapeutic efficacy of EcN-RNP hydrogel in IBD mouse models**

(A) Schematics of IBD mouse model establishment and intervention strategy.

(B) Body weight variations over time.

(C) Body weights on day 10.

(D) DAIs on day 10. See Figure S12 for details.

(E) Representative images of colons. See also Figure S13.

(F) Colon length analysis.

(G) H&E staining of colon tissues.

(H) Crypt depth quantification.

(I and J) Masson's staining of colon tissues and quantitative analysis of collagen fibers.

I, PBS; II, DSS; III, DSS + EcN; IV, DSS + EcN-RNP; V, DSS + EcN-RNP@HCMg; VI, DSS + EcN-RNP@HCMg + IPTG; VII, DSS + EcN-RNP@HCCa + IPTG.

Data are presented as mean ± SD; ns, no significance; *, $p < 0.05$; **, $p < 0.01$; and ***, $p < 0.001$, calculated using Student's $t$ test or one-way ANOVA.

while increasing the expression levels of PRODH, P5CDH, and PHD to 1.2, 1.24 and 1.33, respectively, compared with the LPS-induced group. This suggests that proline accumulation and cycling help maintain an adequate intracellular supply of glutamate, thereby supporting rapid glutathione (GSH) synthesis (Figures 6B and 6C). Animal experiments also demonstrated similar effects; EcN-RNP@HCMg reduced the expression levels of PYCR1 and P5CS to 0.62 and 0.63 in colon tissue cells of IBD mice, respectively, while increasing the expression levels of PRODH and P5CDH to 1.11 and 1.24, respectively (Figures 6D and 6E). Furthermore, EcN-RNP@HCMg could restore the content of GSH within cells and in the intestines of IBD mice (Figures 6G and 6H). In summary, EcN-RNP@HCMg inhibits proline synthesis and promotes its conversion to glutamate, thus enhancing GSH accumulation against ROS.

## Beneficial effects of EcN-RNP hydrogel on gut microbiota of DSS-induced IBD mouse models

Considering that alterations in the gut microbiota are intimately linked to the onset and progression of IBD, the gut microbiota of the mice treated with oral EcN-RNP@HCMg was investigated. Alpha diversity analysis showed the decreased OTU richness, Shannon index, and Simpson index in the DSS-induced IBD mice compared with those in the control group. These indices were partially restored following treatment with EcN-RNP@HCMg, indicating a recovery of microbial diversity (Figures 7A–7C). Moreover, the microbial community composition after treatment with EcN-RNP@HCMg exhibited a closer relationship with the healthy control group, as shown by beta diversity analysis (Figure 7D; Figure S25). Family-level analysis

CellPress
OPEN ACCESS

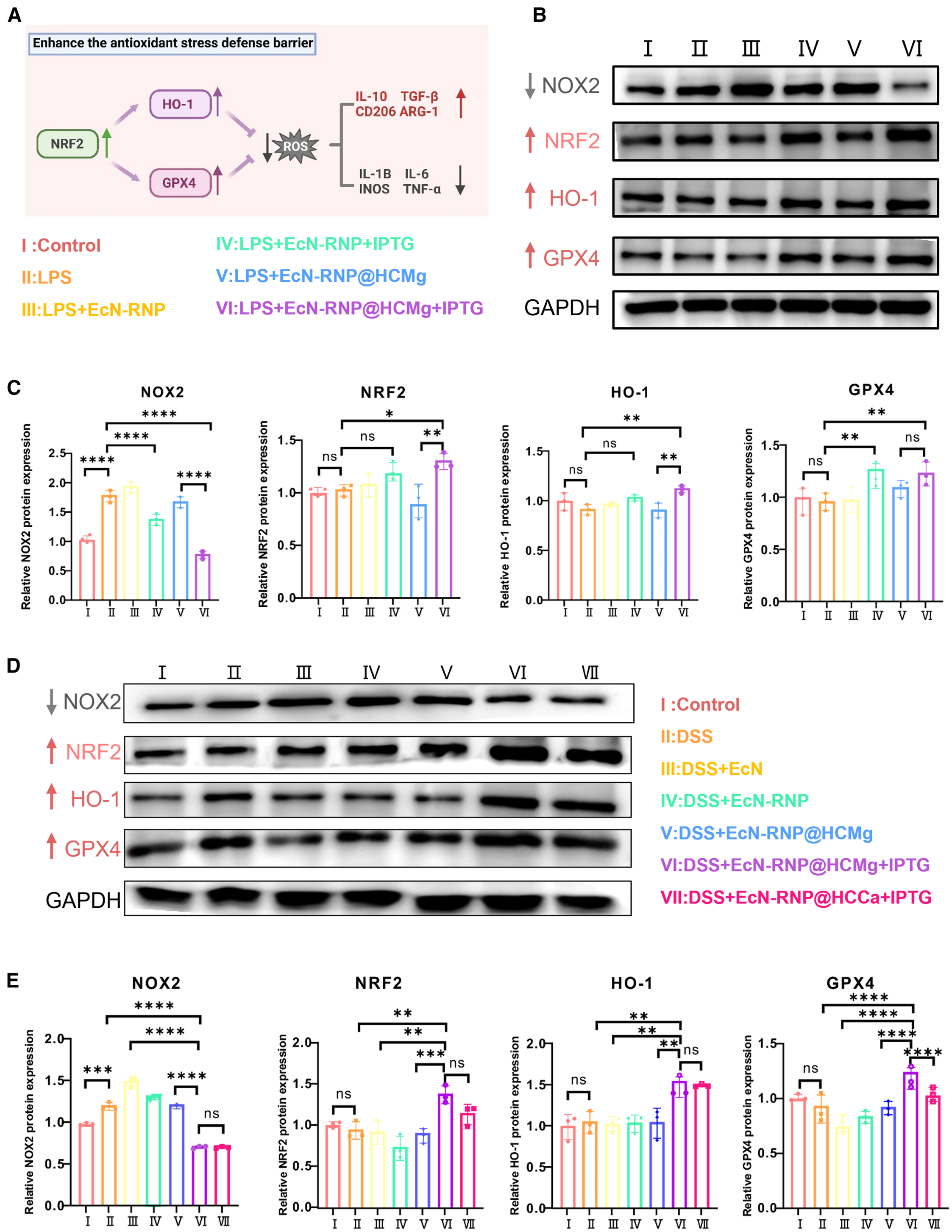


**Figure 5. NRF2-HO1 and NRF2-GPX4 regulation mediated antioxidative stress effects of EcN-RNP hydrogel**

(A) Schematic of the signaling pathway.

(B) Western blot (WB) analysis of NOX2, NRF2,HO-1, and GPX4 expression in the RAW264.7 cells. See also Figure S22.

(C) Statistical analysis of WB results. I, PBS; II, LPS; III, LPS + EcN-RNP; IV, LPS + EcN-RNP + IPTG; V, LPS + EcN-RNP@HCMg; VI, LPS + EcN-RNP@HCMg + IPTG.

(D) WB analysis of NOX2,NRF2,HO-1, and GPX4 expression in colon tissues of IBD mice.

(E) Statistical analysis of WB results. I, PBS; II, DSS; III, DSS + EcN; IV, DSS + EcN-RNP; V, DSS + EcN-RNP@HCMg; VI, DSS + EcN-RNP@ HCMg + IPTG; VII, DSS + EcN-RNP@HCCa + IPTG.

Data are presented as mean ± SD; ns, no significance; *, $p < 0.05$; **, $p < 0.01$; and ***, $p < 0.001$, calculated using Student's *t* test or one-way ANOVA.

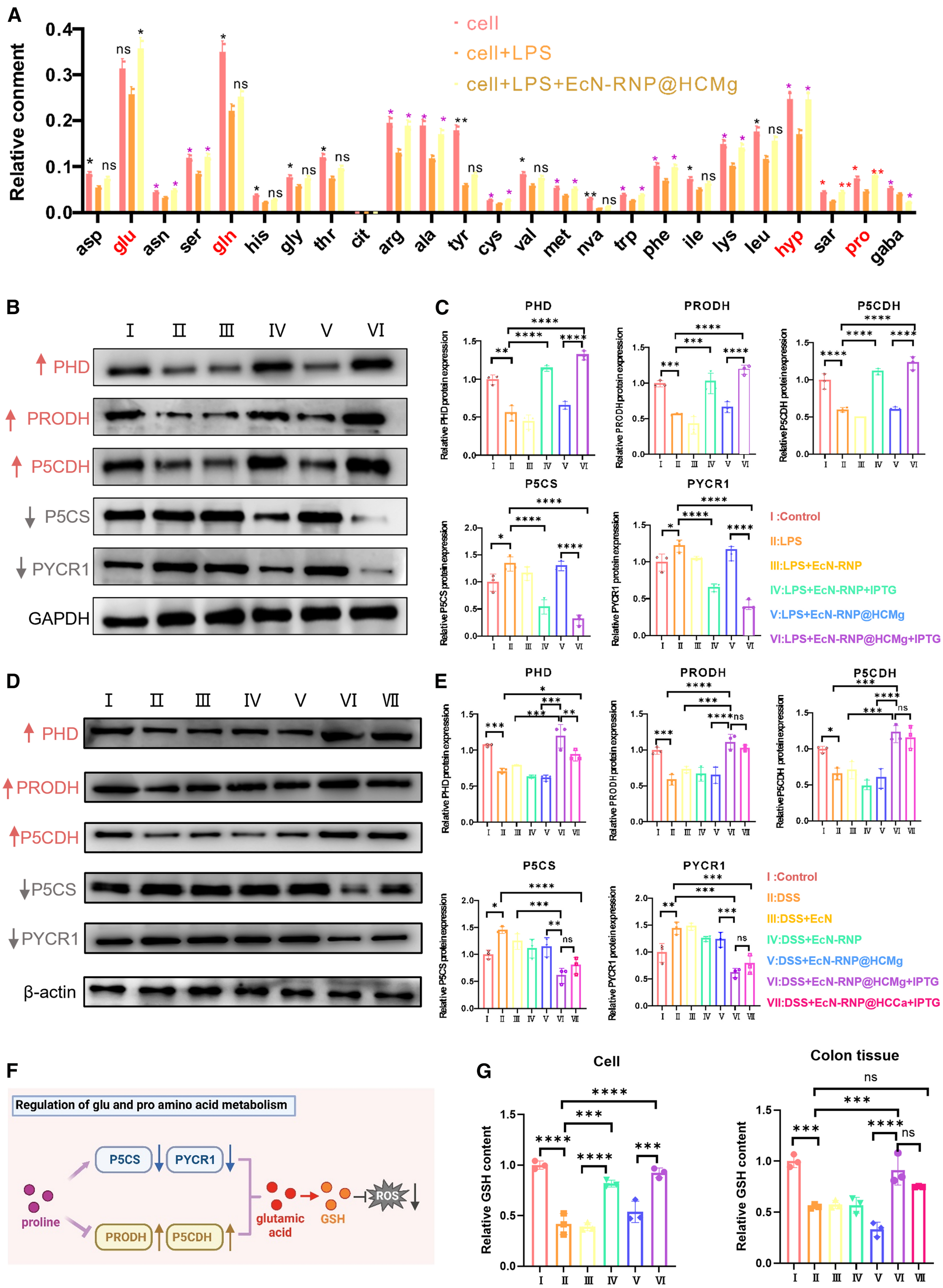

**Figure 6. EcN-RNP hydrogel transforms proline-to-glutamic acid metabolism**

(A) Intracellular amino acid content analysis.

(B and C) Expression levels (B) and statistical analysis (C) of PHD, PRODH, P5CDH, P5CS, and PYCR1 in the treated cells. I, PBS; II, LPS; III, LPS + EcN-RNP; IV, LPS + EcN-RNP + IPTG; V, LPS + EcN-RNP@HCMg; VI, LPS + EcN-RNP@HCMg + IPTG.

*(legend continued on next page)*

revealed that the EcN-RNP@HCMg + IPTG treatment led to an increase in the abundance of multiple bacterial strains compared with the DSS group, as indicated by red arrows in Figure 7E, while decreased abundances of some, as indicated by green arrows. Interestingly, the abundances of beneficial bacteria, *Akkermansia*, *Bacteroides*, and *Eubacterium* xylanophilum group, were increased, while the abundances of *Eubacterium* siraeum group and *Lachnoclostridium* were decreased after treatment with EcN-RNP@HCMg + IPTG (Figures 7F–7L; Figure S26).

## DISCUSSION

In this study, CRISPR-Cas9 was employed to edit gp91$^{phox}$ subunit of NOX2 gene; meanwhile, the conventional CRISPR-Cas9 chassis organisms were replaced with the non-pathogenic EcN. Furthermore, both external encapsulation and internal engineering strategies were employed to modulate EcN behavior. The EcN were coated with CS and HA targeting CD44$^{+}$ macrophages, along with $Mg^{2+}$, to enhance Cas9 activity. Additionally, the hydrogel-based physical barrier was used to protect EcN from gastrointestinal degradation, resulting in a 171-fold increase in EcN survival. Incorporating an inducible promoter module into the Cas9 expression vector resulted in Cas9 expression only in the presence of IPTG, thereby achieving spatiotemporal control and minimizing off-target effects. Using bacteria as a modular engineering platform, precise and complex regulatory circuits can be constructed through the interchangeable integration of genes and promoters. These circuits not only coordinate bacterial population behaviors and synchronize collective activities but also trigger disease-responsive mechanisms, offering IBD treatment application potential.

NOX2 abnormal activation has been shown to contribute to the development of inflammation in IBD mouse models.[13,14] In the present study, CRISPR-Cas9 was employed to suppress gp91$^{phox}$ subunit of NOX2, resulting in a 56% reduction of NOX2 expression levels in cells and a 42% reduction in the IBD mice. Currently, NOX2 inhibition is primarily achieved using small molecules. Dextromethorphan inhibits NOX2 activation by blocking the membrane translocation of p47$^{phox}$; however, it requires an ultra-low dose to exert effect that poses challenges for therapeutic application.[30] Bivalent 2-aminoquinoline compounds have been developed to disrupt the interaction between p47$^{phox}$ and p22$^{phox}$, thereby inhibiting NOX2 activation. However, their efficacy remains limited compared with the direct inhibition of NOX2 assembly.[31] DPI is a non-specific flavoprotein inhibitor capable of inhibiting multiple NOX enzymes including NOX2. However, it is unsuitable for clinical application due to its lack of specificity and potential toxicity.[32] In contrast, the NOX2 inhibition strategy developed in this study offers a well-defined and robust mechanism involving directly disruption of the NOX2 gene and holds greater promise for clinical translation.

Our results showed that the inhibition of NOX2 significantly upregulated the expression of NRF2, HO-1, and GPX4. Specifically, the protein expression levels of NRF2, HO-1, and GPX4 increased by 26%, 23%, and 28% in LPS-induced RAW264.7 cells, respectively, and increased by 21%, 43%, and 10% in colon tissues, respectively, with corresponding increases in mRNA levels. NRF2, a key transcription factor known as the regulator of cellular antioxidant responses, can bind to specific antioxidant response elements in DNA within the nucleus.[33] HO-1 and GPX4 are two critical downstream genes of NRF2. HO-1 generates the potent antioxidant bilirubin and the anti-inflammatory molecule carbon monoxide, thereby providing broad-spectrum protection against ROS-mediated damage.[34] GPX4 specifically reduces lipid hydroperoxides and is a key enzyme in maintaining the cellular membrane integrity.[35] EcN-RNP hydrogel effectively maintains cellular redox homeostasis by modulating the NRF2-HO-1 and NRF2-GPX4 antioxidant defense axes, thereby holding therapeutic potential for IBD.

Various amino acids, including glutamine, arginine, ornithine, citrulline, and tryptophan, and branched-chain amino acids have been found to be dysregulated in IBD patients.[26–29] This study also found that LPS treatment led to a decrease in the levels of glutamate, glutamine, and hydroxyproline in cells, while the levels of these amino acids were restored by treatment with EcN-RNP hydrogel. Glutamate serves as the precursor for GSH, the most important intracellular antioxidant, and glutamine is synthesized from glutamate.[36] Hydroxyproline is synthesized from proline, which is a key component of collagen and essential for maintaining the structural integrity of the intestinal barrier.[37] Glutamate is converted to proline through P5CS and PYCR1, whereas proline is catabolized back to glutamate by PRODH and P5CDH.[38–40] Inhibition of NOX2 triggers the upregulation of NRF2 transcriptional activity, thereby enhancing GPX4 expression. Given that the GPX4 activity is highly dependent on an adequate supply of glutathione, cells respond through a series of metabolic adjustments: the activities of PRODH and P5CDH are promoted, while those of PYCR1 and P5CS are suppressed. This metabolic reprogramming strategy effectively promoted glutamate accumulation. The accumulated glutamate ensured sustained GSH production serving as a key precursor for GSH biosynthesis, thereby maintaining GPX4 activity, efficiently scavenging ROS, and ultimately alleviating cellular oxidative stress. The precise mechanism uncovered in this study suggests a profound interplay between oxidative stress and glutamate metabolism.

Overall, CRISPR-Cas9-mediated suppression of a NOX2 subunit demonstrated promising therapeutic effects in both cell and animal models, highlighting the feasibility of NOX2 as a therapeutic target for IBD. The strategy of using gene editing to suppress NOX2 expression and reduce ROS generation is more stable than conventional antioxidant approaches. Furthermore, this study utilizes EcN as a living therapeutic factory for CRISPR-Cas9, enabling oral delivery by protecting it

(D and E) Expression levels (D) and statistical analysis (E) of PHD, PRODH, P5CDH, P5CS, and PYCR1. I, PBS; II, DSS; III, DSS + EcN; IV, DSS + EcN-RNP; V, DSS + EcN-RNP@HCMg; VI, DSS + EcN-RNP@HCMg + IPTG; VII, DSS + EcN-RNP@HCCa + IPTG.
(G) The relative content of GSH in cells and intestinal tissues treated by different samples.
Data are presented as mean ± SD; ns, no significance; *, $p < 0.05$; **, $p < 0.01$; and ***, $p < 0.001$, calculated using Student's $t$ test or one-way ANOVA.

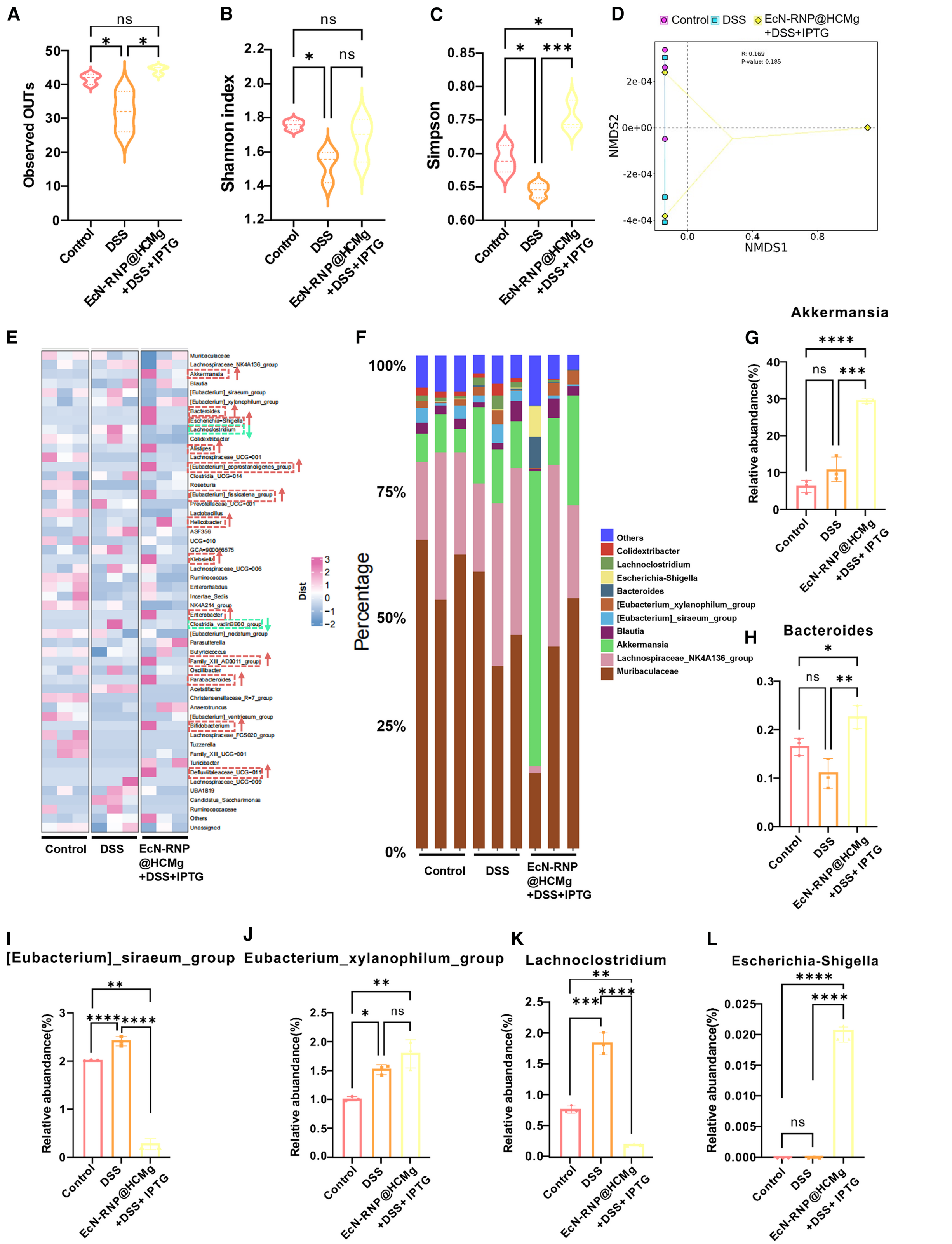


(legend on next page)

from gastroenteric degradation and ensuring sustained *in situ* release. The engineered EcN was designed to carry an IPTG-inducible promoter for enabling controlled gene editing, thereby offering an approach for precise intervention in IBD. EcN-RNP hydrogel could suppress the gp91$^{phox}$ subunit of NOX2 and enhance the oxidative barrier via NRF2-HO-1 and NRF2-GPX4 pathways. Additionally, it could induce amino acid metabolic reprogramming to promote glutamate accumulation, ensure the continuous production of GSH, and efficiently scavenge ROS. Collectively, this study has not only developed an oral administration delivery system using engineered EcN capable of producing CRISPR-Cas9 but also uncovered an elegant regulatory circuit that connects oxidative stress, cellular metabolism, and intestinal inflammation, which may open broad avenues for targeted therapies in IBD.

In subsequent studies, we will further explore the therapeutic effect of the probiotic gene editing strategy constructed in this study on other ROS excess-related diseases, such as stroke, Alzheimer disease, heart failure, liver fibrosis, and rheumatoid arthritis, to provide an experimental basis for the wide application of this strategy. In addition, outer membrane vesicles (OMVs) secreted by EcN were utilized to deliver CRISPR-Cas9 across the intestinal barrier and into host cells. This work established an OMV delivery strategy that enables the OMVs' *in situ* production and encapsulation of CRISPR-Cas9 by probiotics. Given that extracellular vesicles analogous to OMVs have demonstrated promising therapeutic potential in IBD,[3] the therapeutic value and application prospects of probiotic-derived OMVs warrant further in-depth investigation.

## Limitations of the study

First, although we verified the favorable *in vivo* biosafety of EcN-RNP hydrogel based on the measurements of mouse body weights, visceral organ coefficients, and histological results, further safety assessments in non-human primates are required in follow-up research. Second, EcN-RNP hydrogel achieved a gene editing efficiency of over 60% and effectively inhibited NOX2 expression, but sgRNA sequences with better specificity and editing efficiency deserve further investigation to improve the performance of gene editing. Third, five potential off-target sites of the system have been predicted and analyzed, but deep sequencing methods such as genome-wide unbiased identification of DSBs enabled by sequencing (GUIDE-seq) and amplicon sequencing (Amplicon-seq) will improve safety assessments by avoiding low-frequency off-target effects. Finally, although EcN has a well-defined genetic background and mature expression systems, its clinical translational potential is limited due to relatively low abundance in the intestinal microbiota. Future work will develop CRISPR-Cas9 systems in conventional probiotic strains including *Bacillus licheniformis*, *Bifidobacterium*, and *Lactobacillus thermophilus* to expand the application of this delivery system.

## RESOURCE AVAILABILITY

### Lead contact

Requests for further information and resources should be directed to and will be fulfilled by the lead contact, Xiuli Bi (xiulibi@lnu.edu.cn).

### Materials availability

All unique/stable reagents generated in this study are available from the lead contact, with a completed material(s) transfer agreement.

### Data and code availability

- Raw and analyzed data have been deposited in the GEO database under the accession number GEO: GSE214695.
- All data reported in this paper will be shared by the lead contact upon request.
- This paper does not report original code.
- Any additional information required to reanalyze the data reported in this paper is available from the lead contact upon request.

## ACKNOWLEDGMENTS

This work was funded by the National Natural Science Foundation of China (32501261), Liaoning Province Joint Project (2024-BSLH-103), Basic Research Project of the Education Department of Liaoning Province and Research Platform Construction Project of the Education Department of Liaoning Province in 2024. This work was supported by the technical platform services of the Analysis and Testing Center, College of Life Sciences, Liaoning University. We are grateful to all staff members for their professional guidance and experimental assistance.

## AUTHOR CONTRIBUTIONS

Conceptualization, C.Z. and X.B.; methodology, C.Z., T.L., H.H., and G.W.; investigation, C.Z., T.L., Y.Z., and Y.H.; visualization, T.L. and H.H.; supervision, C.Z., X.B., L.C., and J.W.; writing – original draft, C.Z. and T.L.; writing – review & editing, C.Z., X.B., and X.-J.Y.; funding acquisition, C.Z. and X.B.

## DECLARATION OF INTERESTS

The authors declare no competing interests.

## STAR★METHODS

Detailed methods are provided in the online version of this paper and include the following:

- KEY RESOURCES TABLE
- EXPERIMENTAL MODEL AND STUDY PARTICIPANT DETAILS
  - Mice samples
  - Microbe strains
  - Cell lines
- METHOD DETAILS
  - Materials
  - Single-cell transcriptomic analysis
  - Engineering EcN expressing CRISPR/Cas9

**Figure 7. Gut microbiota analysis**
(A–C) Alpha diversity analysis.
(D) Beta diversity analysis. See Figures S25 and S26 for details.
(E and F) Differential analysis at the family level (E) and the genus level (F).
(G–L) Differential analysis of bacterial strains.
Data are presented as mean ± SD; ns, no significance; *, $p < 0.05$; **, $p < 0.01$; and ***, $p < 0.001$, calculated using Student's *t* test or one-way ANOVA.

  - ○ Preparation of EcN-RNP hydrogel and measurement of zeta potential, encapsulation efficiency, growth curve
  - ○ Isopropyl β-D-1-thiogalactopyranoside (IPTG)-induced Cas9 expression assay
  - ○ Detection of intestinal stability and retention time
  - ○ Detection of intracellular ROS levels using flow cytometry and fluorescence microscopy
  - ○ Live/dead staining and cell counting Kit-8 detection
  - ○ RT-PCR detection of inflammatory factors
  - ○ Western blotting assay
  - ○ EcN-RNP hydrogel induced transformation of proline-to-glutamic acid metabolism
  - ○ Animal experiments
  - ○ Detection of gene editing efficiency
- QUANTIFICATION AND STATISTICAL ANALYSIS

## SUPPLEMENTAL INFORMATION

Supplementary data related to this article can be found online at https://doi.org/10.1016/j.xcrm.2026.102940.

Received: March 10, 2026
Revised: May 18, 2026
Accepted: June 29, 2026
Published: July 24, 2026

## REFERENCES

1. Neurath, M.F. (2020). Host–microbiota interactions in inflammatory bowel disease. Nat. Rev. Gastroenterol. Hepatol. *17*, 76–77. https://doi.org/10.1038/s41577-019-0268-7.

2. Windsor, J.W., Hracs, L., Gorospe, J., Coward, S., Buie, M.J., Quan, J., Markovinovic, A., Caplan, L., Cummings, M., Gearry, R., et al. (2023). Sa1770 the global evolution of inflammatory bowel disease across four epidemiologic stages: a systematic review of incidence and prevalence studies over the past century. Gastroenterology *164*, S-423–424. https://doi.org/10.1016/S0016-5085(23)01984-4.

3. Liu, H., Sun, J., Wang, M., Wang, S., Su, J., and Xu, C. (2023). Intestinal organoids and organoids extracellular vesicles for inflammatory bowel disease treatment. Chem. Eng. J. *465*, 142842. https://doi.org/10.1016/j.cej.2023.142842.

4. Huang, B., Yin, T., Fu, S., Liu, L., Yang, C., Zhou, L., Liu, X., Zhuang, H., Cao, Z., and Hua, Z. (2024). Inflammation-oriented montmorillonite adjuvant enhanced oral delivery of anti-TNF-α nanobody against inflammatory bowel disease. Proc. Natl. Acad. Sci. USA *121*, e2320482121. https://doi.org/10.1073/pnas.2320482121.

5. Wang, B., Guo, X., Qin, L., He, L., Li, J., Jin, X., Chen, D., and Ge, G. (2025). Pharmacological modulation of mitochondrial function as novel strategies for treating intestinal inflammatory diseases and colorectal cancer. J. Pharm. Anal. *15*, 101074. https://doi.org/10.1016/j.jpha.2024.101074.

6. Chandrasekharan, B., Saeedi, B.J., Alam, A., Houser, M., Srinivasan, S., Tansey, M., Jones, R., Nusrat, A., and Neish, A.S. (2019). Interactions between commensal bacteria and enteric neurons, via FPR1 induction of ROS, increase gastrointestinal motility in mice. Gastroenterology *157*, 179–192.e2. https://doi.org/10.1053/j.gastro.2019.03.045.

7. Di Petrillo, A., Onali, S., Era, B., Raho, N., Murtas, D., Maxia, C., Favale, A., Eleonora, G., Pinto, S., Fais, A., and Fantini, M.C. (2024). P011 altered expression and enzymatic activity of xanthine oxidase in inflammatory bowel disease. J. Crohns Colitis *18*, i257. https://doi.org/10.1093/ecco-jcc/jjad212.0141.

8. Liu, J., Wang, Y., Heelan, W.J., Chen, Y., Li, Z., and Hu, Q. (2022). Mucoadhesive probiotic backpacks with ROS nanoscavengers enhance the bacteriotherapy for inflammatory bowel diseases. Sci. Adv. *8*, 8798. https://doi.org/10.1126/sciadv.abp8798.

9. Kariyawasam, V., Davis, T., Ghali, M., Tejcek, D., R O'Neil, T., Verley, E., Griffiths, M., Campos, M., Bonney, I., Bulmer, A.C., et al. (2026). Myeloperoxidase luminol reaction—a novel faecal assay for predicting colonoscopy findings in patients with ulcerative colitis: a pilot cross-sectional clinical study. Adv. Healthcare Mater. *15*, e01825. https://doi.org/10.1002/adhm.202501825.

10. Xu, M., Xin, W., Xu, J., Wang, A., Ma, S., Dai, D., Wang, Y., Yang, D., Zhao, L., and Li, H. (2025). Biosilicification-mimicking chiral nanostructures for targeted treatment of inflammatory bowel disease. Nat. Commun. *16*, 2551. https://doi.org/10.1038/s41467-025-57890-8.

11. Jin, T., Lu, H., Zhou, Q., Chen, D., Zeng, Y., Shi, J., Zhang, Y., Wang, X., Shen, X., and Cai, X. (2024). $H_2S$-releasing versatile montmorillonite nanoformulation trilogically renovates the gut microenvironment for inflammatory bowel disease modulation. Adv. Sci. *11*, e2308092. https://doi.org/10.1002/advs.75007.

12. Ge, J., Jia, B., Wang, Y., Ma, Y., Sun, X., Dong, J., Jiang, S., and Li, Z. (2024). DNA nanostructures treat inflammatory bowel disease through ROS scavenging and gut microbiota modulation. Adv. Funct. Mater. *34*, 2402781. https://doi.org/10.1002/adfm.202402781.

13. Bao, S., Carr, E.D.J., Xu, Y.-H., and Hunt, N.H. (2011). Gp91phox contributes to the development of experimental inflammatory bowel disease. Immunol. Cell Biol. *89*, 853–860. https://doi.org/10.1038/icb.2011.4.

14. Chen, L., Pang, W., Luo, X., Li, T., Ju, Q., Yang, Z., He, X., Li, X., Ge, Y., and Bi, X. (2025). Protective effect of a novel nonsteroidal organoselenium compound SLL-1-43 on ulcerative colitis through down-regulating NOX2 signaling pathway in vitro and in vivo. Phytomedicine *143*, 156899. https://doi.org/10.1016/j.phymed.2025.156899.

15. Chen, Y., Shui, M., Yuan, Q., Vong, C.T., Yang, Z., Chen, Z., and Wang, S. (2023). Wielding the double-edged sword: Redox drug delivery systems for inflammatory bowel disease. J. Contr. Release *358*, 510–540. https://doi.org/10.1016/j.jconrel.2023.05.007.

16. Vermot, A., Petit-Härtlein, I., Smith, S.M.E., and Fieschi, F. (2021). NADPH Oxidases (NOX): an overview from discovery, molecular mechanisms to physiology and pathology. Antioxidants *10*, 890. https://doi.org/10.3390/antiox10060890.

17. Brown, T.D., Whitehead, K.A., and Mitragotri, S. (2019). Materials for oral delivery of proteins and peptides. Nat. Rev. Mater. *5*, 127–148. https://doi.org/10.1038/s41578-019-0156-6.

18. Li, S., Jiang, W., Zheng, C., Shao, D., Liu, Y., Huang, S., Han, J., Ding, J., Tao, Y., and Li, M. (2020). Oral delivery of bacteria: Basic principles and biomedical applications. J. Contr. Release *327*, 801–833. https://doi.org/10.1016/j.jconrel.2020.09.011.

19. Yang, X., Bui, T.A., Mei, H., Aksoy, Y.A., Deng, F., Hutvagner, G., and Deng, W. (2024). Exploring the potential and challenges of CRISPR delivery and therapeutics for genetic disease treatment. Adv. Funct. Mater. *34*, 2402630. https://doi.org/10.1002/adfm.202402630.

20. Lin, S., Han, S., Wang, X., Wang, X., Shi, X., He, Z., Sun, M., and Sun, J. (2024). Oral microto-nano genome-editing system enabling targeted delivery and conditional activation of CRISPR-Cas9 for gene therapy of inflammatory bowel disease. ACS Nano *18*, 25657–25670. https://doi.org/10.1021/acsnano.4c07750.

21. Iwasaki, R.S., Ozdilek, B.A., Garst, A.D., Choudhury, A., and Batey, R.T. (2020). Small molecule regulated sgRNAs enable control of genome editing in E. coli by Cas9. Nat. Commun. *11*, 1394. https://doi.org/10.1038/s41467-020-15226-8.

22. Jiang, W.Y., David, B., David, C., Zhang, F., and Marraffini. (2013). RNA-guided editing of bacterial genomes using CRISPR-Cas systems. Nat. Biotechnol. *31*, 233–239. https://doi.org/10.1038/nbt.2508.

23. Zhang, J., Liu, M.Y., Chen, Q.F., Li, X., Chen, L., Dong, Z., Zhu, W., Yang, Y., Liu, Z., and Chen, Q. (2022). Programmable probiotics modulate inflammation and gut microbiota for inflammatory bowel disease treatment after effective oral delivery. Nat. Commun. *13*, 3432. https://doi.org/10.1038/s41467-022-31171-0.

24. Cui, M., Pang, G., Zhang, T., Sun, T., Zhang, L., Kang, R., Xue, X., Pan, H., Yang, C., Zhang, X., et al. (2021). Optotheranostic nanosystem with phone visual diagnosis and optogenetic microbial therapy for ulcerative colitis at-home care. ACS Nano *15*, 7040–7052. https://doi.org/10.1021/acsnano.1c00135.

25. Garrido-Trigo, A., Corraliza, A.M., Veny, M., Dotti, I., Melón-Ardanaz, E., Rill, A., Crowell, H.L., Corbí, Á., Gudiño, V., Esteller, M., et al. (2023). Macrophage and neutrophil heterogeneity at single-cell spatial resolution in human inflammatory bowel disease. Nat. Commun. *14*, 4506. https://doi.org/10.1038/s41467-023-40156-6.

26. Li, J.Y., Guo, Y.C., Zhou, H.F., Yue, T.T., Wang, F.X., Sun, F., and Wang, W.Z. (2023). Arginine metabolism regulates the pathogenesis of inflammatory bowel disease. Nutr. Rev. *81*, 578–586. https://doi.org/10.1093/nutrit/nuac070.

27. Ding, Y., Yu, J., Chen, X., Wang, S., Tu, Z., Shen, G., Wang, H., Jia, R., Ge, S., Ruan, J., et al. (2021). Dose-dependent carbon-dot-induced ROS promote uveal melanoma cell tumorigenicity via activation of mTOR signaling and glutamine metabolism. Adv. Sci. *8*, 2002404. https://doi.org/10.1002/advs.202002404.

28. Lei, L., Cai, S., Zhang, Y., Yang, L., Deng, J., Mei, H., Zhang, X., Zhang, K., He, B., and Cao, J. (2022). Structure inversion-bridged sequential amino acid metabolism disturbance potentiates photodynamic-evoked immunotherapy. Adv. Funct. Mater. *32*, 2103394. https://doi.org/10.1002/adfm.202103394.

29. Wang, Y., Zhang, J., Ren, S., Sun, D., Huang, H.Y., Wang, H., Jin, Y., Li, F., Zheng, C., Yang, L., et al. (2019). Branched-chain amino acid metabolic reprogramming orchestrates drug resistance to EGFR tyrosine kinase inhibitors. Cell Rep. *28*, 512–525.e6. https://doi.org/10.1016/j.celrep.2019.06.026.

30. Li, Y., Hou, L., Cheng, K., He, G., Hong, J.S., Li, S., Zhao, J., and Guo, Y. (2025). Macrophage NADPH oxidase inhibition by ultralow-dose dextromethorphan alleviates DSS-induced inflammation and colitis. Free Radic. Biol. Med. *239*, 189–201. https://doi.org/10.1016/j.freeradbiomed.2025.07.044.

31. Zang, J., Felix, P., Yves, C., Cifuentes-Pagano, E., Hissabu, M.M.S., Dustin, C.M., Svensson, L.H., Olesen, M.M., Poulsen, M.F.L., and Jacobsen, S. (2023). Targeting NOX2 with bivalent small-molecule p47phox-p22phox inhibitors. J. Med. Chem. *66*, 14963–15005. https://doi.org/10.1021/acs.jmedchem.3c01548.

32. Regmi, S.C., Park, S.Y., Ku, S.K., and Kim, J.A. (2014). Serotonin regulates innate immune responses of colon epithelial cells through Nox2-derived reactive oxygen species. Free Radic. Biol. Med. *69*, 377–389. https://doi.org/10.1016/j.freeradbiomed.2014.02.003.

33. Zuo, C., Cao, H., Song, Y., Gu, Z., Huang, Y., Yang, Y., Miao, J., Zhu, L., Chen, J., Jiang, Y., and Wang, F. (2022). Nrf2: An all-rounder in depression. Redox Biol. *58*, 102522. https://doi.org/10.1016/j.redox.2022.102522.

34. Noh, J., Jung, E., Lee, J., Hyun, H., Hong, S., and Lee, D. (2019). Engineered polymeric micelles for combinational oxidation anticancer therapy through concurrent HO-1 inhibition and ROS generation. Biomacromolecules *20*, 1109–1117. https://doi.org/10.1021/acs.biomac.8b01802.

35. Xu, Z., Wang, X., Sun, W., Xu, F., Kou, H., Hu, W., Zhang, Y., Jiang, Q., Tang, J., and Xu, Y. (2023). RelB-activated GPX4 inhibits ferroptosis and confers tamoxifen resistance in breast cancer. Redox Biol. *68*, 102952. https://doi.org/10.1016/j.redox.2023.102952.

36. Wen, Y., Chen, H., Zhang, L., Wu, M., Zhang, F., Yang, D., Shen, J., and Chen, J. (2021). Glycyrrhetinic acid induces oxidative/nitrative stress and drives ferroptosis through activating NADPH oxidases and iNOS, and depriving glutathione in triple-negative breast cancer cells. Free Radic. Biol. Med. *173*, 41–51. https://doi.org/10.1016/j.freeradbiomed.2021.07.019.

37. Liu, Z., Qin, X., Zhang, B., Nong, K., Chen, W., Yang, Z., Lang, W., Liu, X., Li, L., Wang, X., et al. (2025). Proline rich-39 (PR-39) antimicrobial protein alleviated lipopolysaccharide-induced intestinal barrier dysfunction in piglets by altering intestinal flora associated bile acid metabolism and in turn regulating TGR-5/NF-κB/MLCK/MLC pathway. Int. J. Biol. Macromol. *307*, 141930. https://doi.org/10.1016/j.ijbiomac.2025.141930.

38. Srivastava, D., Schuermann, J.P., White, T.A., Krishnan, N., Sanyal, N., Hura, G.L., Tan, A., Henzl, M.T., Becker, D.F., and Tanner, J.J. (2010). Crystal structure of the bifunctional proline utilization A flavoenzyme from Bradyrhizobium japonicum. Proc. Natl. Acad. Sci. USA *107*, 2878–2883. https://doi.org/10.1073/pnas.0906101107.

39. Linder, S.J., Bernasocchi, T., Martínez-Pastor, B., Sullivan, K.D., Galbraith, M.D., Lewis, C.A., Ferrer, C.M., Boon, R., Silveira, G.G., Cho, H.M., et al. (2023). Inhibition of the proline metabolism rate-limiting enzyme P5CS allows proliferation of glutamine-restricted cancer cells. Nat. Metab. *5*, 2131–2147. https://doi.org/10.1038/s42255-023-00919-3.

40. Kang, J.E., Karla, P., Carla, D.R., Sumpton, D., Däbritz, J.H.M., Tardito, S., Boldrini, C., Hernandez-Fernaud, J.R., Athineos, D., Dhayade, S., et al. (2022). Cancer-associated fibroblasts require proline synthesis by PYCR1 for the deposition of pro-tumorigenic extracellular matrix. Nat. Metab. *4*, 693–710. https://doi.org/10.1038/s42255-022-00582-0.

41. Labun, K., Montague, T.G., Krause, M., Torres Cleuren, Y.N., Tjeldnes, H., and Valen, E. (2019). CHOPCHOP v3: expanding the CRISPR web toolbox beyond genome editing. Nucleic Acids Res. *47*, W171–W174. https://doi.org/10.1093/nar/gkz365.

42. Kim, H., and Kim, J.S. (2014). A guide to genome engineering with programmable nucleases. Nat. Rev. Genet. *15*, 321–334. https://doi.org/10.1038/nrg3686.

43. Brinkman, E.K., Kousholt, A.N., Harmsen, T., Leemans, C., Chen, T., Jonkers, J., and van Steensel, B. (2018). Easy quantification of template-directed CRISPR/Cas9 editing. Nucleic Acids Res. *46*, e58. https://doi.org/10.1093/nar/gky164.

## STAR★METHODS

### KEY RESOURCES TABLE

| REAGENT or RESOURCE | SOURCE | IDENTIFIER |
|---|---|---|
| **Antibodies** | | |
| GAPDH antibody | Baode Biotech | Cat#BS00243-T |
| gp91$^{phox}$ antibody | Baode Biotech | Cat#BS79741 |
| NRF2 antibody | Baode Biotech | Cat#BS1258 |
| Heme oxygenase-1 antibody | Abcam | Cat# ab13248;RRID:AB_2118663 |
| Cas9 antibody | Abcam | Cat# ab50014; RRID:AB_2068610 |
| PHD antibody | Youpin Biotechnology | Cat#YP-mAb-18357 |
| PRODH antibody | Youpin Biotechnology | Cat#YP-mAb-18497 |
| P5CS antibody | Youpin Biotechnology | Cat#YP-mAb-18293 |
| PYCR1 antibody | Youpin Biotechnology | Cat#YP-mAb-17244 |
| ZO-1 antibody | Nature Biosciences | Cat#A96695 |
| Claudin-1 antibody | Nature Biosciences | Cat#A23098 |
| **Chemicals, peptides, and recombinant proteins** | | |
| Hyaluronic acid | Macklin | Cat#H909936 |
| Magnesium chloride | Macklin | Cat#M813763 |
| Chitosan | Macklin | Cat#C804729 |
| Kanamycin | Genview | Cat#AK177 |
| Chloramphenicol | Genview | Cat#AC060 |
| IPTG | Genview | Cat#C1175 |
| Total RNA Extraction Reagent | Novizan Biotechnology | Cat#R401-01 |
| DiR (DiIC18(7)) | Aladdin | N/A |
| DCFH-DA probe | Solarbio | Cat#ID31309 |
| Pre-stained protein markers | Sainor Biotech | Cat#SB-26616 |
| Pre-stained protein markers | Sainor Biotech | Cat#SB-26619 |
| T7 Endonuclease I (T7E1) | Sangon Biotech | Cat#B610169 |
| **Critical commercial assays** | | |
| CCK-8 assay kit | Solarbio | Cat#CA1210 |
| Cell Viability and Cytotoxicity Assay Kit | Solarbio | Cat#CA1633 |
| BCA assay kit | Sainor Biotechnology | Cat#SB-WB013 |
| **Deposited data** | | |
| Raw and analyzed data | This paper | GEO: GSE214695 |
| **Experimental models: Cell lines** | | |
| Caco-2 cells | Human Fenghui Biotechnology Co., Ltd. | N/A |
| Raw264.7 cells | Laboratory stock | N/A |
| A549 cells | Laboratory stock | N/A |
| **Experimental models: Organisms/strains** | | |
| Mouse: C57BL/6 | Liaoning Changsheng Biotechnology | N/A |
| **Recombinant DNA** | | |
| pET-22a-Cas9-kana | Genewiz, Inc. | N/A |
| pUC57-sgRNA-Chl | Genewiz, Inc. | N/A |
| **Software and algorithms** | | |
| ChopChop software | Labun et al.[41] | https://chopchop.cbu.uib.no/ |
| Cas-OFFinder | Kim et al.[42] | http://www.rgenome.net/cas-offinder/ |
| TIDE | Brinkman et al.[43] | https://tide.nki.nl/ |

## EXPERIMENTAL MODEL AND STUDY PARTICIPANT DETAILS

### Mice samples

Male SPF wild-type C57BL/6 mice (6–8 weeks, 18–20 g, Liaoning Changsheng Biotechnology) were housed under SPF conditions with a 12 h light/dark cycle and *ad libitum* access to food and water at room temperature. All procedures were approved by the Animal Ethics Committee of Liaoning University (LNU-2022-091,301) and followed institutional guidelines. Sex of mice did not influence the experimental outcomes. Mice were randomly divided into experiment groups ($n$ = 5/group). The control group received normal drinking water, while the other six groups received 2.5% (w/v) dextran sulfate sodium (DSS) solution for five days. Starting from the fifth day, treatment groups (EcN-RNP, EcN-RNP+IPTG, EcN-RNP@HCMg, EcN-RNP@HCMg+IPTG, and EcN-RNP@HCCa+IPTG) were gavage with 200 μL samples every other day, and all mice were euthanized on day 10. IPTG was mixed with the samples at a final concentration of 1 mM.

### Microbe strains

Wild-type *Escherichia coli* 1917(EcN) was cultured in Luria-Bertani medium (10 g/L tryptone, 5 g/L yeast extract, 10 g/L NaCl) for experiments.

### Cell lines

Wild-type Caco-2 (human colorectal adenocarcinoma) cells were purchased from Human Fenghui Biotechnology Co., Ltd. Wild-type Raw264.7 (Mouse Monocytic Macrophage Leukemia Cell Line) and Wild-type A549 (Human Non-Small Cell Lung Cancer Cell Line) cells were stored in the laboratory. All A549, Caco-2 and RAW264.7 cells were cultured in a constant-temperature incubator at 37°C with 5% $CO_2$. A549 cells were maintained in F-12 K medium supplemented with 10% fetal bovine serum (FBS) and penicillin–streptomycin. Caco-2 and RAW264.7 cells were cultured in high-glucose DMEM containing 10% FBS and penicillin–streptomycin. All cell lines were authenticated via short tandem repeat (STR) profiling to confirm their identity before experiments. Mycoplasma contamination testing was routinely performed using a PCR-based detection method, and all cells were confirmed mycoplasma-negative throughout the study. The reagents and kits for biological assays are listed in the key resources table.

## METHOD DETAILS

### Materials

Caco-2 (human colorectal adenocarcinoma) cells were purchased from Human Fenghui Biotechnology Co., Ltd. Raw264.7 and A549 cells were stored in the laboratory. The plasmid pET-22a-Cas9-kana and pUC57-sgRNA-Chl was prepared by Genewiz, Inc. Hyaluronic acid (H909936), magnesium chloride (M813763), and chitosan (C804729) were purchased from Macklin. Kanamycin (AK177). Chloramphenicol (AC060) and IPTG (C1175) were all purchased from Genview. Total RNA Extraction Reagent (R401-01) was purchased from Novizan Biotechnology. DiR (DiIC18(7)) was obtained from Aladdin. DCFH-DA probe (ID31309), CCK-8 assay kit (CA1210) and Cell Viability and Cytotoxicity Assay Kit (CA1633) were acquired from Solarbio. Pre-stained protein markers (SB-26616/SB-26619) and BCA assay kit (SB-WB013) were purchased from Sainor Biotech. GAPDH antibody (BS00243-T), gp91$^{phox}$ antibody (BS79741), and NRF2 antibody (BS1258) were bought from Baode Biotech. T7 Endonuclease I(T7E1, B610169) was bought from Sangon Biotech. Heme oxygenase-1 antibody (ab68477) and Cas9 antibody (AB315191) were bought from Abcam. PHD antibody (YP-mAb-18357), PRODH antibody (YP-mAb-18497), P5CS antibody (YP-mAb-18293), and PYCR1 antibody (YP-mAb-17244) were purchased from Youpin Biotechnology. ZO-1 (A96695) and Claudin-1 (A23098) antibody were purchased from Nature Biosciences. All the primer sequences can be found in Table S1.

### Single-cell transcriptomic analysis

Single-cell transcriptomic data included 18 samples (6 healthy controls, 6 UC patients, and 6 CD patients) from GSE214695 dataset (25), data preprocessing, quality control, batch correction, cell clustering and annotation were performed using the Seurat framework.

### Engineering EcN expressing CRISPR/Cas9

A single colony containing the Cas9-expressing plasmid (pET-22a-Cas9) was cultured in LB medium to an $OD_{600}$ of approximately 0.5. Competent cells were prepared by chilling, centrifuging, and resuspending in ice-cold 0.05 mol/L $CaCl_2$ solution and a glycerol/$CaCl_2$ solution. These competent cells were transformed with pUC57-sgRNA-Chl plasmid via a 30-min ice incubation, a 60-s heat shock at 42°C, and immediate cooling on ice. Following a 45-min recovery in LB medium, the mixture was plated on agar and incubated at 37°C.

### Preparation of EcN-RNP hydrogel and measurement of zeta potential, encapsulation efficiency, growth curve

The EcN sediment was mixed with 0.25 M $MgCl_2$ solution and 0.5% (m/v) chitosan prepared in 0.1 M HCl, then 5% (w/v) hyaluronic acid was layered on top. The three components were mixed in a 1:1:1 volume ratio and allowed to stand overnight for forming hydrogel. Then IPTG was added with a final concentration of 1 mM. 1 mL $MgCl_2$, chitosan, hyaluronic acid, EcN+$MgCl_2$, EcN+$MgCl_2$+

hyaluronic acid, and EcN+$MgCl_2$+ hyaluronic acid was prepared separately. Their zeta potentials were measured using a zeta potential analyzer. The hydrogel was immersed in PBS and taken out at regular time intervals, blotted dry with filter paper and weighed to calculate its swelling ratio. Injectability was evaluated using a 1 mL syringe.$10^5$, $10^6$, $10^7$, $10^8$, and $10^9$ CFU/mL EcN was encapsulated with $MgCl_2$, hyaluronic acid, and chitosan, respectively. After dilution, the samples were plated and incubated overnight at 37°C for colonies counting. As for growth curve, the $OD_{600}$ values of the EcN-RNP culture solution and the EcN-RNP@HCMg culture solution were measured at 0, 0.5, 1, 1.5, 2, 4, 6, 8, 10, 24, 26 and 28 h, respectively.

### Isopropyl β-D-1-thiogalactopyranoside (IPTG)-induced Cas9 expression assay

EcN-RNP, EcN-RNP + IPTG, EcN-RNP@HCMg and EcN-RNP@HCMg+ IPTG were incubated overnight in a shaker at 37°C and 220 rpm. When the $OD_{600}$ of the EcN-RNP and EcN-RNP@HCMg culture reaches 0.6, IPTG was added to a final concentration of 1 mM. SDS-PAGE and western blot were used to verify IPTG-induced Cas9 expressions. Samples were added in the gel based on the BCA assay results, electrophoresis was initiated at a voltage of 80 V and then at a voltage of 150 V. The gel was stained with Coomassie Brilliant Blue for 2 h, followed by overnight destaining.

Electrophoresis was performed as described above. The transfer was performed at low temperature using a constant voltage of 100 V for 1 h. Then, the membrane was blocked with 5% non-fat milk prepared in Tris-Buffered Saline with Tween 20 (TBST) at room temperature for 1 h. The membrane was washed three times with TBST. The membrane was then incubated with the primary antibody (Cas9, goat anti-rabbit, 1:1000; GAPDH, 1:5000, goat anti-mouse) at 4°C overnight. The following day, the membrane was washed three times with TBST and then incubated with secondary antibody (goat anti-mouse IgG-HRP) at room temperature for 2 h. Subsequently, the membrane was washed three times with TBST and carried out signal shooting.

### Detection of intestinal stability and retention time

EcN-RNP and EcN-RNP@HCMg were prepared at a concentration of $3 \times 10^9$ CFU/mL. The samples were incubated in simulated gastric fluid (SGF, 10 g/L pepsin, 16.4 mL/L dilute HCl) at 37°C with gentle shaking at 100 rpm for 2 h and simulated intestinal fluid (SIF, 6.8 g/L $KH_2PO_4$, 77 mL/L 0.2 M NaOH, 10 g/L pancreatin, pH 6.8) at 37°C with gentle shaking at 100 rpm for 4 h, respectively. After incubation, samples were added 1 mL LB medium and incubated at 37°C with shaking at 100 rpm for 30 min. Then the culture was spread onto agar plates and incubated overnight at 37°C.

Colon segments were harvested from overnight-fasted C57BL/6 mice and cut into 1 cm × 4 cm pieces. EcN-RNP and EcN-RNP@HCMg were prepared at a concentration of $3 \times 10^9$ CFU/mL and the prepared colon tissues were covered with them. The colon tissues were mounted on a 45° platform and 1 mL PBS was added, shaking at 60 rpm for 15 min. The above procedure was repeated three times. The eluate was collected and plated onto agar plates. After incubating overnight at 37°C, the colonies were counted.

The EcN cell membrane was labeled with DiR [DiIC18(7)], EcN-RNP and EcN-RNP@HCMg were prepared at a concentration of $10^9$ CFU/mL. The samples were orally administered to C57BL/6 mice via gavage, and intestinal fluorescence was periodically imaged using an X-ray imaging system.

EcN-EGFP expressing green fluorescent protein was selected as the model strain for cellular uptake assays, and EcN-EGFP@HCMg was prepared according to the above method. The sample was added to the upper chamber of the Transwell model, while different cell types (A549, Caco-2 and RAW264.7 cells) were seeded in the lower chamber. All A549, Caco-2 and RAW264.7 cells were cultured in a constant-temperature incubator at 37°C with 5% $CO_2$. A549 cells were maintained in F-12 K medium supplemented with 10% fetal bovine serum (FBS) and penicillin–streptomycin. Caco-2 and RAW264.7 cells were cultured in high-glucose DMEM containing 10% FBS and penicillin–streptomycin. After overnight incubation, the cells were rinsed with PBS and lysed with RIPA lysis buffer. Detection was performed using a microplate reader at an excitation wavelength of 480 nm and an emission wavelength of 520 nm.

### Detection of intracellular ROS levels using flow cytometry and fluorescence microscopy

EcN-RNP and EcN-RNP@HCMg were prepared at a concentration of $3 \times 10^9$ CFU/mL, and IPTG was added to the cells together with the samples at a final concentration of 1 mM, this dosage was used in all subsequent cellular experiments unless otherwise specified. A Transwell co-culture model with a 0.4 μm pore size was used, in which samples was added to the upper chamber and target cells were seeded in the lower chamber. The Transwell co-culture model was used in all the cell experiments unless otherwise specified. The cells were washed twice with PBS after co-culturing with samples in a 6-well plate for 3 h. The DCFH-DA probe was diluted 1:1000 in serum-free medium, and 1 mL the solution was added to each well. The cells were then incubated in a $CO_2$ incubator for 30 min and finally observed under a fluorescence microscope. The samples were prepared as described above, and 0.5 mL trypsin was added to each well for digesting the cells. The free cells were analyzed by flow cytometry.

### Live/dead staining and cell counting Kit-8 detection

The cells were washed twice with PBS after co-culturing with samples in a 6-well plate for 3 h. A working solution was prepared by mixing Calcein AM, PI and assay buffer at a ratio of 1:1:1000. The samples were added with 1 mL working solution each well and incubated at 37°C in the dark for 30 min. Then the samples were observed by fluorescence microscopy. Cells were co-cultured with samples in a 96-well plate for 3 h, 10 μL Cell Counting Kit-8 (CCK-8) reagent was added to each well. The plate was incubated in a cell culture incubator for 2 h, and absorbances of the plate were measured at 562 nm.

### RT-PCR detection of inflammatory factors

Cells co-cultured with samples were lysed with 1 mL RNA lysis buffer. After adding 200 μL chloroform, the mixture was vigorously vortexed (10 times) and incubated at room temperature for 5 min. Following centrifugation (12,000 rpm, 4°C, 15 min), the upper aqueous phase was transferred to a new tube, an equal volume of isopropanol was added, and the samples were centrifuged (12,000 rpm, 4°C, 10 min). The supernatant was discarded, and the RNA pellet was washed with pre-chilled 75% ethanol, air-dried for 5–10 min, and dissolved in 30–40 μL water. For reverse transcription, 1 μg RNA and 4 μL 5× HiScript III qRT Super Mix were mixed, and the total volume were adjusted to 20 μL with RNase-free dd$H_2O$. cDNA was synthesized using the program: 37°C for 15 min, 85°C for 5 s. The qPCR reaction mixture comprised 1 μL cDNA, 10 μL 2×RealStar Universal SYBR qPCR Mix, 0.5 μL each of forward and reverse primers, and 8 μL sterile water. The mRNA expression levels were measured using a Thermo Fisher 7500 real-time quantitative PCR instrument.

### Western blotting assay

Cells were co-cultured with samples for 3 h and total protein was extracted. SDS-PAGE, membrane transfer and blocking were performed as described in Section 2.5. The primary antibody against GAPDH was diluted at 1:5000, and the secondary antibody was goat anti-mouse. Primary antibodies against $gp91^{phox}$, HO-1, NRF2, and GPX4 were all diluted at 1:1000, and the corresponding secondary antibody was goat anti-rabbit.

### EcN-RNP hydrogel induced transformation of proline-to-glutamic acid metabolism

Caco-2 cells (human colorectal adenocarcinoma cell) were added 2 μL lipopolysaccharide (LPS,1 μg/mL) and co-cultured with EcN-RNP@HCMg+IPTG for three hours. The cells were collected by treating with trypsin and centrifugation at 1500 rpm for 5 min. The cells were rapidly frozen in liquid nitrogen for 15 min and immediately stored at −80°C. The cells were sent to Yixi Technology (Guangzhou) Co., Ltd. for analysis using an Agilent 1100 liquid chromatography system.

WB were performed as described in Section 2.5. The primary antibody against β-actin was diluted at 1:5000, and the secondary antibody was goat anti-mouse. Primary antibodies against PYCR1, P5CS, P5CDH, PHD, and PRODH were all diluted at 1:1000, and the corresponding secondary antibody was goat anti-rabbit.

### Animal experiments

Male SPF healthy C57BL/6 mice (6–8 weeks, 18–20 g, Liaoning Changsheng Biotechnology) were housed under SPF conditions with a 12 h light/dark cycle and *ad libitum* access to food and water at room temperature. All procedures were approved by the Animal Ethics Committee of Liaoning University (LNU-2022-091,301) and followed institutional guidelines. Mice were randomly divided into seven groups ($n$ = 5/group). The control group received normal drinking water, while the other six groups received 2.5% (w/v) dextran sulfate sodium (DSS) solution for five days. Starting from the fifth day, treatment groups (EcN-RNP, EcN-RNP+IPTG, EcN-RNP@HCMg, EcN-RNP@HCMg+IPTG, and EcN-RNP@HCCa+IPTG) were gavage with 200 μL samples every other day, and all mice were euthanized on day 10. IPTG was mixed with the samples at a final concentration of 1 mM. The heart, liver, spleen, lungs, kidneys and colon from each group were collected, photographed and weighed. Colon segments (1–2 cm) and major organs were excised under aseptic conditions, fixed in 1 mL tissue fixative and sent to Saver Biotechnology Co., Ltd. for H&E, masson's trichrome and immunohistochemical staining. Primary antibodies against ZO-1 and Claudin-1 were all diluted at 1:1000, and the corresponding secondary antibody was goat anti-rabbit. Additionally, colon tissues from three mice were collected and immediately frozen in liquid nitrogen, then shipped on dry ice to Saver Company for 16 S rRNA sequencing.

### Detection of gene editing efficiency

sgRNA sequences were designed using the ChopChop software,[41] with gene editing efficiency and off-target sites predicted simultaneously. The Cas-OFFinder tool[42] was additionally used for off-target prediction. The PCR reaction system consisted of 1 μL template DNA, 10 μL 2× PCR Master Mix, 0.5 μL forward primer, 0.5 μL reverse primer, and 8 μL sterile water. PCR amplification was performed on a thermal cycler. PCR products were sent to Genewiz Company for Sanger sequencing, and the sequencing results were subsequently analyzed via the TIDE algorithm.[43] The T7E1 assay was carried out using a kit.

## QUANTIFICATION AND STATISTICAL ANALYSIS

Data was presented as mean ± SD. Statistical analyses were performed using Student's t-tests or one-way ANOVA in GraphPad Prism (Version 9). Statistical significance was defined as $p < 0.05$ and ns was defined as no significant difference.

**Cell Reports Medicine, Volume *7***

## Supplemental information

# CRISPR-Cas9-producing probiotic bacteria for editing NOX2/gp91$^{phox}$ and treating inflammatory bowel disease

**Chen Zhang, Tianyu Li, Huoye Hao, Gang Wang, Yuzhe Zhang, Yue Huang, Lili Chen, Jin Wang, Xiang-Jiao Yang, and Xiuli Bi**

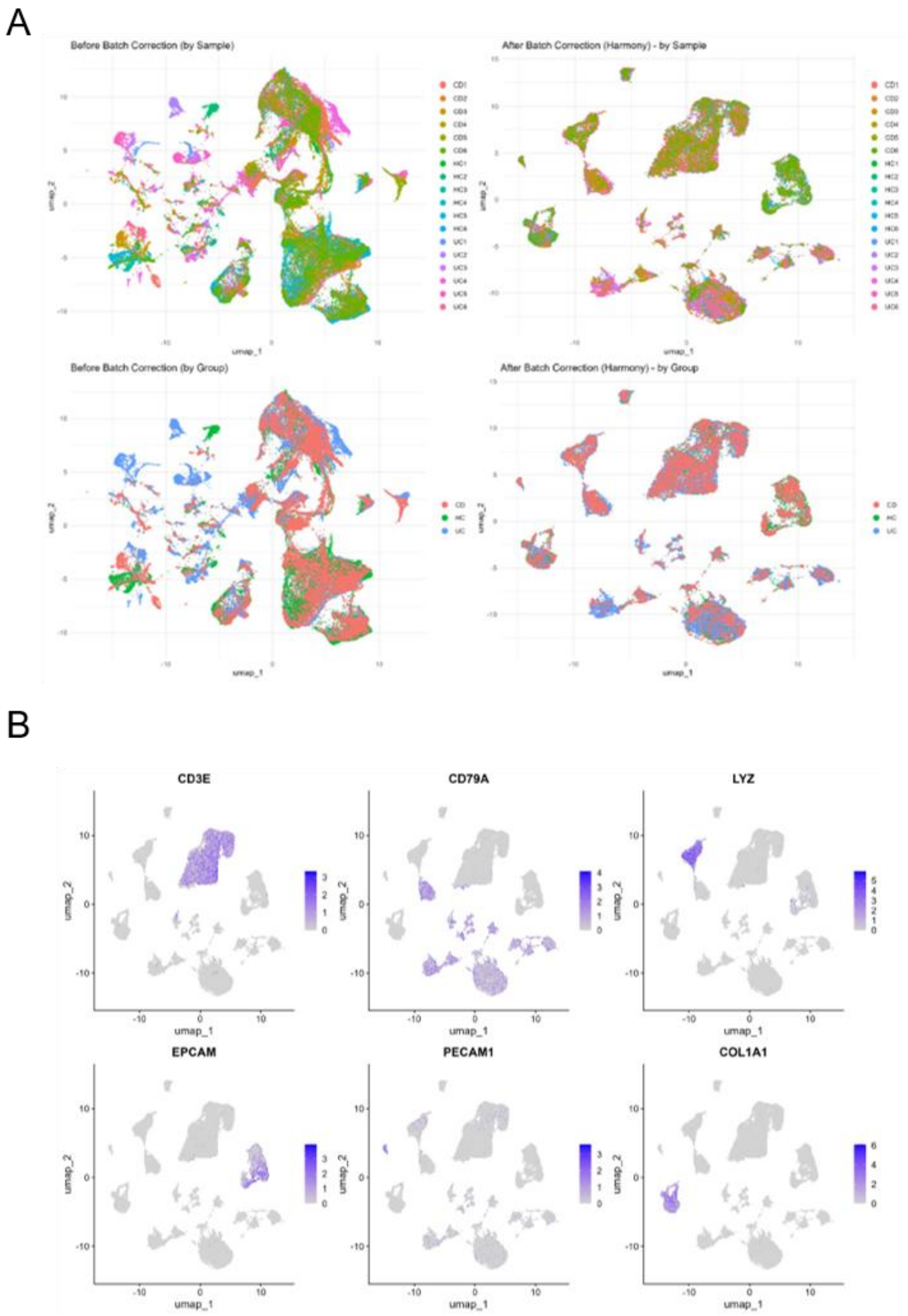


**Figure S1. Clustering and annotation of cells, Related to Figure 1.**

A) Clustering and annotation of cells before and after batch correction. The results show that cells clustered primarily by sample, exhibiting a clear batch effect. Cells were more evenly intermixed after correction, which preserved the biological differences between disease groups. B) Identification of marker genes. The results showed that marker genes were specifically expressed in their corresponding cell clusters, validating the accuracy of cell type annotations.

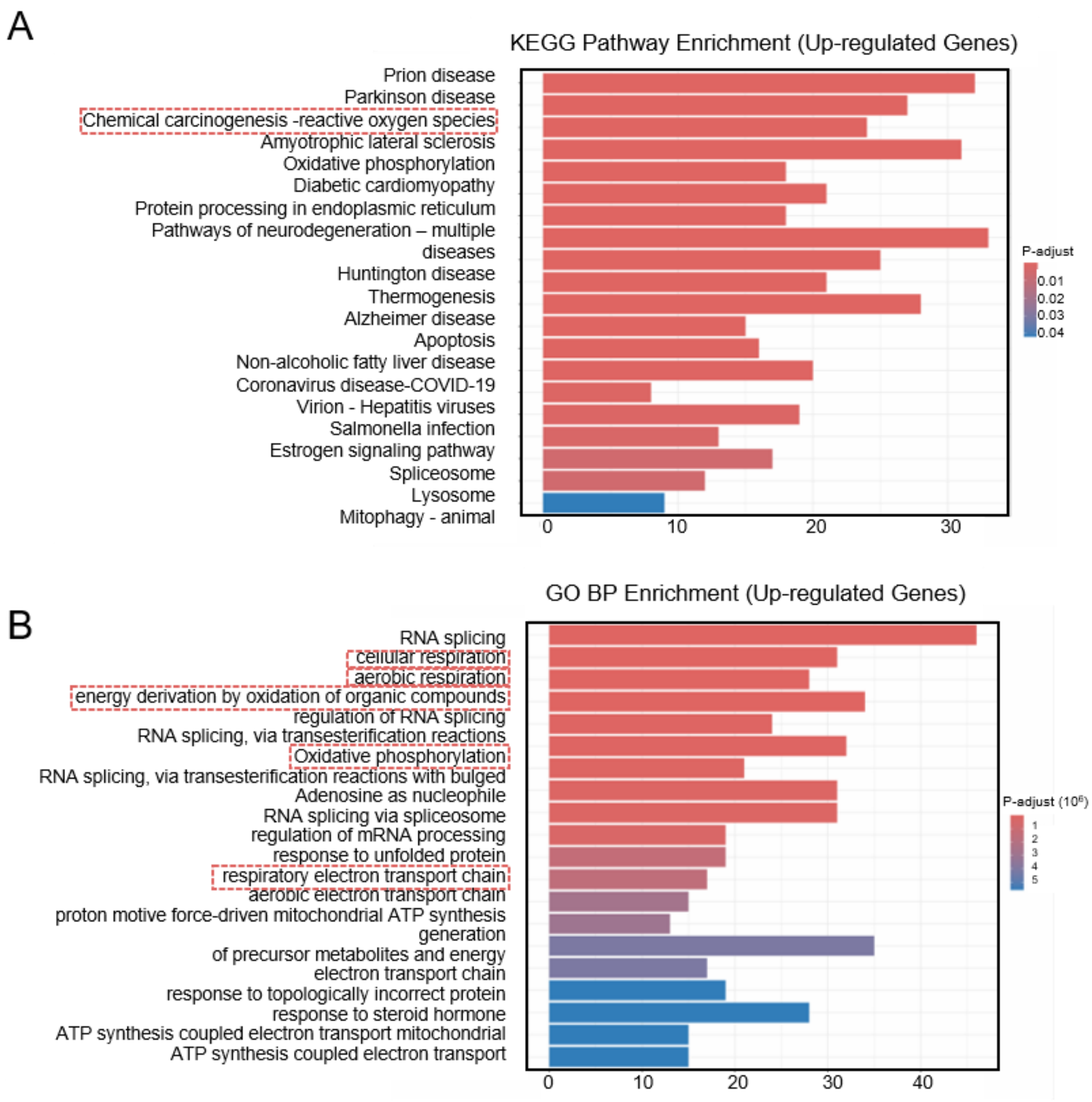


**Figure S2. Differentially expressed genes (DEGs) enriched in oxidative stress pathways, Related to Figure 1.**

(A) KEGG and (B) GO pathway enrichment analyses. Differentially expressed genes (DEGs) enriched in oxidative stress, inflammatory response and ROS metabolism pathways.

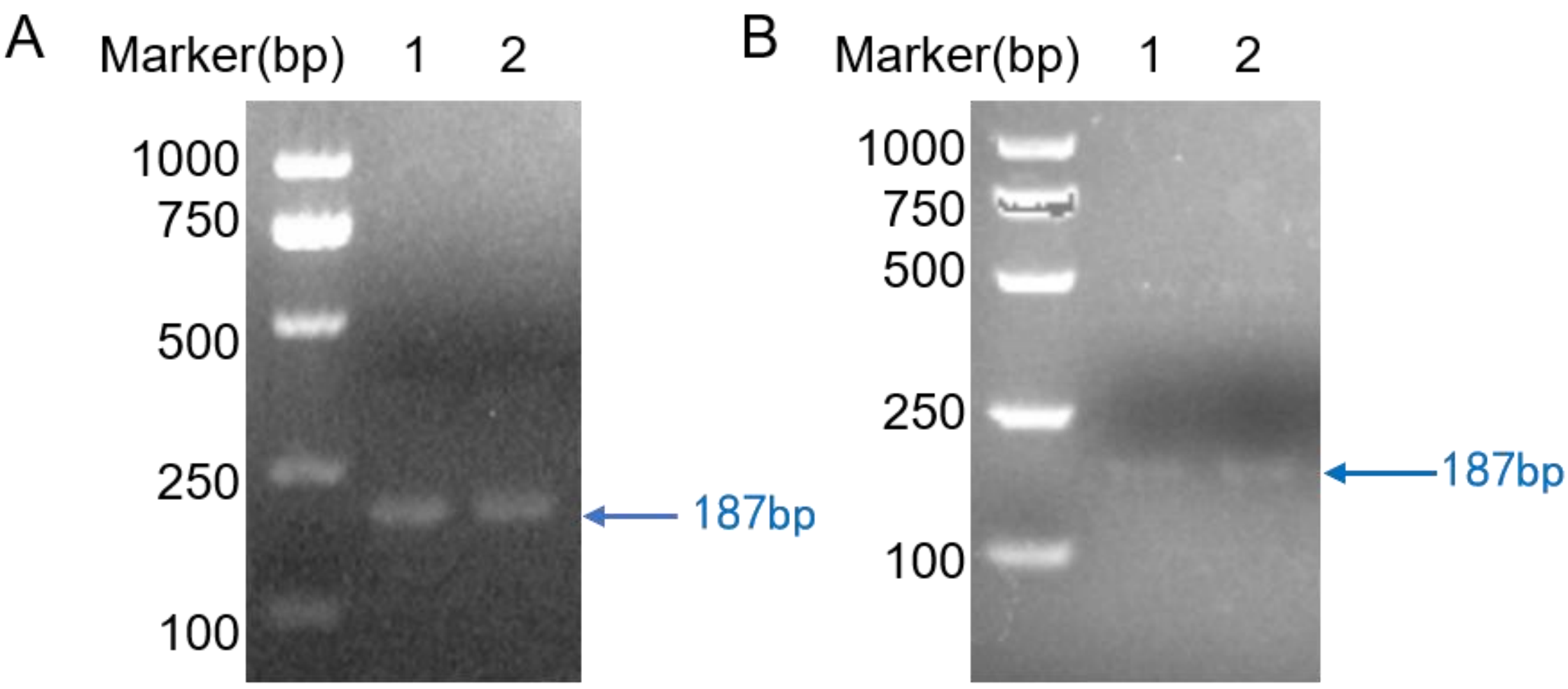


**Figure S3. Verification of the gp91$^{phox}$ gene sequence in cultured cells and colon tissues, Related to Figure 1.**

A) Verification of the gp91$^{phox}$ gene sequence in cultured cells. lane 1, Caco-2 cells, lane 2, RAW264.7 cells. B) Verification of the gp91$^{phox}$ gene sequence from colon tissues, lanes 1 and 2 represent repetitive testing.

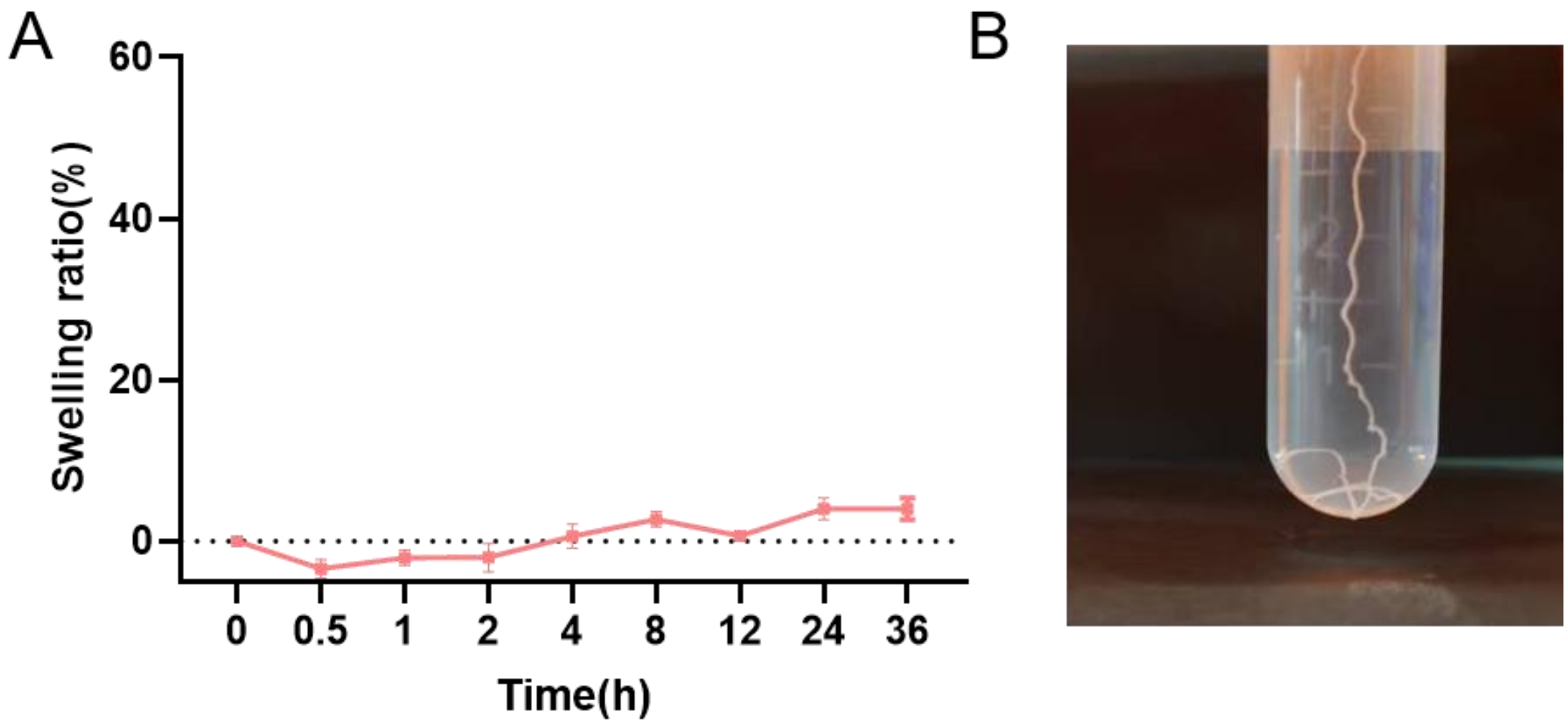


**Figure S4. Swelling ratio and injectability of EcN-RNP@HCMg hydrogel, Related to Figure 1.**

Swelling ratio A) and injectability B) of EcN-RNP@HCMg hydrogel. EcN-RNP@HCMg exhibits a maximum swelling ratio and injectability, these characteristics effectively reduce the risk of intestinal embolism and laceration.

A

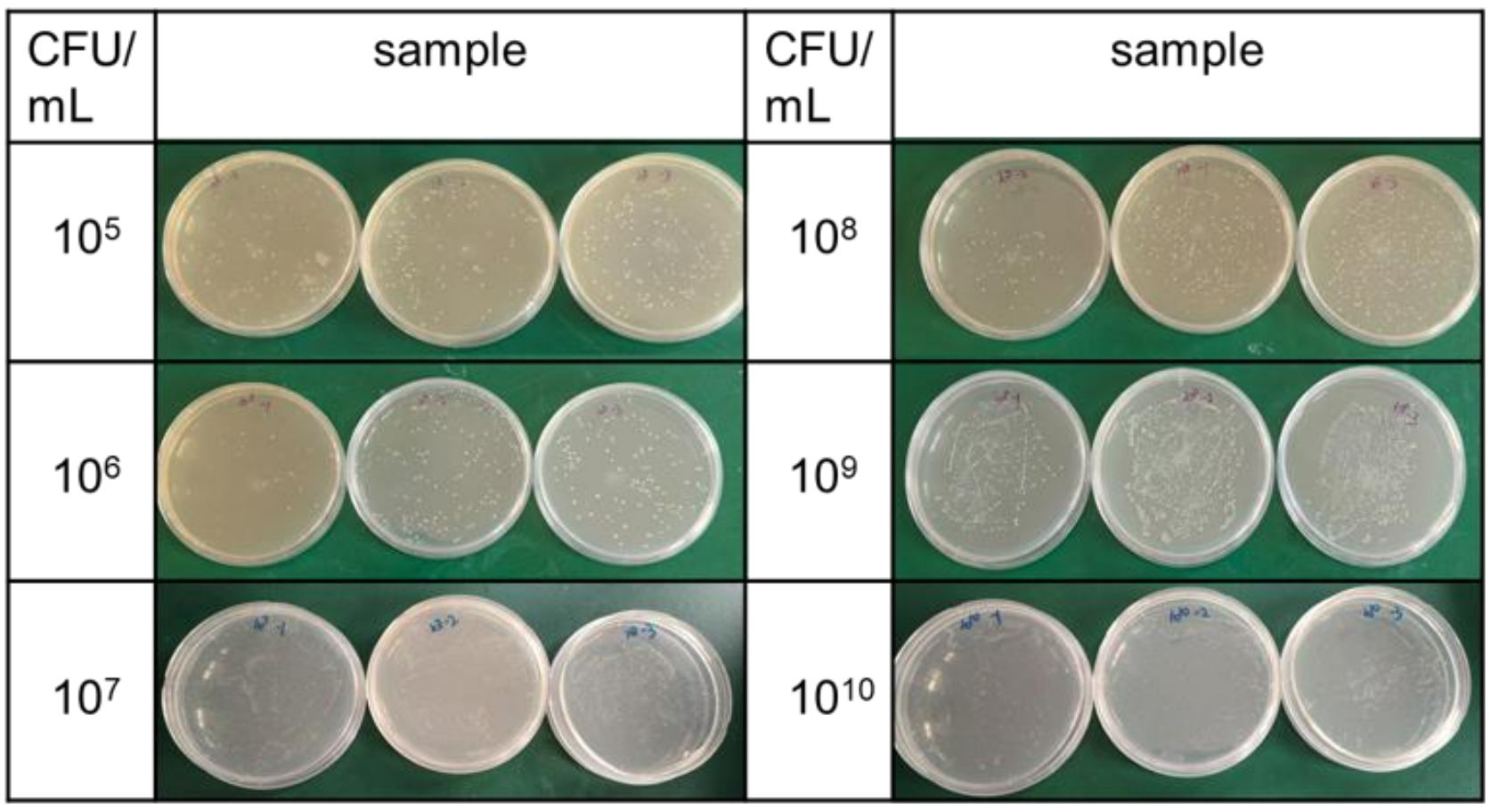


B

| bacteria (CFU/mL) | sample 1 | sample 2 | sample 3 | encapsulation rate% |
|---|---|---|---|---|
| $10^5$ | 78 | 95 | 140 | 1.04% |
| $10^6$ | 11 | 176 | 119 | 0.92% |
| $10^7$ | 110 | 100 | 90 | 0.93% |
| $10^8$ | 59 | 295 | 423 | 2.59% |
| $10^9$ | 1100 | 800 | 750 | 8.83% |
| $10^{10}$ | 90 | 60 | 50 | 0.67% |

**Figure S5. Encapsulation analysis of EcN-RNP@HCMg hydrogel, Related to Figure 1.**

A) Images of colonies showing bacterial encapsulation analysis. B) Encapsulation efficiency statistics. The results show that the maximum encapsulation efficiency of 8.83% by HA, CS and $MgCl_2$ was achieved at a bacterial concentration of $10^9$ CFU/mL.

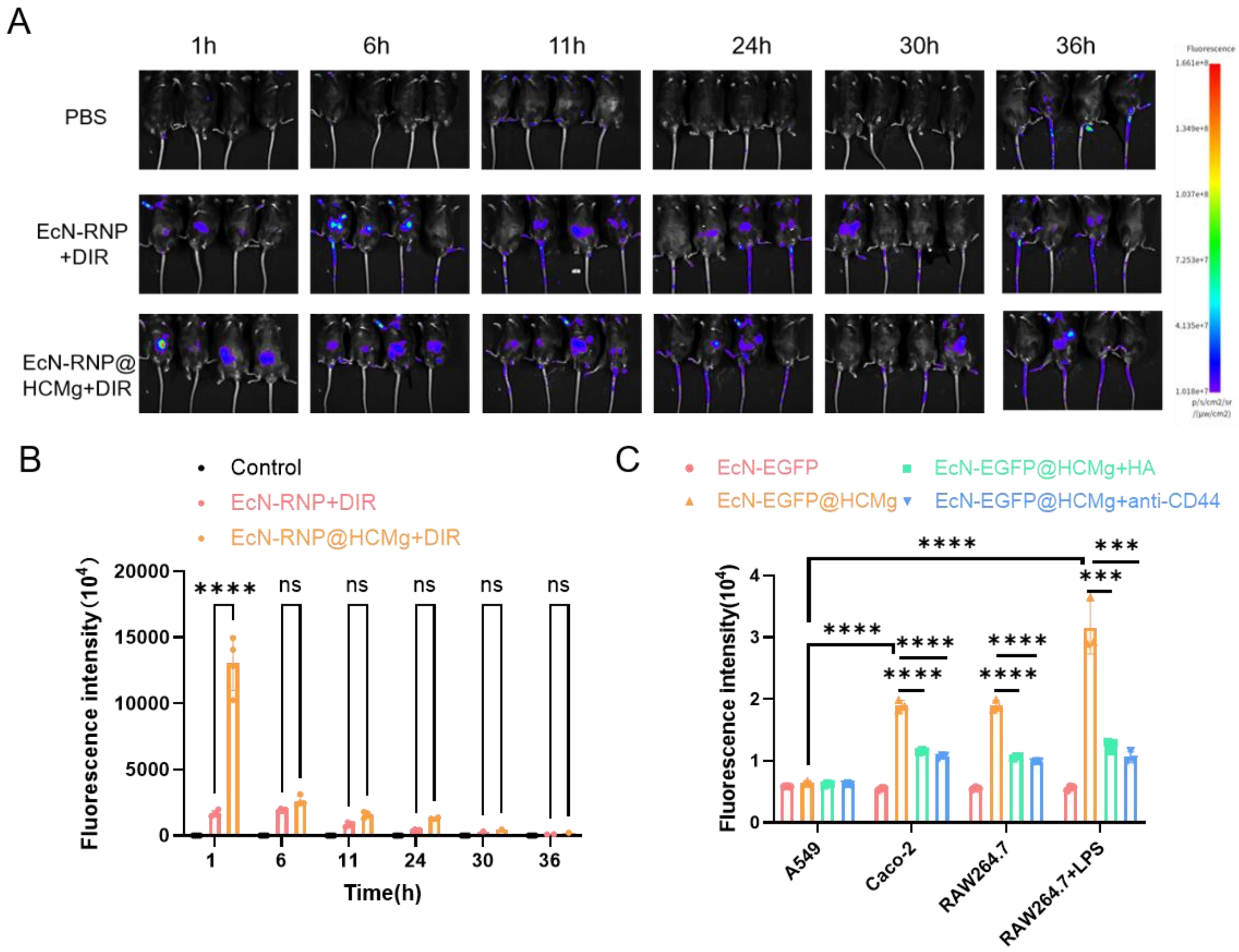


**Figure S6. IVIS and cell uptake analysis of HA Targeting Properties, Related to Figure 2.**

Fluorescence images of IBD mice following oral administration (A and B). C) Statistics of the uptake of EcN-EGFP@HCMg by different cells. Data are presented as mean ± SD, ns: no significance, *p < 0.05, **p < 0.01 and ***p < 0.001 calculated using Student's test or one-way ANOVA.

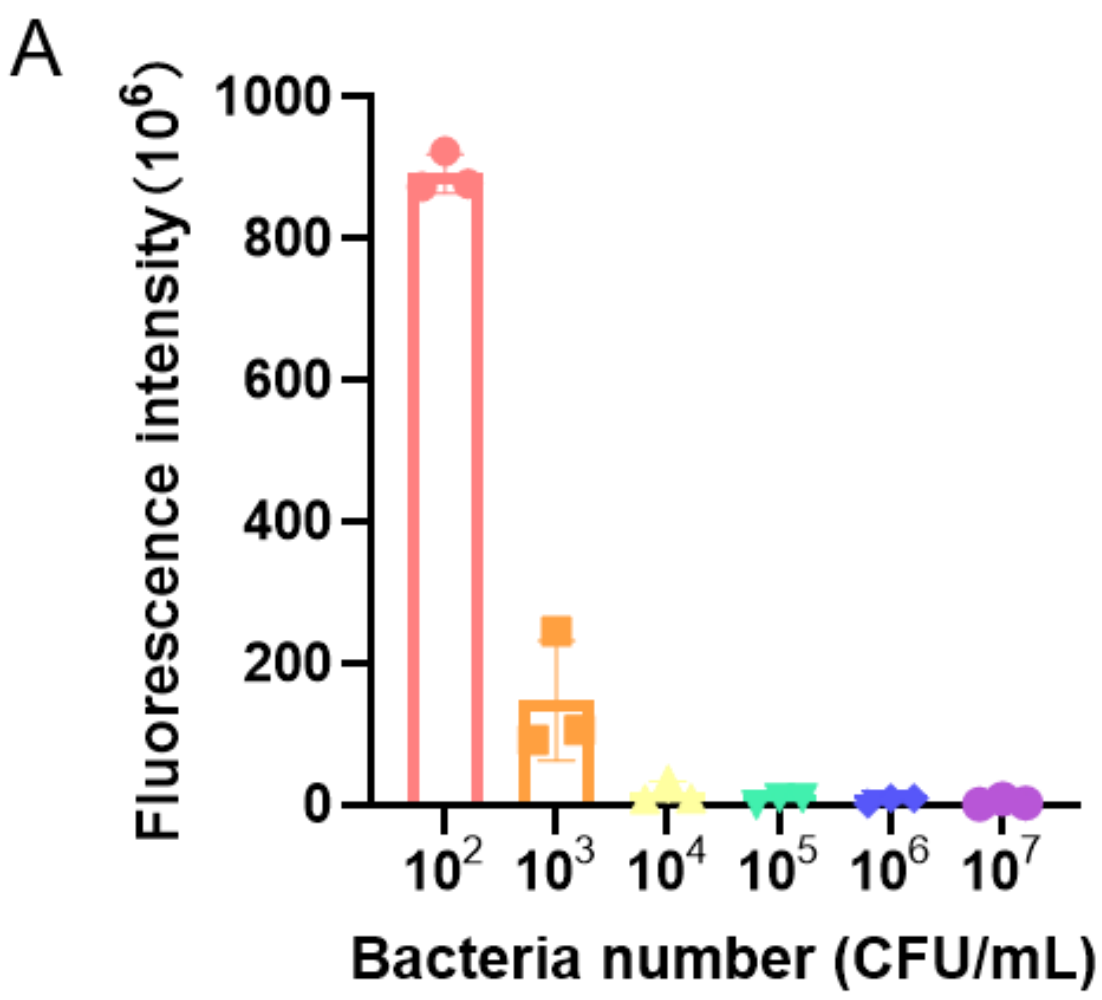


**Figure S7. Intracellular ROS levels Caco-2 cells, Related to Figure 3.**

Intracellular ROS levels Caco-2 cells were treated by EcN-RNP@HCMg of different EcN concentrations (A) and different times (B). Cells were co-cultured with EcN-RNP@HCMg at concentrations of $10^2$, $10^3$, $10^4$, $10^5$, $10^6$, and $10^7$ CFU/mL, and the results showed that higher bacterial concentrations led to lower ROS levels. Moreover, the effects of EcN-RNP@HCMg on reducing ROS levels gradually enhanced as the co-culture duration increased. Data are presented as mean ± SD, ns: no significance, *$p < 0.05$, **$p < 0.01$ and ***$p < 0.001$ calculated using Student's test or one-way ANOVA.

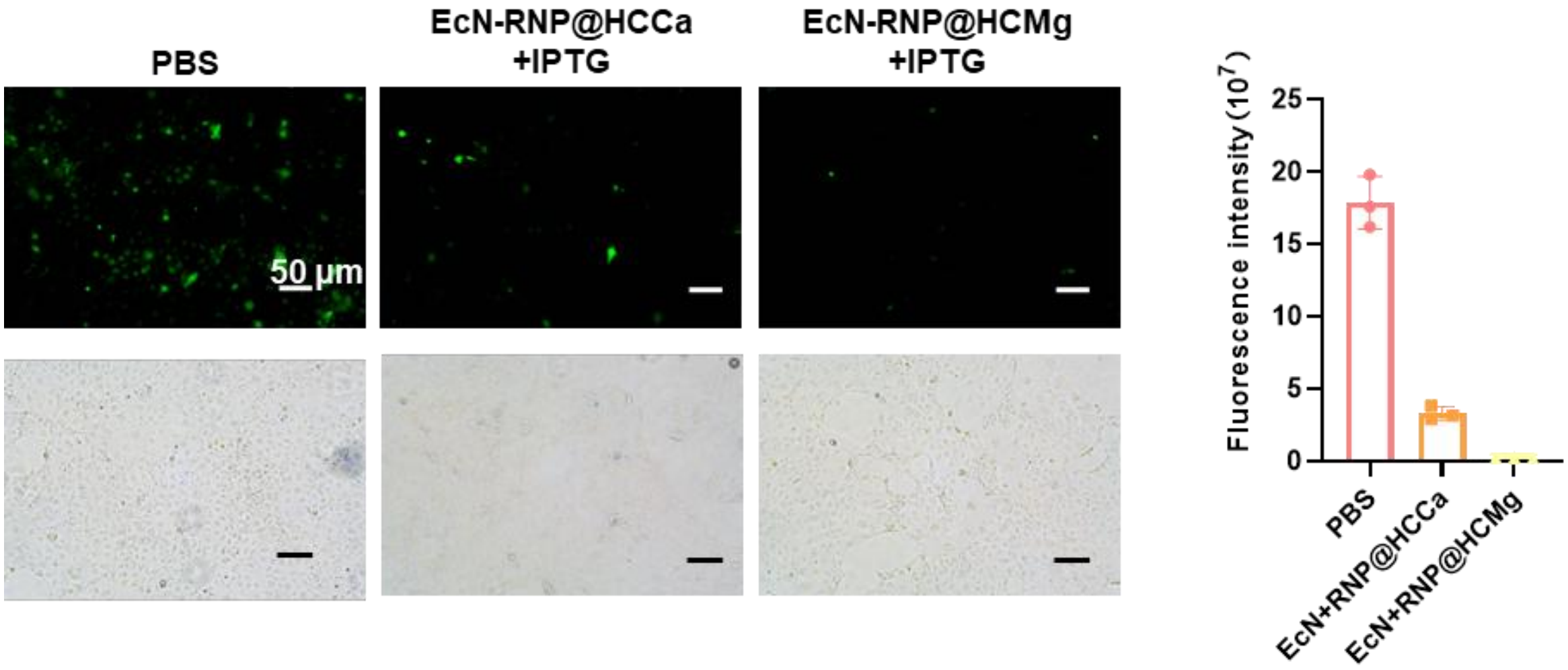


**Figure S8. Effects of $Mg^{2+}$ and $Ca^{2+}$ were compared by detecting the intracellular ROS levels, Related to Figure 3.**

Effects of EcN-RNP@HCCa+IPTG and EcN-RNP@HCMg+IPTG on reducing ROS levels in Caco-2 cells following $H_2O_2$ treatment. The results show that both EcN-RNP@HCCa+IPTG and EcN-RNP@HCMg+IPTG can reduce ROS levels in cells, with EcN-RNP@HCMg+IPTG exhibiting a superior effect. Data are presented as mean ± SD, ns: no significance, *p < 0.05, **p < 0.01 and ***p < 0.001 calculated using Student's test or one-way ANOVA.

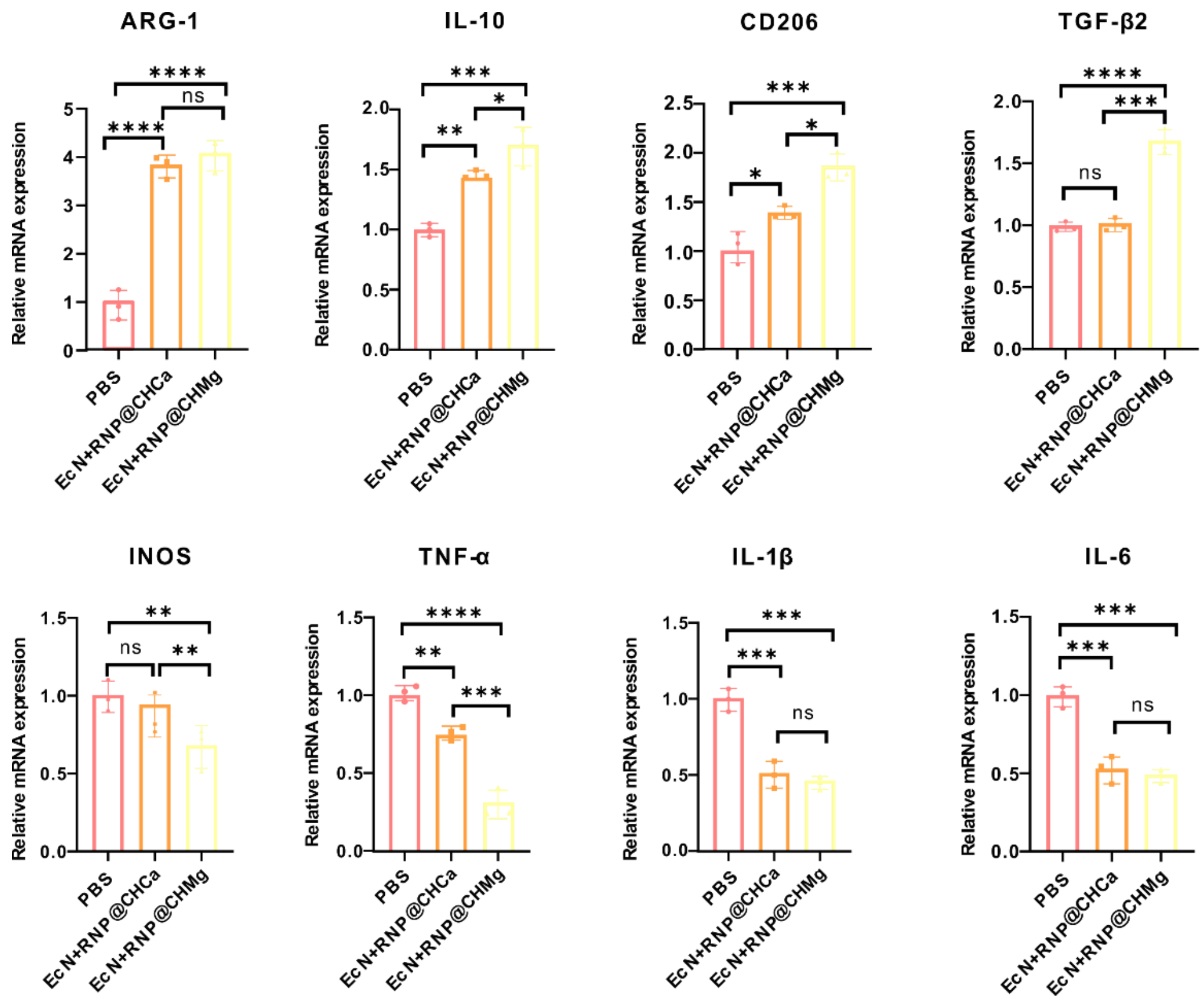


**Figure S9. Effects of $Mg^{2+}$ and $Ca^{2+}$ were compared by detecting the expression of inflammatory factors, Related to Figure 3.**

Effects of EcN-RNP@HCCa + IPTG and EcN-RNP@HCMg + IPTG on mRNA expression of inflammatory cytokines in LPS-treated RAW264.7 cells. Data are presented as mean ± SD, ns: no significance, *p < 0.05, **p < 0.01 and ***p < 0.001 calculated using Student's test or one-way ANOVA.

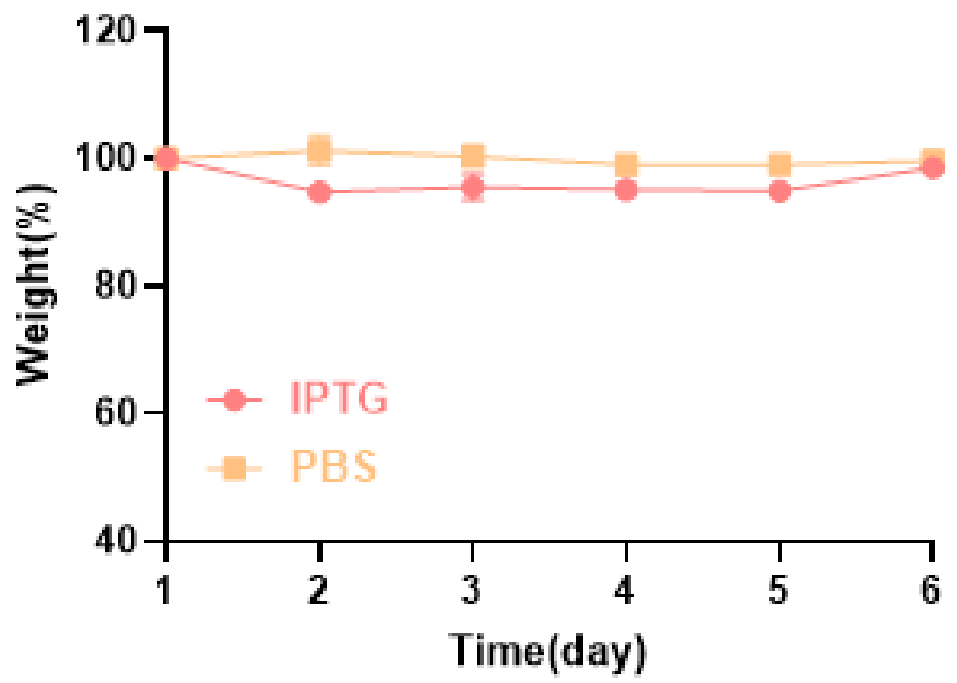


**Figure S10. In vivo safety testing of IPTG, Related to Figure 4.**

Body weight variation in mice after oral administration of IPTG. The body weight of mice over six days showed no significant difference in IPTG and PBS groups, demonstrating the safety of IPTG.

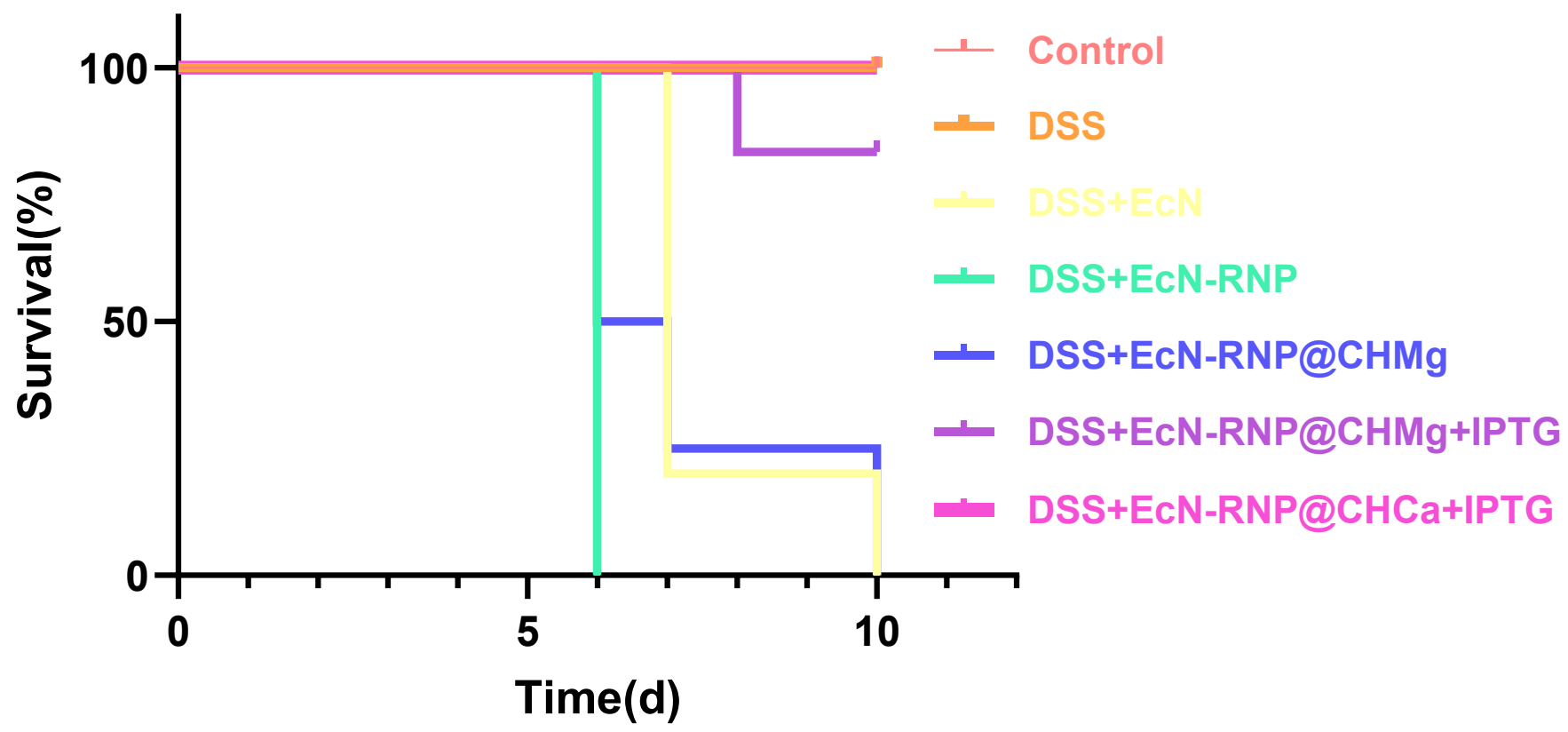


**Figure S11. Survival curves, Related to Figure 4.**

The results show that mice in groups except control, EcN-RNP@HCCa + IPTG and EcN-RNP@HCMg + IPTG died progressively.

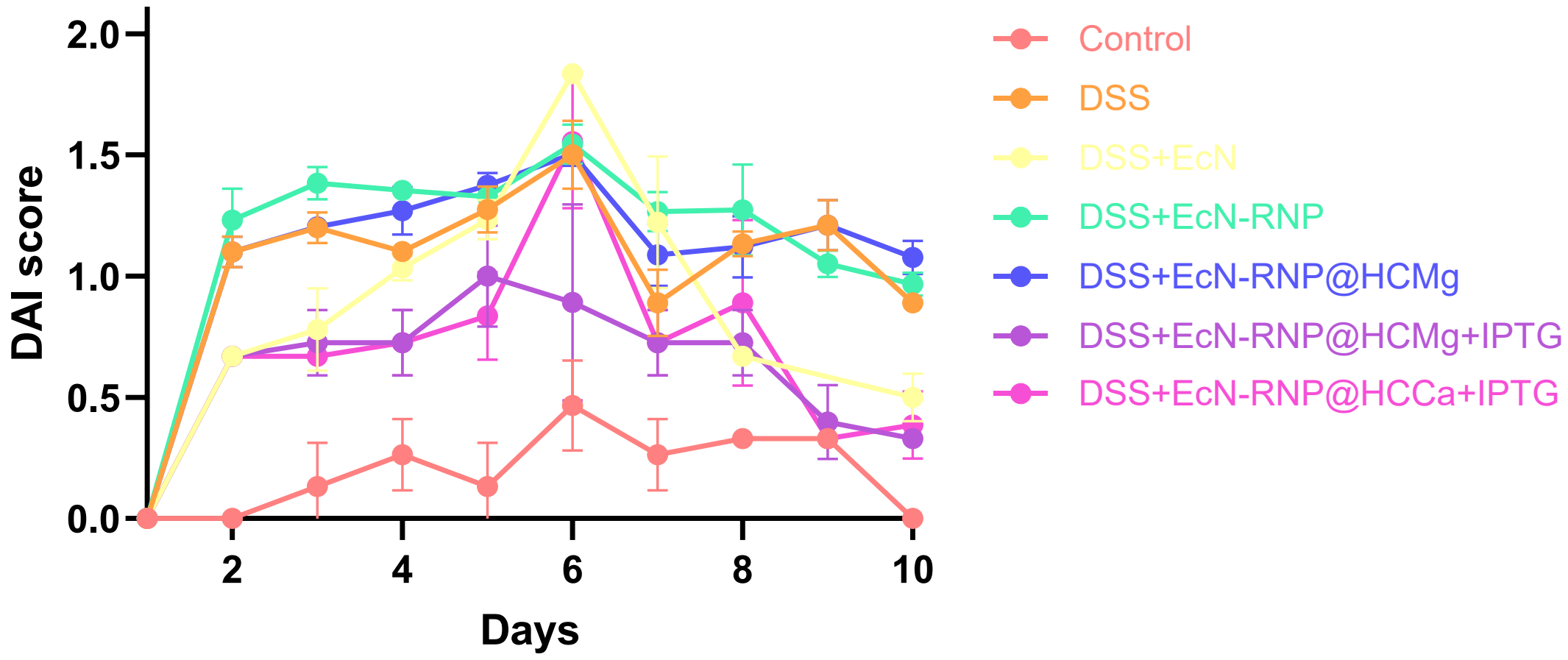


**Figure S12. DAI scores variation curve, Related to Figure 4.**

The DAI scores variation curve during the 10-day monitoring period. The DAI scores of mice showed a consistent increase in all groups after DSS treatment. However, these scores reduced in mice with oral administration of EcN-RNP@HCMg + IPTG, leading to a gradual normalization towards control group levels.

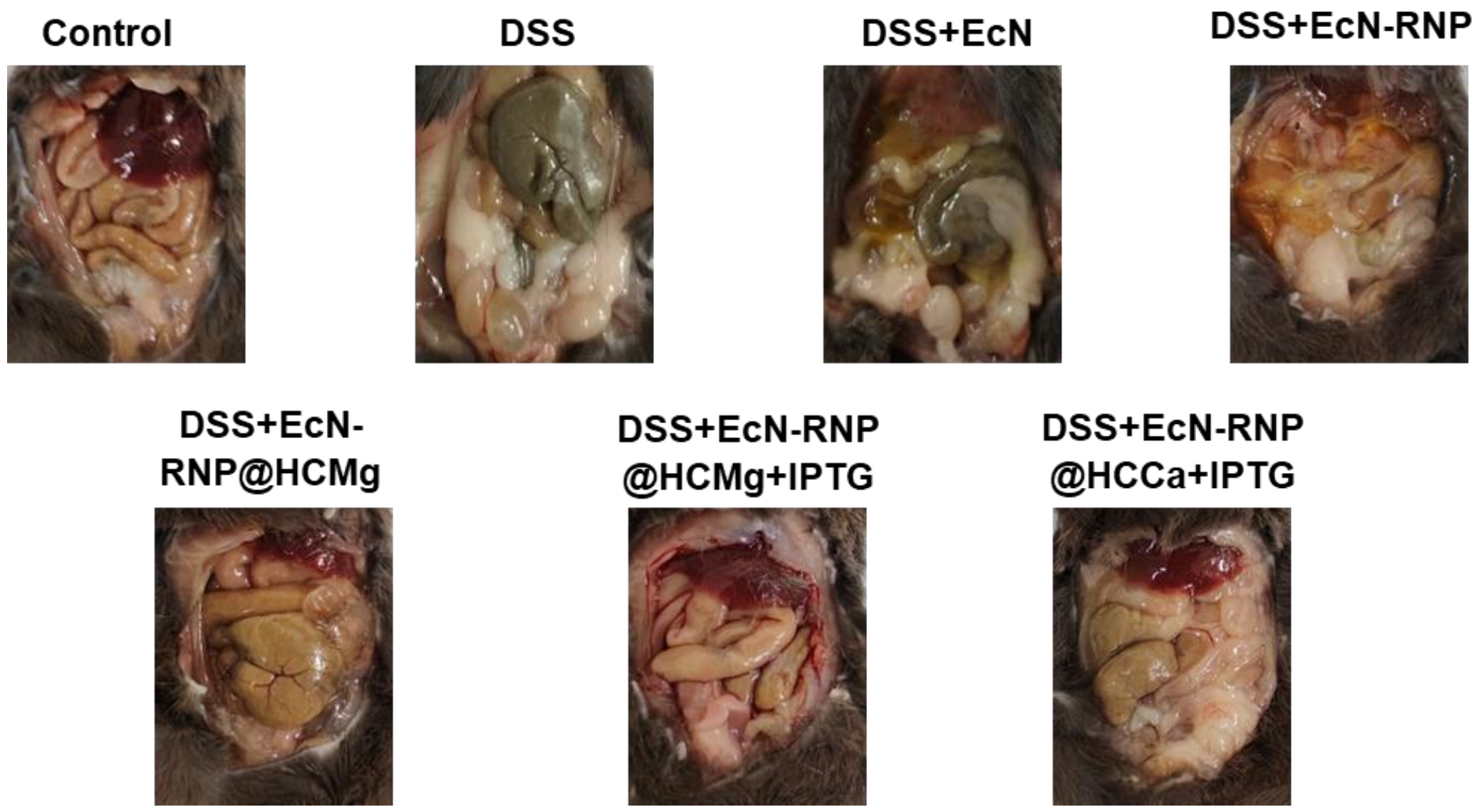


**Figure S13. Macroscopic pictures of abdominal condition, Related to Figure 4.**

Macroscopic pictures of abdominal condition from each group. Colons in mice exhibited tissue adhesions and irregular morphology after DSS treatment, whereas these conditions were alleviated in mice treated with EcN-RNP@HCMg + IPTG.

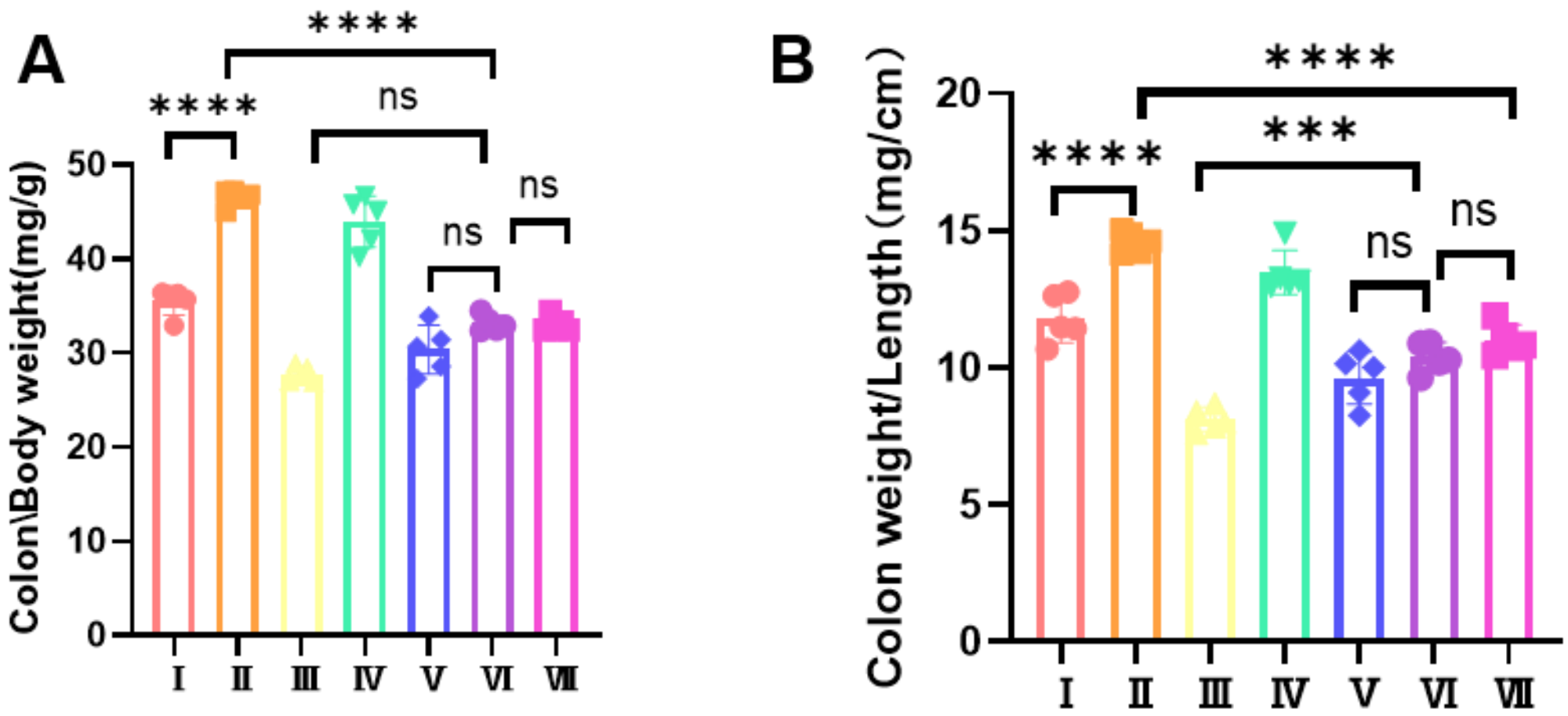


**Figure S14. Colon weight and length characteristics, Related to Figure 4.**

Colon weight and length characteristics. A) Colon /body weight ratio. B) Colon weight/length ratio. I: PBS, II: DSS, III:DSS+EcN, IV:DSS+EcN-RNP, V: DSS+EcN-RNP@HCMg, VI: DSS+EcN-RNP@ HCMg+IPTG, VII: DSS+EcN-RNP@HCCa+IPTG. The colon/body weight and colon weight/length ratio of mice significantly increased following DSS treatment, while these indices of mice treated with EcN-RNP@HCMg+IPTG reduced. Data are presented as mean ± SD, ns: no significance, *p < 0.05, **p < 0.01 and ***p < 0.001 calculated using Student's test or one-way ANOVA.

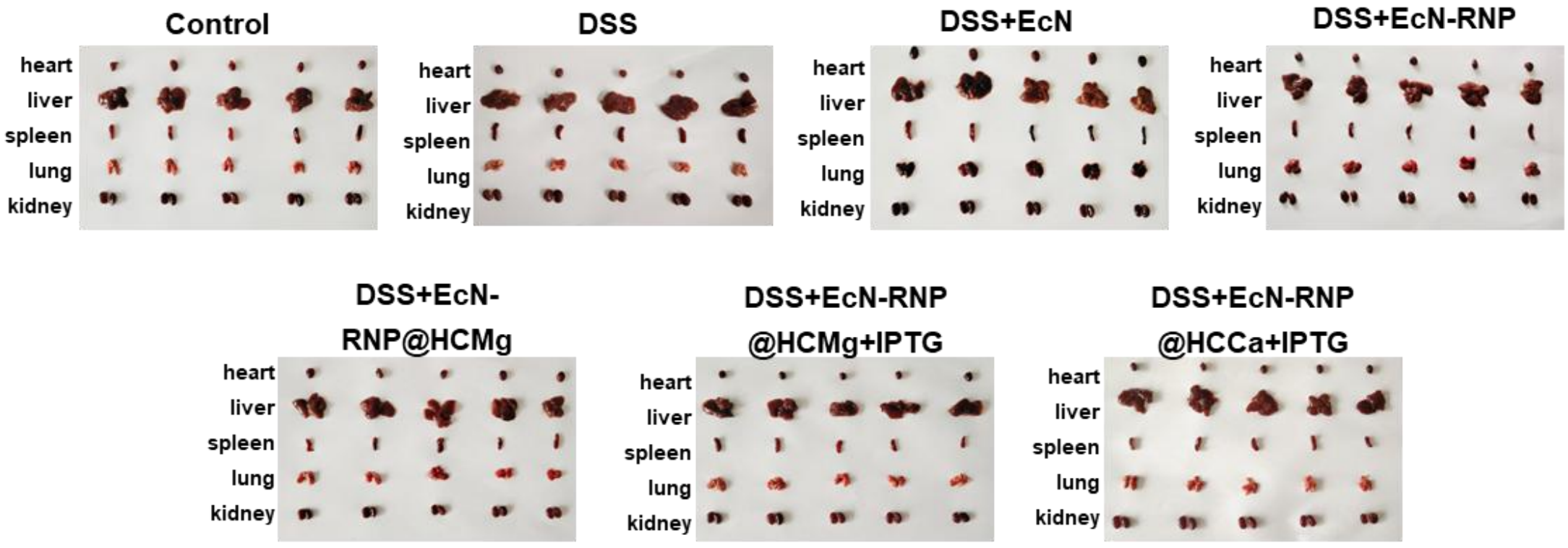


**Figure S15. Major organ pictures, Related to Figure 4.**

Macroscopic pictures of heart, liver, spleen, lung and kidney (another organ as there are five arrays from each group.

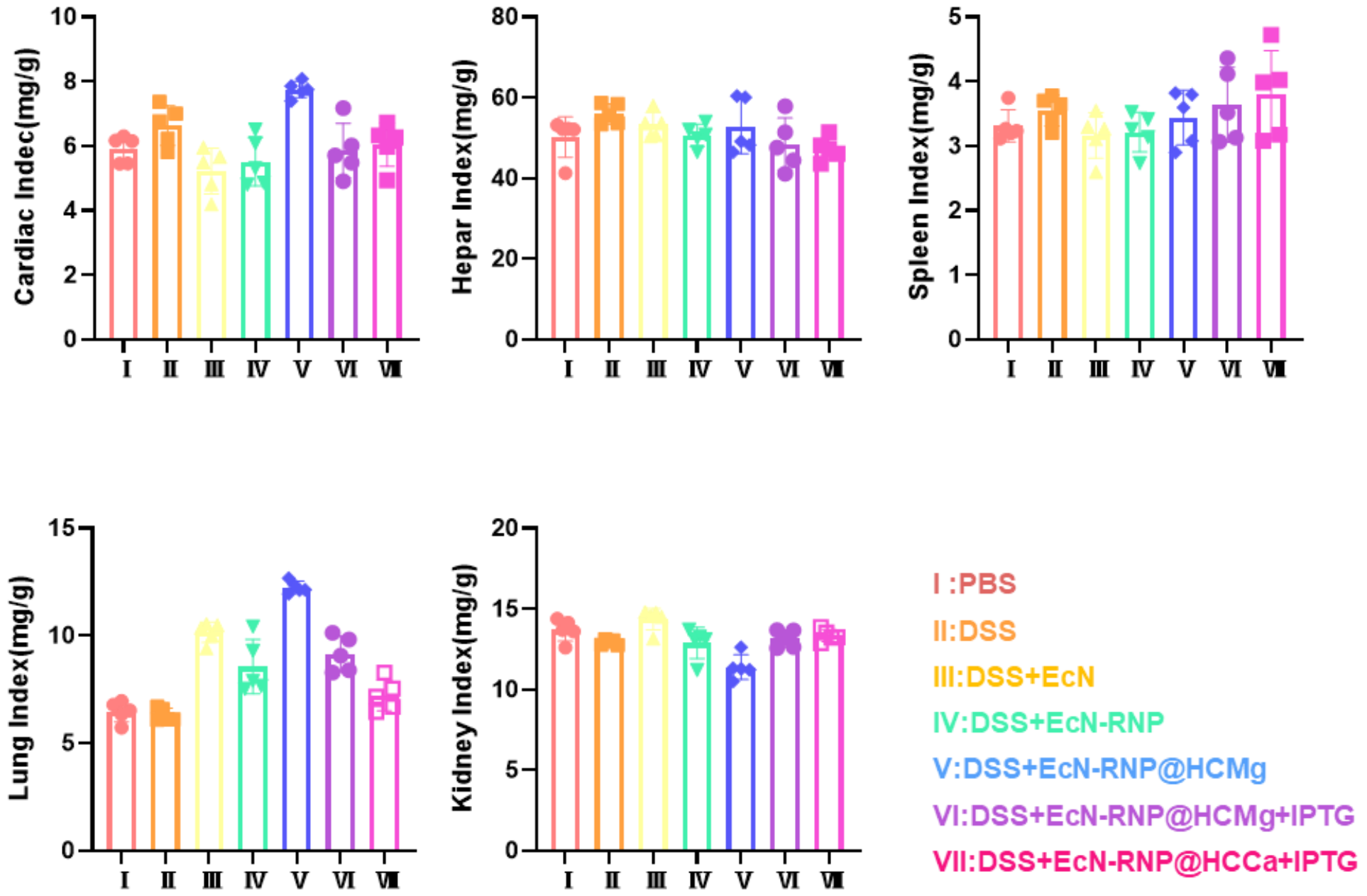


**Figure S16. Organ indices of heart, liver, spleen, lung, and kidney, Related to Figure 4.** The results show no significant differences in organ indices among groups, indicating that samples did not adversely affect mice organs. I: PBS, II: DSS, III: DSS+EcN, IV: DSS+EcN-RNP, V: DSS+EcN-RNP@HCMg, VI: DSS+EcN-RNP@HCMg+IPTG, VII: DSS+EcN-RNP@HCCa+IPTG.

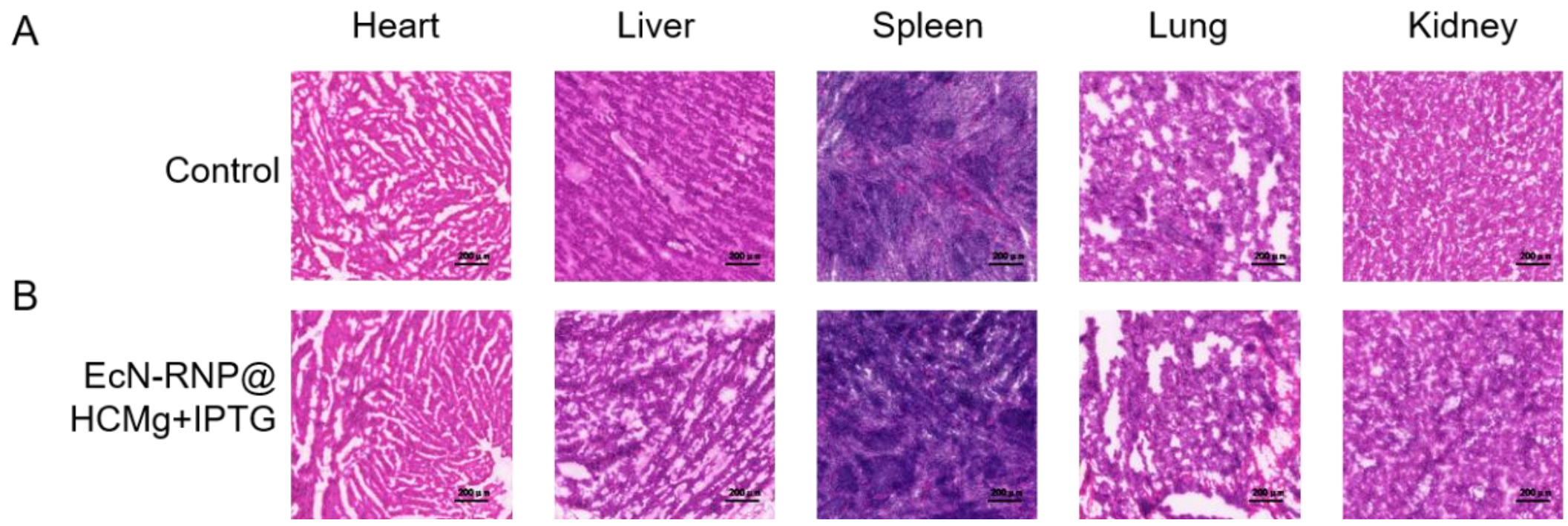


**Figure S17. H&E staining of major organs, Related to Figure 4.**

H&E staining of the heart, liver, spleen, lung and kidney tissues in mice from the control (A) and EcN-RNP@HCMg+IPTG (B) groups.

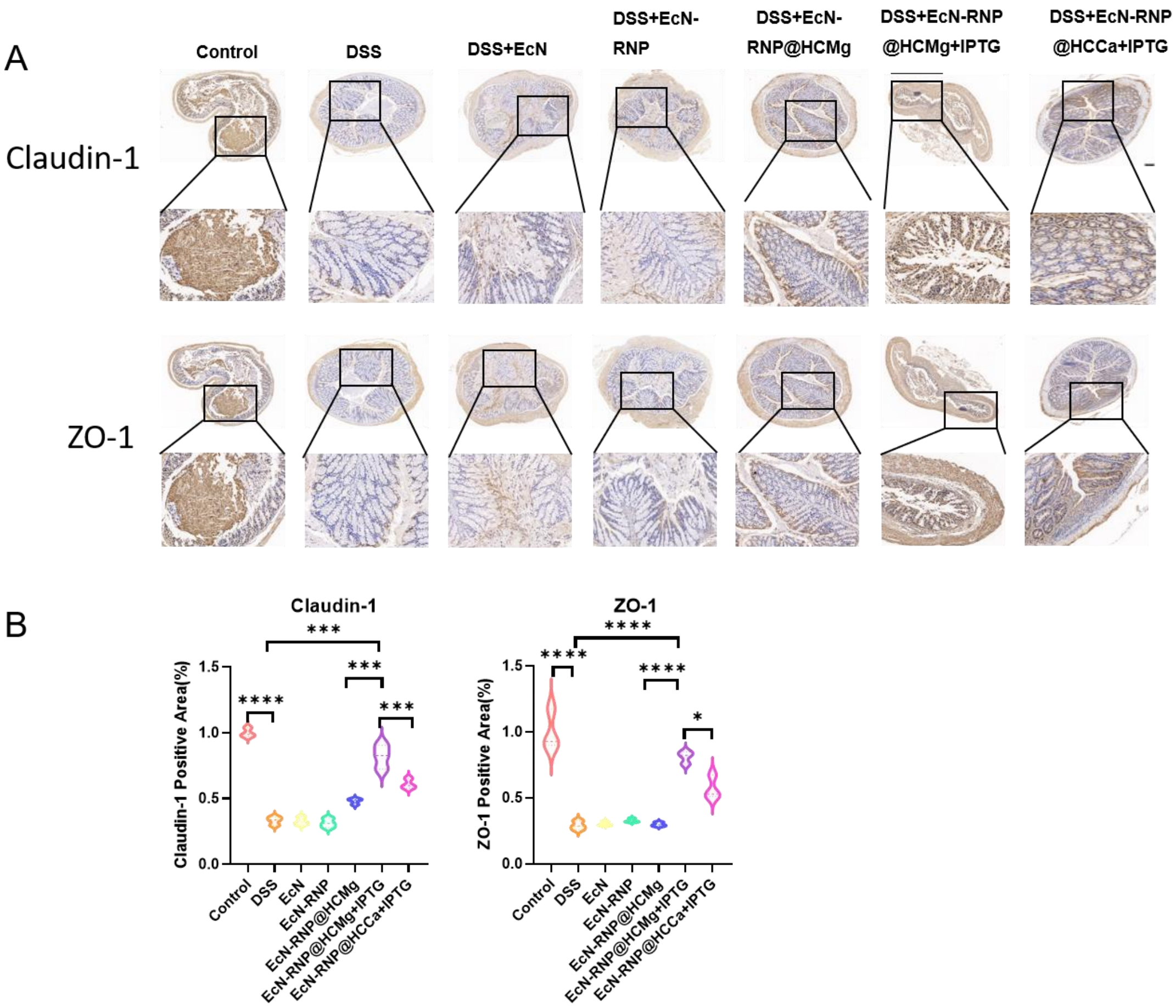


**Figure S18. Immunohistochemical staining of Claudin-1 and ZO-1, Related to Figure 4.**

Immunohistochemical staining images A) and statistical analysis B) of Claudin-1 and ZO-1 in colon tissues. The strong brown-stained intensity of Claudin-1 and ZO-1 exhibited in DSS + EcN-RNP@HCMg + IPTG group, indicating its capacity to repair the intestinal epithelial barrier. Data are presented as mean ± SD, ns: no significance, *p < 0.05, **p < 0.01 and ***p < 0.001 calculated using Student's test or one-way ANOVA.

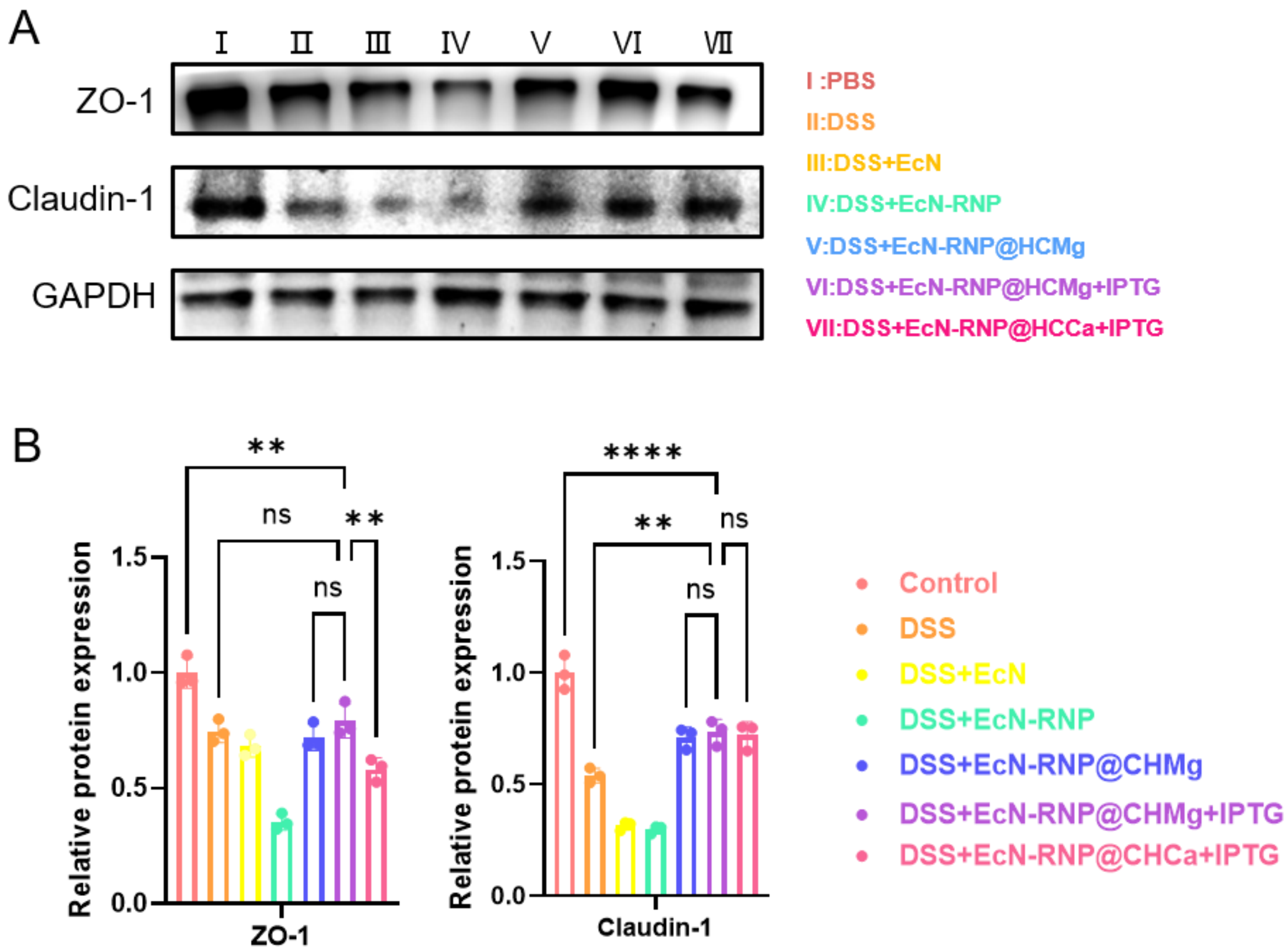


**Figure S19. Detection of intestinal epithelial barrier permeability, Related to Figure 4.**

Detection of intestinal epithelial barrier permeability. A) Expression levels and B) statistical analysis of ZO-1 and Claudin-1 in cells from colon tissues. Data are presented as mean ± SD, ns: no significance, *p < 0.05, **p < 0.01 and ***p < 0.001 calculated using Student's test or one-way ANOVA.

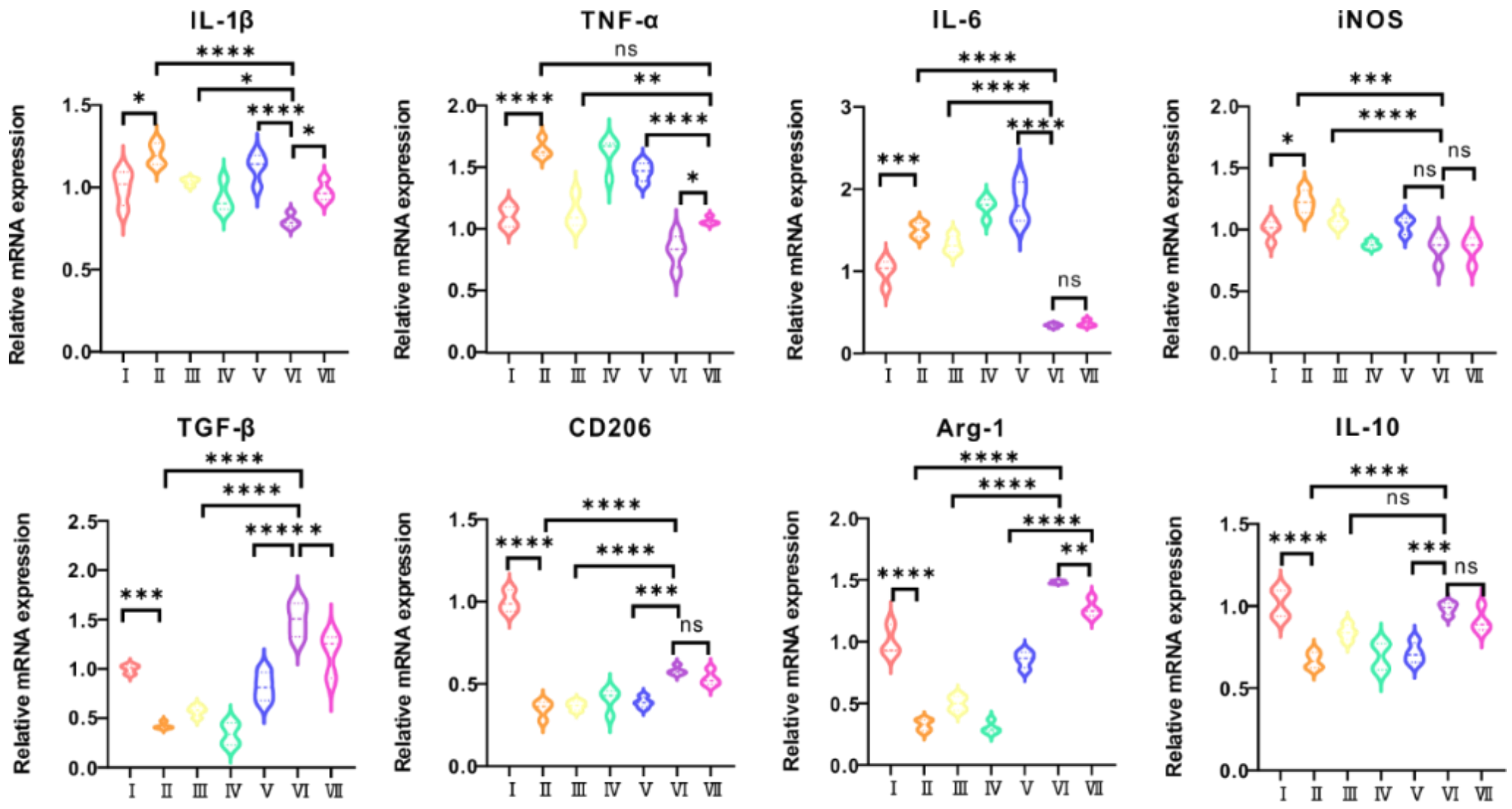


**Figure S20. The mRNA expressions analysis of inflammatory cytokine in colon tissues, Related to Figure 4.**

The mRNA expressions analysis of inflammatory cytokine in colon tissues. I: PBS, II: DSS, III:DSS+EcN, IV:DSS+EcN-RNP, V: DSS+EcN-RNP@HCMg, VI: DSS+EcN-RNP@ HCMg+IPTG, VII: DSS+EcN-RNP@HCCa+IPTG. Data are presented as mean ± SD, ns: no significance, $*p < 0.05$, $**p < 0.01$ and $***p < 0.001$ calculated using Student's test or one-way ANOVA.

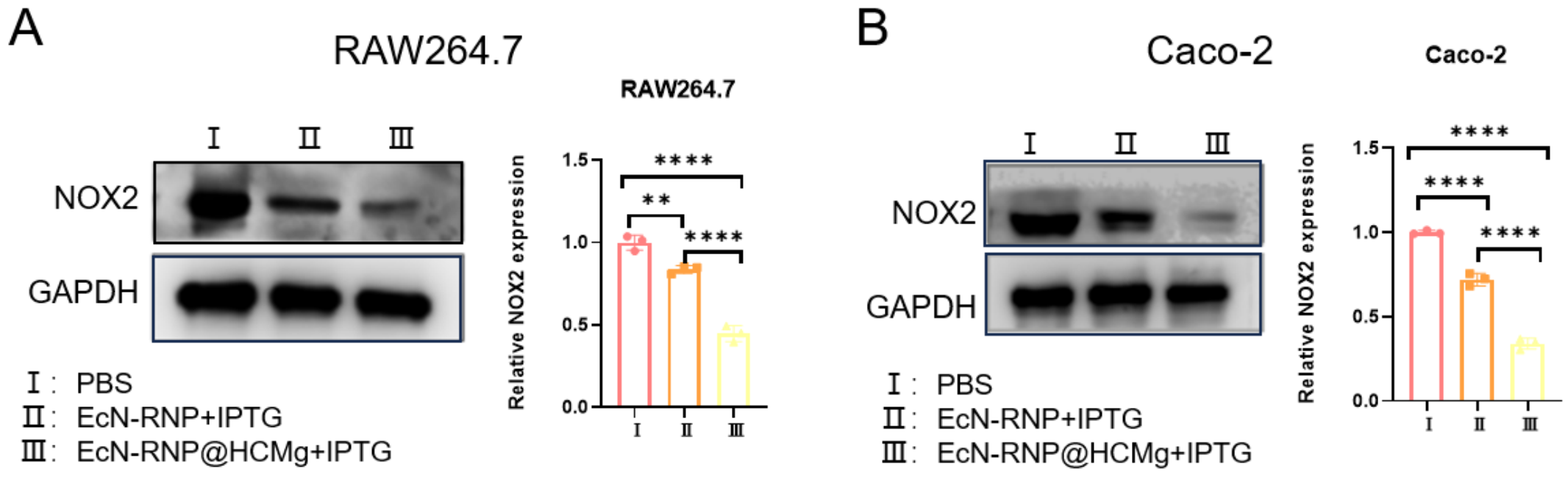


**Figure S21. IPTG induces CRISPR/Cas9 to inhibit the expression of NOX2, Related to Figure 5.**

Expression analysis of NOX2 in RAW264.7 cells A) and Caco-2 cells B) treated by EcN-RNP+IPTG and EcN-RNP@HCMg+IPTG. Data are presented as mean ± SD, ns: no significance, $*p < 0.05$, $**p < 0.01$ and $***p < 0.001$ calculated using Student's test or one-way ANOVA.

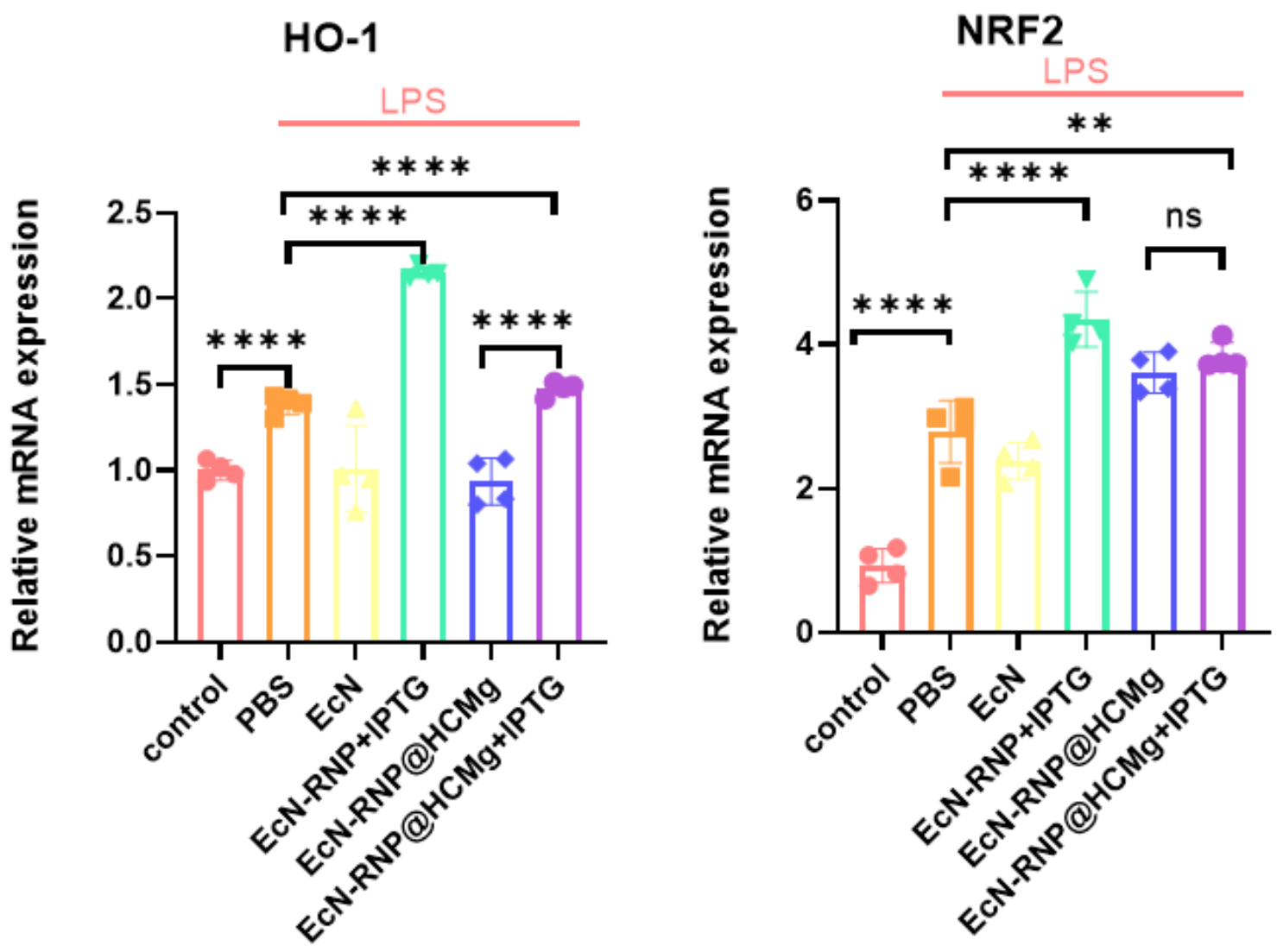


**Figure S22. The mRNA expression levels of HO-1 and NRF2, Related to Figure 5.**

mRNA expression levels of HO-1 and NRF2 in RAW 264.7 cells. The mRNA expression of HO-1 and NRF2 were upregulated in cells treated by EcN-RNP@HCMg+IPTG. Data are presented as mean ± SD, ns: no significance, *p < 0.05, **p < 0.01 and ***p < 0.001 calculated using Student's test or one-way ANOVA.

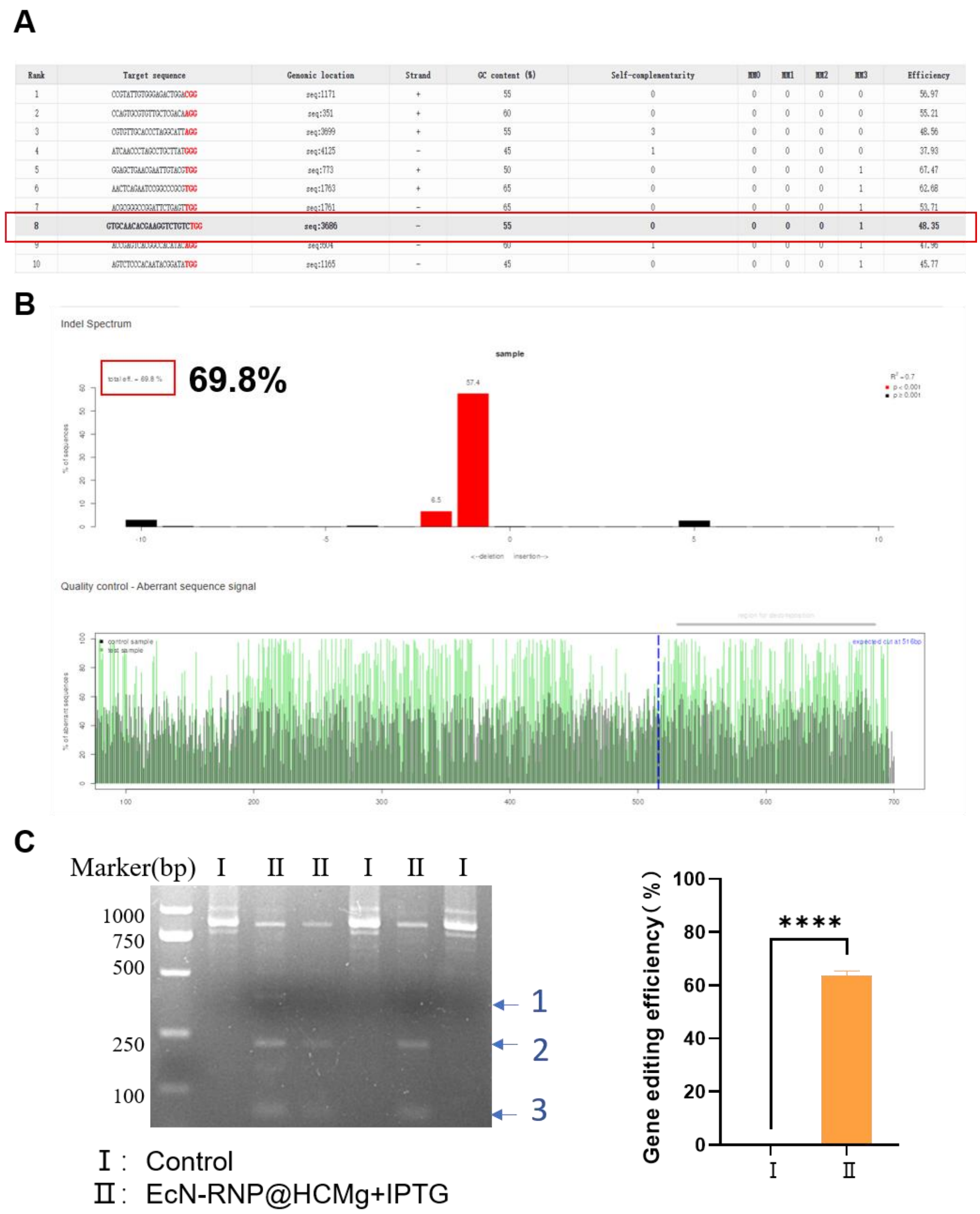

| Rank | Target sequence | Genomic location | Strand | GC content (%) | Self-complementarity | MM0 | MM1 | MM2 | MM3 | Efficiency |
|---|---|---|---|---|---|---|---|---|---|---|
| 1 | CCGTATTGTGGGAGACTGGACGG | seq:1171 | + | 55 | 0 | 0 | 0 | 0 | 0 | 56.97 |
| 2 | CCAGTGCGTGTTGCTCGACAAGG | seq:351 | + | 60 | 0 | 0 | 0 | 0 | 0 | 55.21 |
| 3 | CGTGTTGCACCCTAGGCATTAGG | seq:3699 | + | 55 | 3 | 0 | 0 | 0 | 0 | 48.56 |
| 4 | ATCAACCCTAGCCTGCTTATGGG | seq:4125 | - | 45 | 1 | 0 | 0 | 0 | 0 | 37.93 |
| 5 | GGAGCTGAACGAATTGTACGTGG | seq:773 | + | 50 | 0 | 0 | 0 | 0 | 1 | 67.47 |
| 6 | AACTCAGAATCCGGCCCGCGTGG | seq:1763 | + | 65 | 0 | 0 | 0 | 0 | 1 | 62.68 |
| 7 | ACGCGGGCCGGATTCTGAGTTGG | seq:1761 | - | 65 | 0 | 0 | 0 | 0 | 1 | 53.71 |
| 8 | GTGCAACACGAAGGTCTGTCTGG | seq:3686 | - | 55 | 0 | 0 | 0 | 0 | 1 | 48.35 |
| 9 | [illegible] | [illegible] | - | 60 | 1 | 0 | 0 | 0 | 1 | [illegible] |
| 10 | AGTCTCCCACAATACGGATATGG | seq:1165 | - | 45 | 0 | 0 | 0 | 0 | 1 | 45.77 |



**Figure S23. Analysis of genome editing efficiency, Related to Figure 5.**

A) *In silico* prediction of the gene editing efficiency of the selected sgRNA at the target locus. B) Mutation efficiency detected by TIDE analysis in cells isolated from colonic tissues of mice treated with EcN-RNP@HCMg+IPTG. C) The indel mutation frequency of NOX2 determined by T7EI assay in mouse colonic tissues following EcN-RNP@HCMg+IPTG treatment.

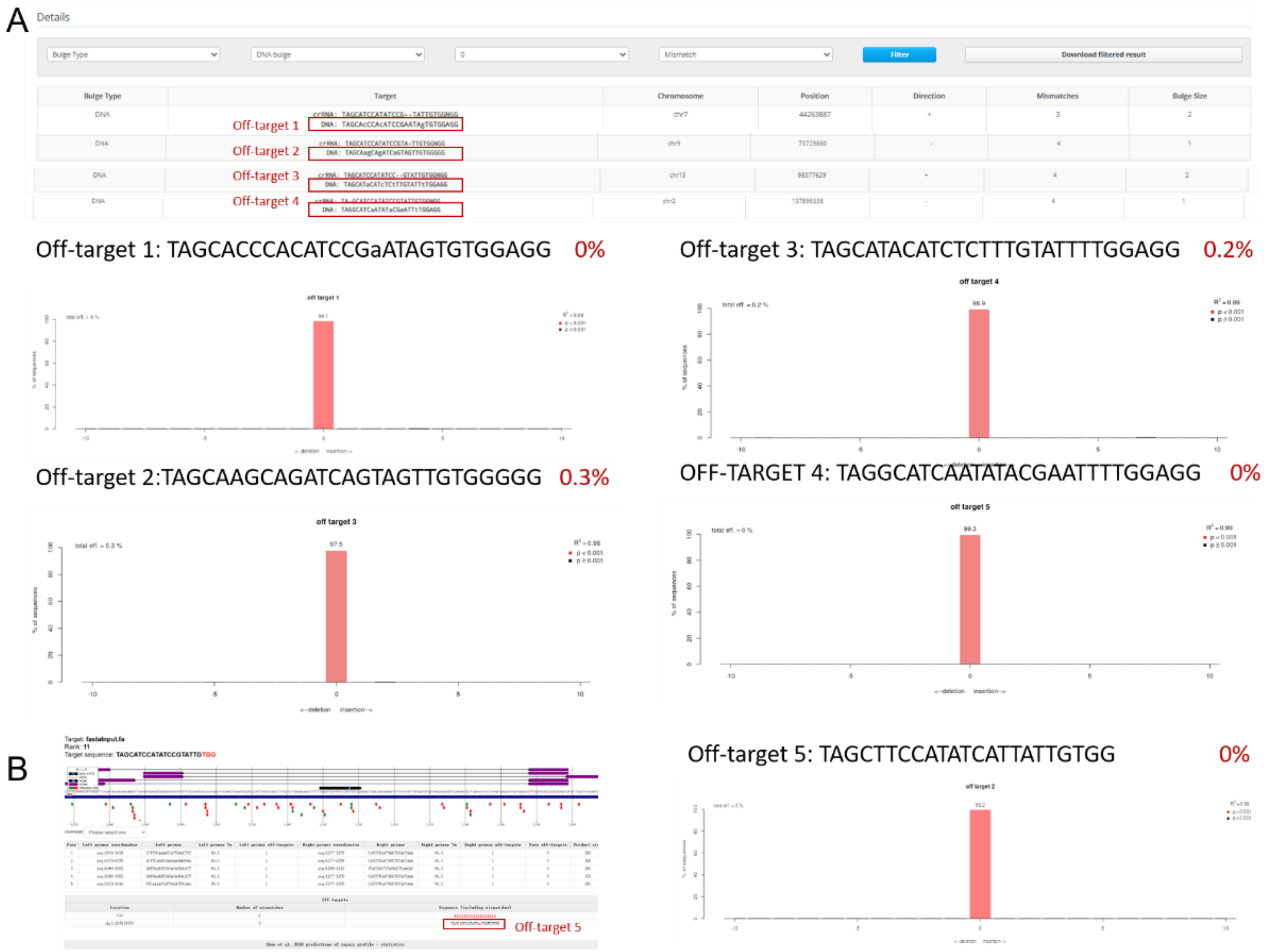


**Figure S24. Analysis of the off-target rate, Related to Figure 5.**

A) Five off-targets of EcN-RNP@HCMg+IPTG were predicted by two computational software programs. B) Analysis of the corresponding mutation frequency of the five off-targets.

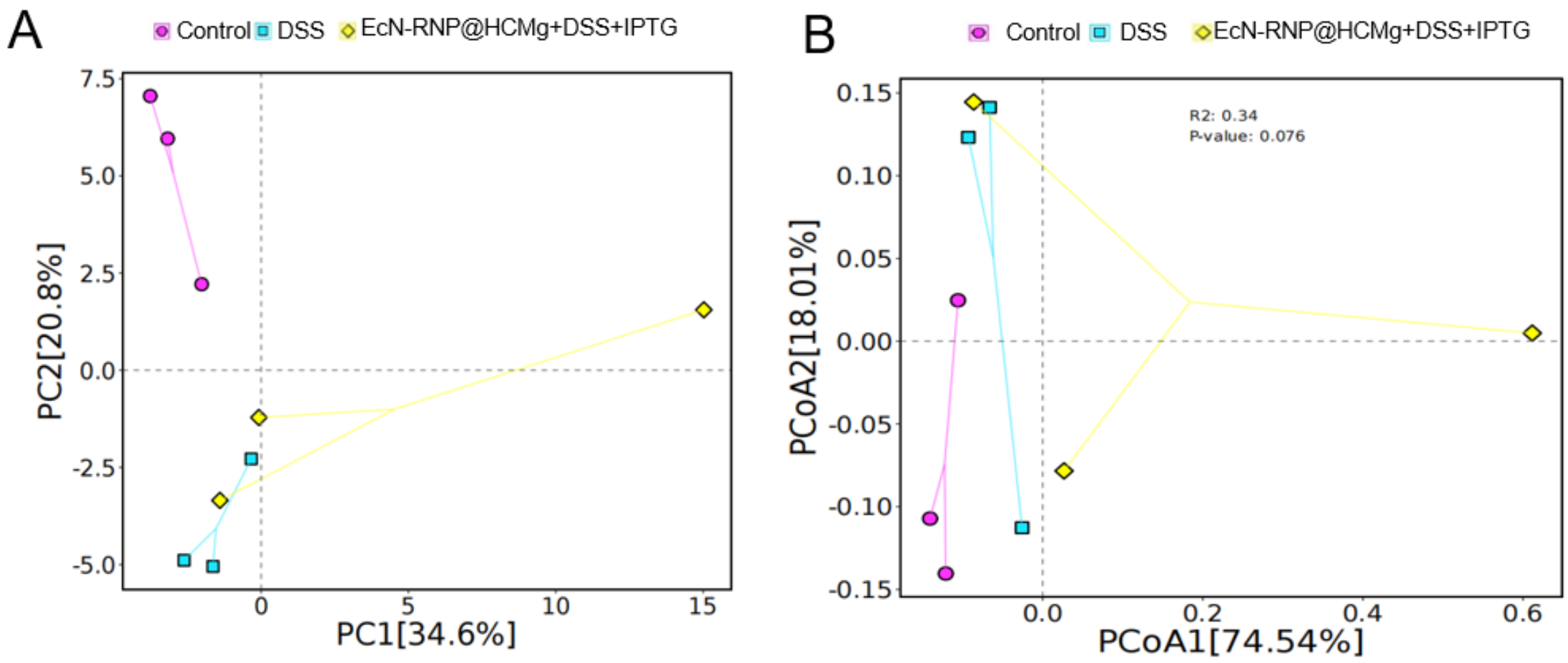


**Figure S25. Beta diversity analysis, Related to Figure 7.**

The results show that the microbial community composition in EcN-RNP@HCMg group exerts a closer relationship with the healthy control group, compared with the DSS group.

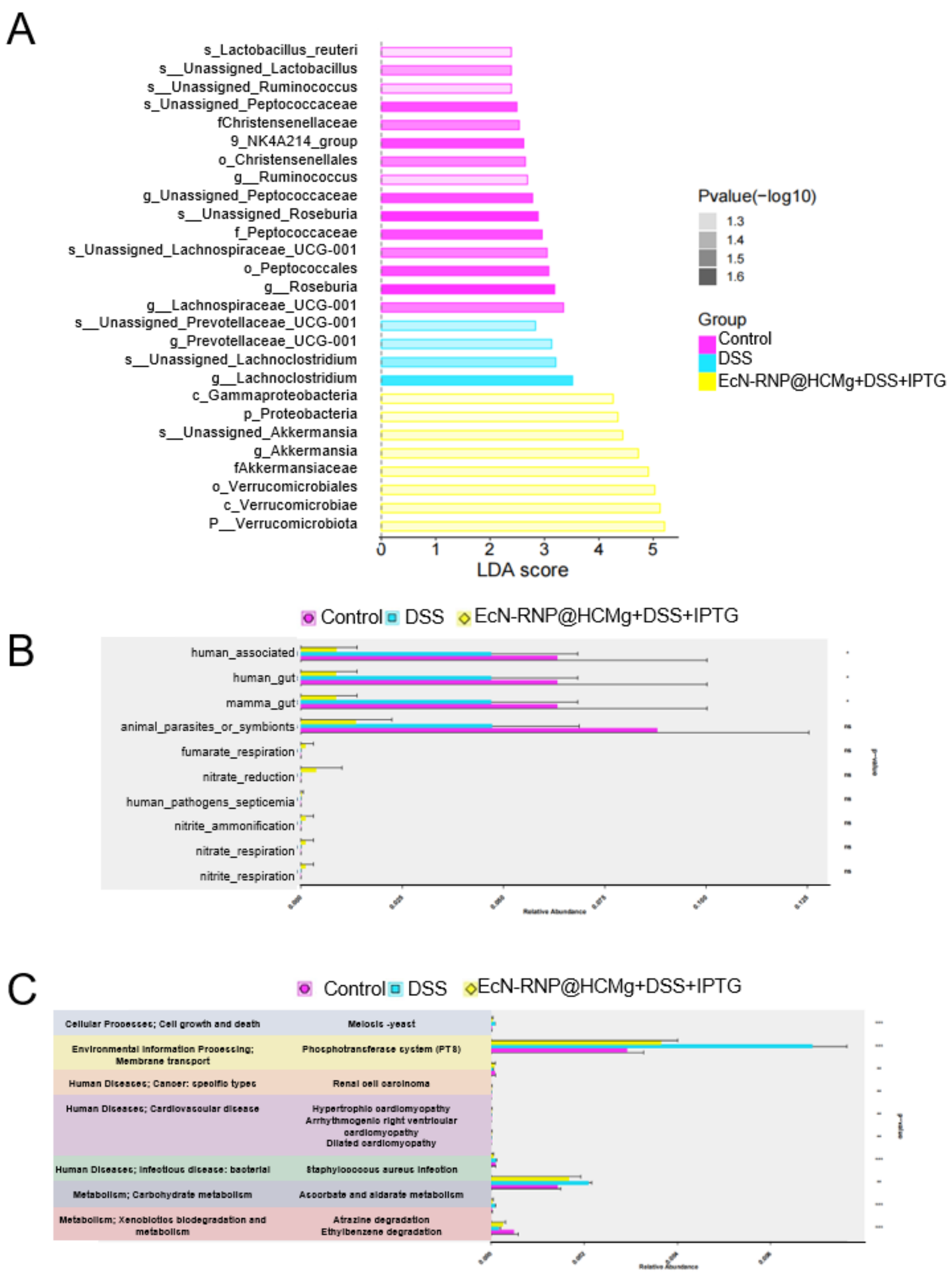


**Figure S26. Gut microbiota functional analysis, Related to Figure 7.**

Functional analysis of bacteria in the colon tissues were performed and the results show that the functions related to the intestines have been restored treated by EcN-RNP@HCMg+IPTG.

**Table S1.**

**Table S1. Primers sequence, Related to STAR Methods.**

| Name | Sequence |
|---|---|
| β-actin(F) | GGCTGTATTCCCCTCCATCG |
| β-actin(R) | CCAGTTGGTAACAATGCCATGT |
| iNOS(F) | GTTCTCAGCCCAACAATACAAGA |
| iNOS(R) | GTGGACGGGTCGATGTCAC |
| IL-1β(F) | GCAACTGTTCCTGAACTCAAC |
| IL-1β(R) | ATCTTTTGGGGTCCGTCAACT |
| IL-10(F) | GCTCTTACTGACTGGCATGA |
| IL-10(R) | CGCAGCTCTAGGAGCATGTG |
| TGF-β2(F) | CTTCGACGTGACAGACGCT |
| TGF-β2(R) | GCAGGGGCAGTGTAAACTTATT |
| IL-6(F) | TAGTCCTTCCTACCCCAATTTCC |
| IL-6 (R) | TTGGTCCTTAGCCACTCCTTC |
| TNF-α(F) | CCCTCACACTCAGATCATCTTCT |
| TNF-α(R) | GCTACGACGTGGGCTACAG |
| Arg-1(F) | CTCCAAGCCAAAGTCCTTAGAG |
| Arg-1(R) | AGGAGCTGTCATTAGGGACATC |
| CD206(F) | CTCTGTTCAGCTATTGGACGC |
| CD206(R) | CGGAATTTCTGGGATTCAGCTTC |
| Gp91$^{phox}$ cell(F) | CCCCAAGGTATCCAAGTT |
| Gp91$^{phox}$ cell(R) | AAAGGGTCCATCAACTGC |
| Gp91$^{phox}$ bacteria(F) | CGCCAGGGTTTTCCCAGTCACGACGTT |
| Gp91$^{phox}$ bacteria(R) | ATGCAGCTGGCACGACAGGTTTCCCGA |
| Cas9(F) | CAAACAGCGCACTTTCGACAATGGAAG |
| Cas9(R) | TGTTCCTTAAGGATTTGGGACCCCAGT |
| Target 1(F) | CTGTATGTGGCCGTGACTCG |
| Target 1(R) | CCTTTCCTGCATCTGGGTCT |
| Off-target 1(F) | CCTCGCCTGGGAGATAGGAG |
| Off-target 1(R) | GCACGGGGATCAGAGGTTTT |
| Off-target 2(F) | TGAGCCTTTTCAGATTTCTGTTCC |
| Off-target 2(R) | ACAAGGATGATGCCTATGGCT |
| Off-target 3(F) | TCTACTCTTGCTCTGCCATACATT |
| Off-target 3(R) | AGCAATATGATGCGCAATCCC |
| Off-target 4(F) | TGACGCCCCCTTGTTTTTCT |
| Off-target 4(R) | CCACCCGTAGGCCTTCATAC |
| Off-target 5(F) | TCTCAGTTTCAGGCCTTCGG |
| Off-target 5(R) | ACATGCAAAGTCGCAAAGCC |
